# Evaluation of the Inhibitory Effects of Pyridylpyrazole Derivatives on LPS-Induced PGE_2_ Productions and Nitric Oxide in Murine RAW 264.7 Macrophages

**DOI:** 10.3390/molecules26216489

**Published:** 2021-10-27

**Authors:** Mahmoud M. Gamal El-Din, Mohammed I. El-Gamal, Young-Do Kwon, Su-Yeon Kim, Hee-Soo Han, Sang-Eun Park, Chang-Hyun Oh, Kyung-Tae Lee, Hee-Kwon Kim

**Affiliations:** 1 National Research Centre, Pharmaceutical and Drug Industries Research Division, Dokki-Giza 12622, Egypt; dr.m.g.eldin@hotmail.com; 2 Department of Medicinal Chemistry, College of Pharmacy, University of Sharjah, Sharjah 27272, United Arab Emirates; drmelgamal2002@gmail.com; 3 Sharjah Institute for Medical Research, University of Sharjah, Sharjah 27272, United Arab Emirates; 4 Department of Medicinal Chemistry, Faculty of Pharmacy, Mansoura University, Mansoura 35516, Egypt; 5 Molecular Imaging & Therapeutic Medicine Research Center, Department of Nuclear Medicine, Jeonbuk National University Medical School and Hospital, 20 Geonji-ro, Deokjin-gu, Jeonju 54907, Korea; gskydgo@gmail.com; 6 Research Institute of Clinical Medicine, Jeonbuk National University-Biomedical Research Institute, Jeonbuk National University Hospital, 20 Geonji-ro, Deokjin-gu, Jeonju 54907, Korea; 7 Department of Pharmaceutical Biochemistry, Kyung Hee University, Seoul 02447, Korea; dlstm4@gmail.com (S.-Y.K.); heesu3620@daum.net (H.-S.H.); qkrtkddms0930@naver.com (S.-E.P.); 8 Department of Life and Nanopharmaceutical Sciences, Graduate School, Kyung Hee University, 26, Kyungheedae-ro, Seoul 02447, Korea; 9 Center for Biomaterials, Korea Institute of Science and Technology, P.O. Box 131, Cheongryang, Seoul 130-650, Korea; choh@kist.re.kr

**Keywords:** amide, anti-inflammatory, COX-2, iNOS, NO, PGE_2_, pyrazole

## Abstract

A series of thirteen triarylpyrazole analogs were investigated as inhibitors of lipopolysaccharide (LPS)-induced prostaglandin E_2_ (PGE_2_) and nitric oxide (NO) production in RAW 264.7 macrophages. The target compounds **1a**–**m** have first been assessed for cytotoxicity against RAW 264.7 macrophages to determine their non-cytotoxic concentration(s) for anti-inflammatory testing to make sure that the inhibition of PGE_2_ and NO production would not be caused by cytotoxicity. It was found that compounds **1f** and **1m** were the most potent PGE_2_ inhibitors with IC_50_ values of 7.1 and 1.1 μM, respectively. In addition, these compounds also showed inhibitory effects of 11.6% and 37.19% on LPS-induced NO production, respectively. The western blots analysis of COX-2 and iNOS showed that the PGE_2_ and NO inhibitory effect of compound **1m** are attributed to inhibition of COX-2 and iNOS protein expression through inactivation of p38.

## 1. Introduction

Inflammation is considered as a part of our body’s defense mechanisms against invasive organisms. It represents an attempt to get rid of such harmful organisms through releasing antibacterial or antiviral from cells close to it to help the body fight against infection [1]. In addition, it enhances injured tissue healing facilitating the return of the cells to their normal conditions. Despite these beneficial effects, it could have harmful effects triggering a list of disorders such as cardiovascular disorders [2], tumors [3], inflammatory bowel syndrome [4], arthritis [5], pulmonary disorders [6], Alzheimer’s [7], etc.

In order to treat inflammation, it is crucial to understand the role of inflammatory mediators that directly contribute to inflammatory responses. Inflammatory mediators arise from plasma proteins or some types of cells such as mast cells, platelets, neutrophils, monocytes, and macrophages. They are triggered by bacterial toxins or host cell proteins. The inflammatory mediators bind to particular receptors on the target cells and enhance vascular permeability and neutrophil chemotaxis, induce smooth muscle contraction, directly affect enzymatic activity, produce pain, or induce oxidative damage. The majority of these chemical mediators have short lives but produce harmful effects [1]. The inflammatory chemical mediators are exemplified by vasoactive amines (e.g., histamine and 5-HT), eicosanoids (e.g., prostaglandins and leukotrienes), and cytokines (e.g., tumor necrosis factor (TNF) and interleukin-1 (IL-1)).

Cyclooxygenase-2 (COX-2) converts arachidonic acid into PGE_2_, which is the mediator of inflammation [8]. Limiting PGE_2_ production *via* inhibition of COX-2 protein expression and/or enzymatic activity is another useful approach for the treatment of inflammation. Moreover, nitric oxide (NO) has another considerable contribution to inflammation development (although it could produce anti-inflammatory effect under other normal physiological conditions) [9,10,11]. On the other hand, it acts as a proinflammatory mediator to induce localized inflammatory response due to elevated secretion in cases of abnormal conditions. Inducible nitric oxide synthase (iNOS) enzyme forms NO in case of inflammation. NO produces localized vasodilation at the site of inflammation, leading to edema [12]. Therefore, Similar to PGE_2_ production inhibition, decreasing NO production *via* iNOS enzymatic activity inhibition, and/or iNOS protein expression inhibition could be a beneficial avenue for the management of inflammation.

**Figure 1:**
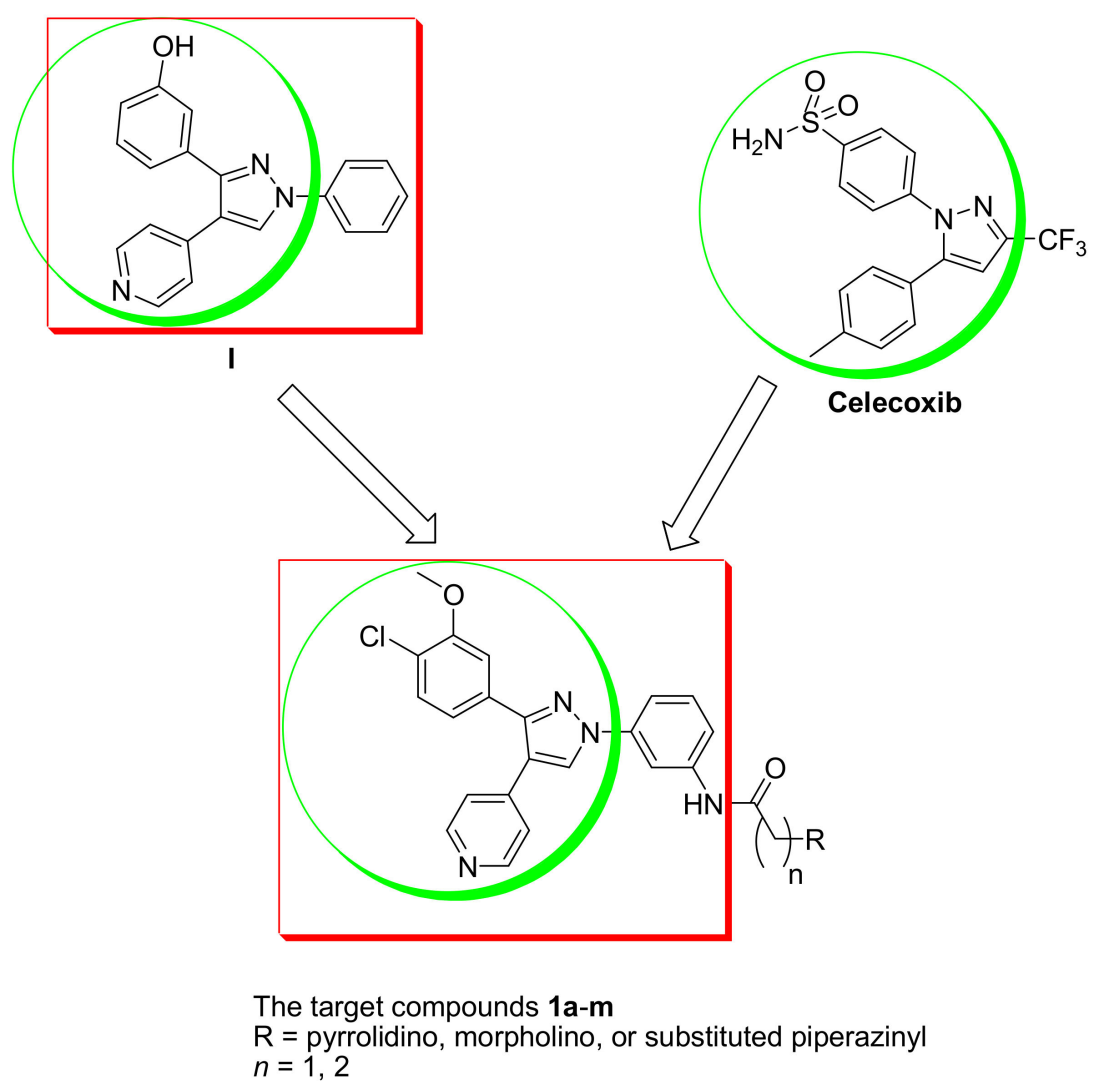
Structures of the lead compound **I** [18], celecoxib, and the target compounds **1a**–**m**.

Many substituted pyrazole derivatives have been recently reported to possess anti-inflammatory activity [13,14,15,16]. In our study, we evaluated a series of substituted pyrazole derivatives with a structural likeness to celecoxib, a pyrazole-based anti-inflammatory agent (Figure 1) as inhibitors of LPS-induced NO and PGE_2_ productions. Vicinal diarylheterocycles such as celecoxib have been reported as COX-2-inhibiting anti-inflammatory agents. The presence of vicinal diarylpyrazole scaffold in the structures of our target compounds encouraged us to investigate their anti-inflammatory activity. Our target compounds **1a**–**m** were previously reported as antiproliferative agents [17]. Moreover, compound **I** (Figure 1) possessing triarylpyrazole nucleus has been reported as inhibitor of PGE_2_ and NO release [18].

## 2. Results and Discussion

### 2.1. Chemistry

**Scheme 1:**
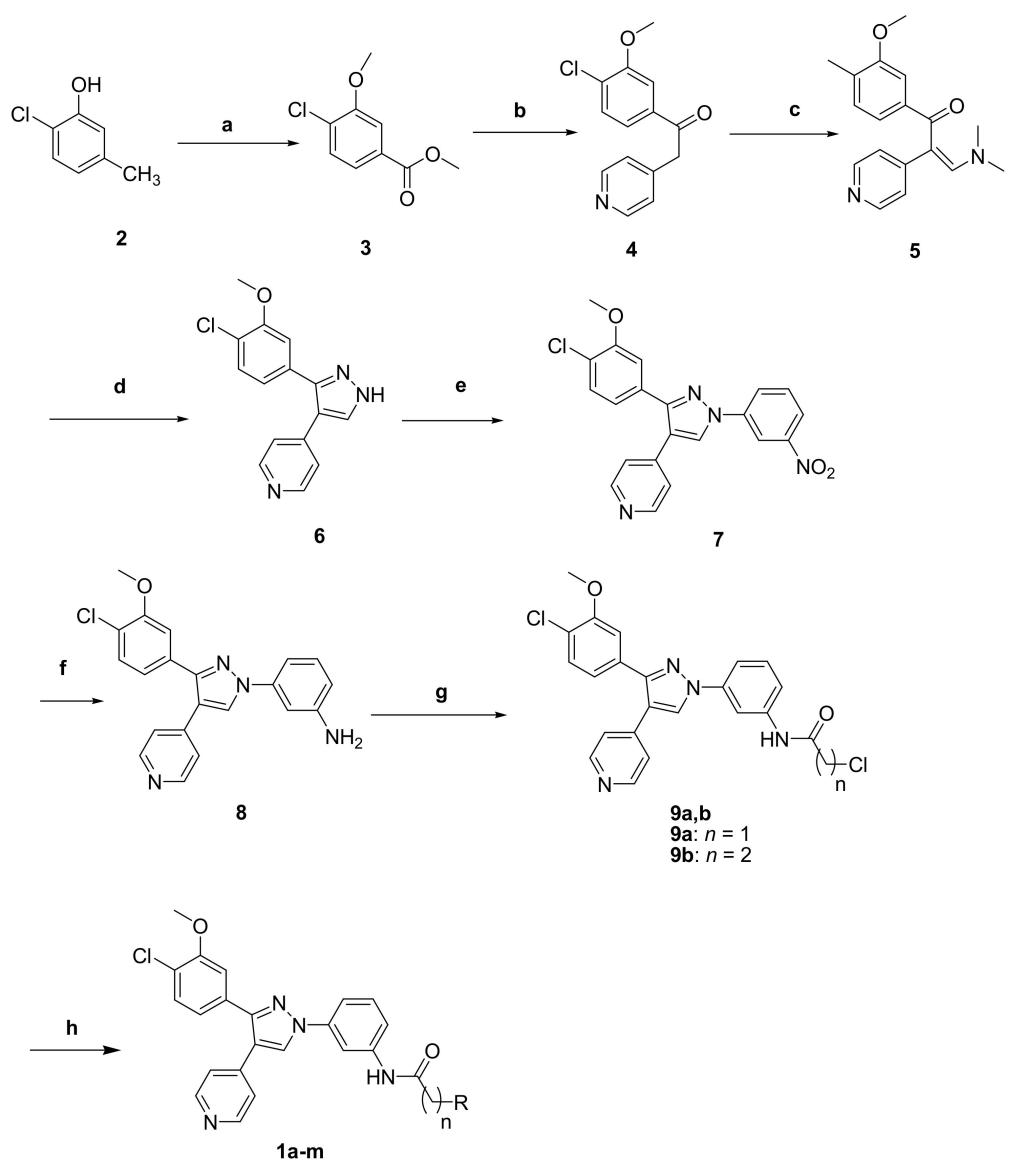
Reagents and conditions: (**a**) (i) (CH_3_)_2_SO_4_, K_2_CO_3_, acetone, reflux, 1 h, 95%; (ii) KMnO_4_, C_5_H_5_N, H_2_O, 50 °C, 24 h, then rt, 13 h, 90%; (iii) acetyl chloride, CH_3_OH, rt, 15 h, 85%; (**b**) 4-picoline, LiHMDS, THF, rt, overnight, 45%; (**c**) (i) DMF-DMA, rt, 18 h; (**d**) hydrazine monohydrate, C_2_H_5_OH, rt, overnight, 81%; (**e**) 1-iodo-4-nitrobenzene, K_2_CO_3_, CuI, L-proline, DMSO, 90 °C, 8 h, 86%; (**f**) H_2_, Pd/C, THF, rt, 2 h, 86%; (**g**) chloroacetyl chloride, or chloropropionyl chloride, TEA, CH_2_Cl_2_, −10 °C, 15 min, 65%; (**h**) appropriate amine derivative, TEA, CH_2_Cl_2_, rt, 1 h, 46–71%.

The final compounds **1a**–**m** were synthesized *via* the pathway demonstrated in Scheme 1. 2-Chloro-5-methylphenol (**2**) was reacted with dimethyl sulfate/potassium carbonate to obtain methoxy derivative. The methyl group was then oxidized to carboxylic acid by potassium permanganate to 4-chloro-3-methoxy-benzoic acid. Esterification of the resulting acid by methanol and acetyl chloride yielded the corresponding methyl ester **3**. Compound **3** was activated using a strong base; lithium bis(trimethylsilyl)amide (LiHMDS) followed by slow addition of 4-picoline gave the pyridine ketide intermediate **4**. The reaction of compound **4** with dimethylformamide dimethylacetal (DMF-DMA) produced compound **5**. After that, adding hydrazine monohydrate yielded the pyrazolyl intermediate **6**. Interaction of compound **6** with *meta*-iodonitrobenzene at 90 °C in dimethyl sulfoxide gave the *meta*-nitrophenyl intermediate **7**. Reduction of the NO_2_ group of **7** using Pd/C and hydrogen gas produced amino compound **8**. Interaction of the amino intermediate **8** with chloroacetyl chloride or chloropropionyl chloride produced the corresponding amide intermediates **9a,b**, respectively. Interaction of the terminal alkyl halide group of compounds **9a,b** with (substituted) alicyclic amines gave the target compounds **1a**–**m** [17]. The detailed experimental procedures and the spectral analysis charts are shown in the Supplementary File. Structures of compounds **1a**–**m** and their cell viability results against RAW 264.7 cells are shown in Table 1.

### 2.2. Biological Evaluation

**Figure 2:**
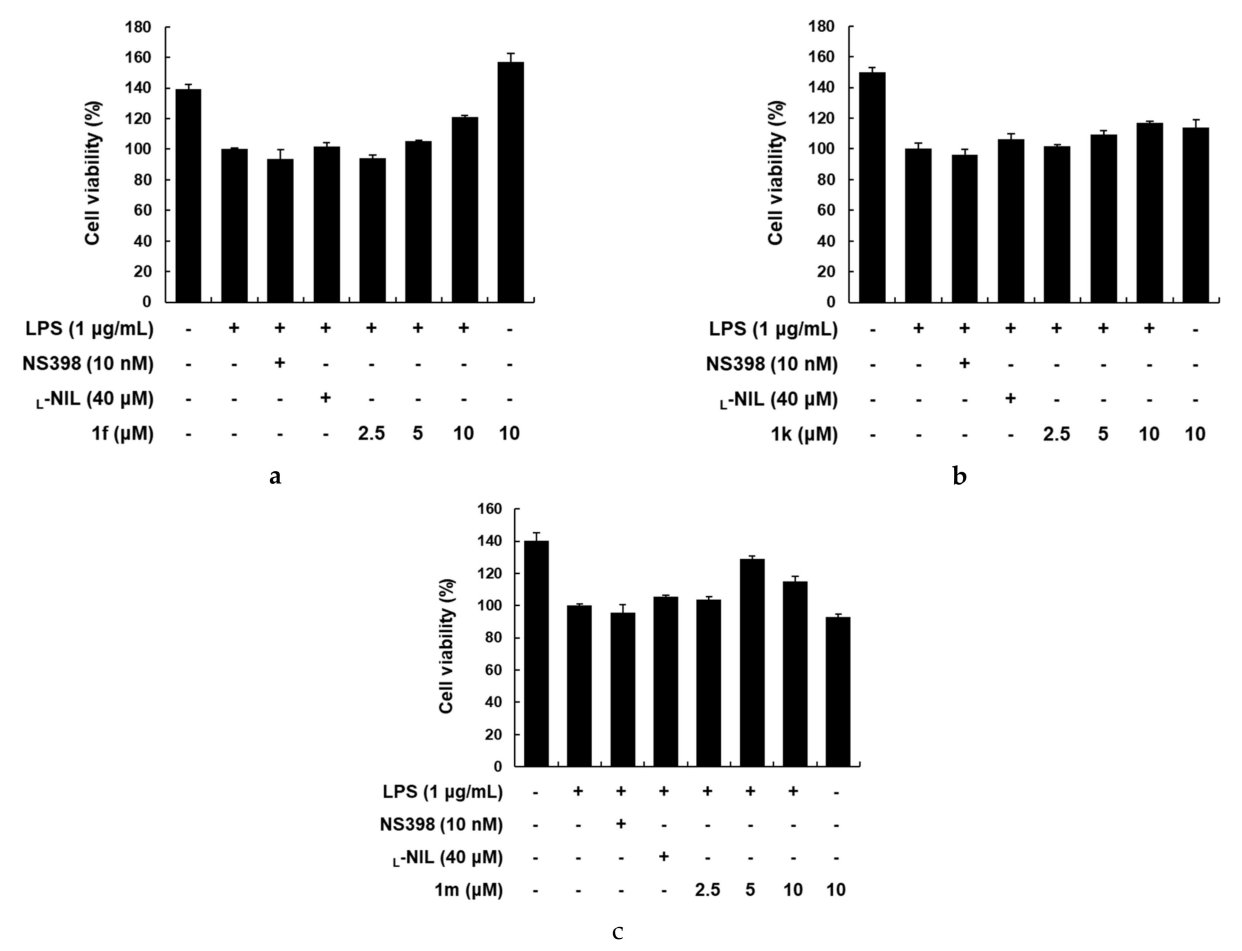
Effects of compounds **1f** (**a**), **1k** (**b**), **1m** (**c**), NS398, and _L_-NIL on LPS-induced RAW 264.7 cell viability at various concentrations (2.5, 5, or 10 μM).

Before screening the PGE_2_ and NO production inhibitory effects of the compounds, the compounds’ cytotoxicity was evaluated at 1 and 10 μM concentrations to make sure that the tested concentrations are safe enough and non-cytotoxic to avoid misleading results. All compounds were non-cytotoxic at 1 μM concentration, while by increasing concentration to 10 μM, all the target compounds except **1f**, **1k**, and **1m** started showing cytotoxicity. These three compounds were found to be non-cytotoxic at 10 μM concentration (Figure 2). The three compounds possess *N*-benzylpiperazinyl, *N*-phenylpiperazinyl, and *N*-(4-fluorobenzyl)piperazinyl moieties, respectively. The piperidinyl and the morpholino moieties seem to be unfavorable to avoid cytotoxicity in this series of compounds.

**Figure 3:**
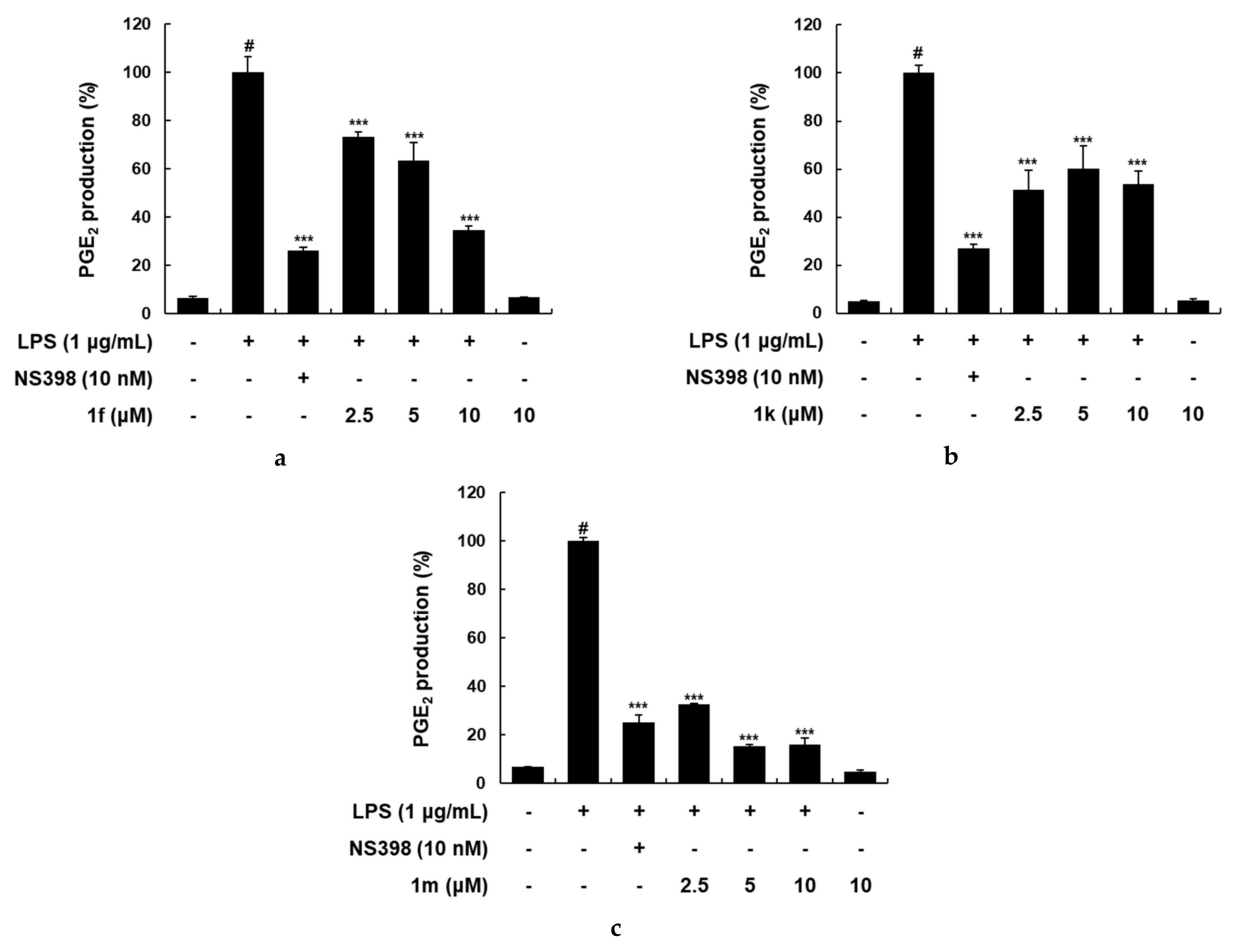
Effects of compounds **1f** (**a**), **1k** (**b**), and **1m** (**c**), and NS398 on LPS-stimulated PGE_2_ production in RAW 264.7 macrophages. # means significant difference from the negative control and *** means significant difference from the positive control.

Upon confirming the non-cytotoxicity of 10 μM concentration of these three derivatives against murine RAW 264.7 macrophages induced by LPS, compounds **1f**, **1k**, and **1m** were tested for inhibitory effect against LPS-induced PGE_2_ production together with checking their cell viability. They have shown no cytotoxicity at these levels (2.5, 5, 10 μM) and good inhibition values against PGE_2_ production (Figure 2 and Figure 3). Among these selected derivatives, compounds **1f** and **1m** showed dose-dependent inhibition along with increasing its concentration (37.4 at 5 μM to 65.4% at 10 μM) for compound **1f**, and 67% at 2.5 μM and 84.9% at 10 μM for compound **1m**. Furthermore, the IC_50_ values of compounds **1f** and **1m** were 7.6 and 1.1 μM, respectively. This indicates that compound **1m** with ethylene bridge was more active than compound **1f** possessing methylene bridge. The fluorine atom of compound **1m** might confer more lipophilicity that may result in more penetration inside the cell and hence better inhibition of PGE_2_ production. The fluorine atom can also add some more merits such as formation of an additional hydrogen bond with a hydrogen bond donor in the target protein and stronger hydrophobic interaction by fluorophenyl compared with unsubstituted phenyl. In addition, *p*-fluoro can prevent aromatic hydroxylation metabolic reaction and hence can elongate the duration of action [19]. Moreover, **1f** and **1m** were evaluated at 1 and 10 μM for inhibitory effects on LPS-induced NO production. It was found that compounds **1f** and **1m** showed inhibition values of 11.06% and 37.19%, respectively on LPS-induced NO production at 10 µM concentration (Table 2). Compound **1m** is slightly more active than L-NIL at 10 µM.

Compounds **1f**, **1k**, and **1m** were also tested for inhibitory effects against LPS-induced PGE_2_ production in addition to checking their cell viability. They have shown no cytotoxicity at these levels and good inhibition values against PGE_2_ production (Figure 2 and Figure 3). Among these selected derivatives, compounds **1f** and **1m** showed dose-dependent inhibition along with increasing its concentration (37.4% at 5 μM to 65.4% at 10 μM) for compound **1f**, and 67% at 2.5 μM and 84.9% at 10 μM for compound **1m**. Furthermore, the IC_50_ values of compounds **1f** and **1m** were 7.6 and 1.1 μM, respectively. This complies that compound **1m** with ethylene bridge was more active than compound **1f** possessing methylene bridge. The fluorine atom of compound **1m** might confer more lipophilicity that may result in more penetration inside the cell and hence better inhibition of PGE_2_ production.

**Figure 4:**
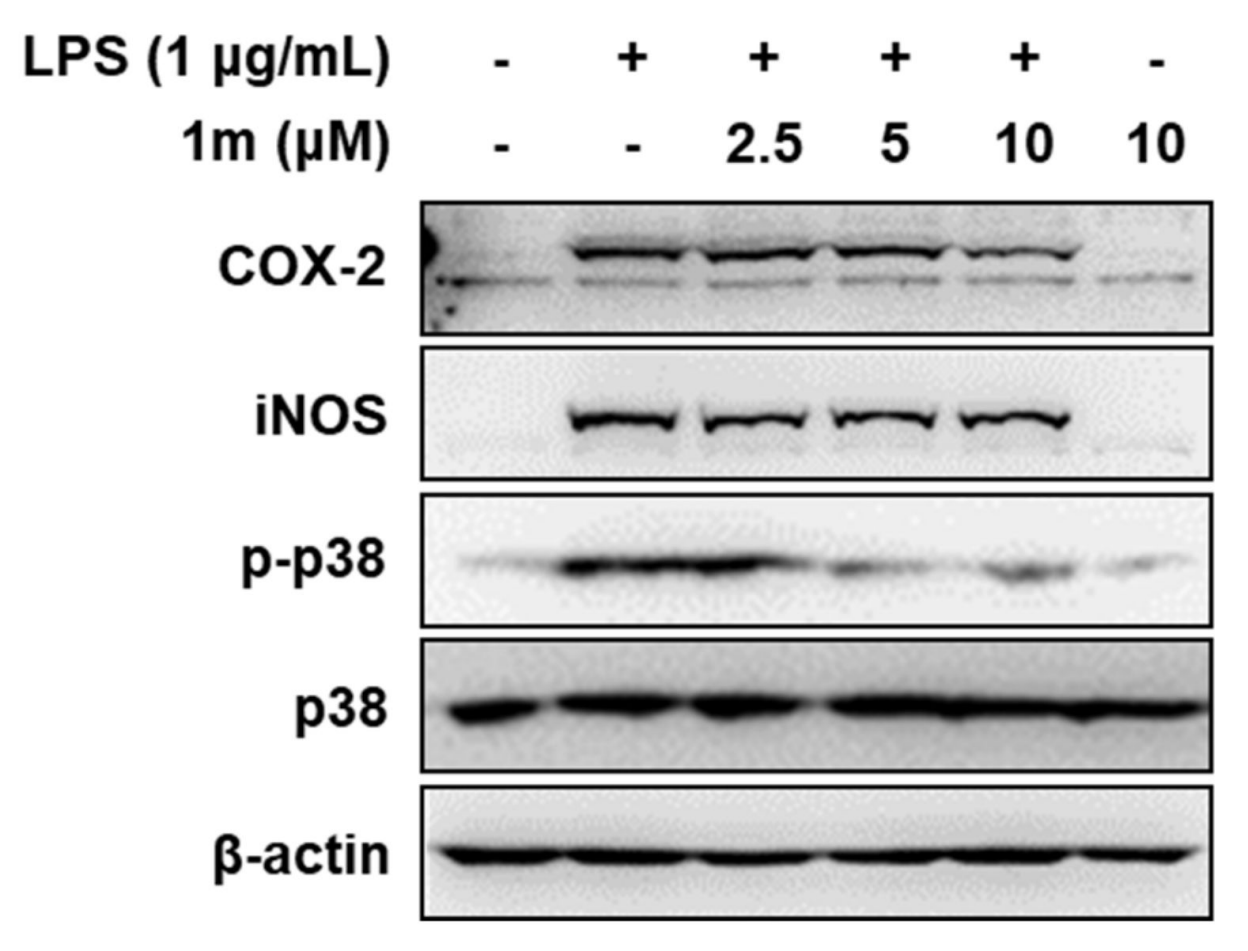
Effect of compound **1m** on COX-2, iNOS, and p-p38 protein expression in LPS-induced RAW 264.7 macrophages.

Furthermore, the most promising compound **1m** was chosen for a more extensive investigation of its molecular mechanism(s) of action. It was tested for inhibitory effects on COX-2 and iNOS protein expressions (Figure 4). Compound **1m** showed a concentration-dependent inhibitory effect against COX-2 and iNOS protein expression, especially at 10 µM concentration. Moreover, compound **1m** markedly suppressed the phosphorylation of p38, a key molecule in regulating inflammation [20].

## 3. Conclusions

Our target compounds were tested for potential cytotoxicity. We then selected the safest compounds for further investigations as PGE_2_ and NO production inhibitors in LPS-induced murine RAW 264.7 macrophages. The two tested compounds (**1f** and **1m**) act more against PGE_2_ production than over NO. We identified a couple of potential PGE_2_ production inhibitory compounds, namely **1f** and **1m**. The most potent compound, **1m**, exerted a strong inhibitory effect on PGE_2_ production with IC_50_ value of 1.1 μM and NO production with 37% at 10 μM. It produces these effects due to inhibition of both COX-2 and iNOS protein expression through inactivation of p38. Further structural optimization is needed in order to optimize activity.

## Figures and Tables

**Table 1:**
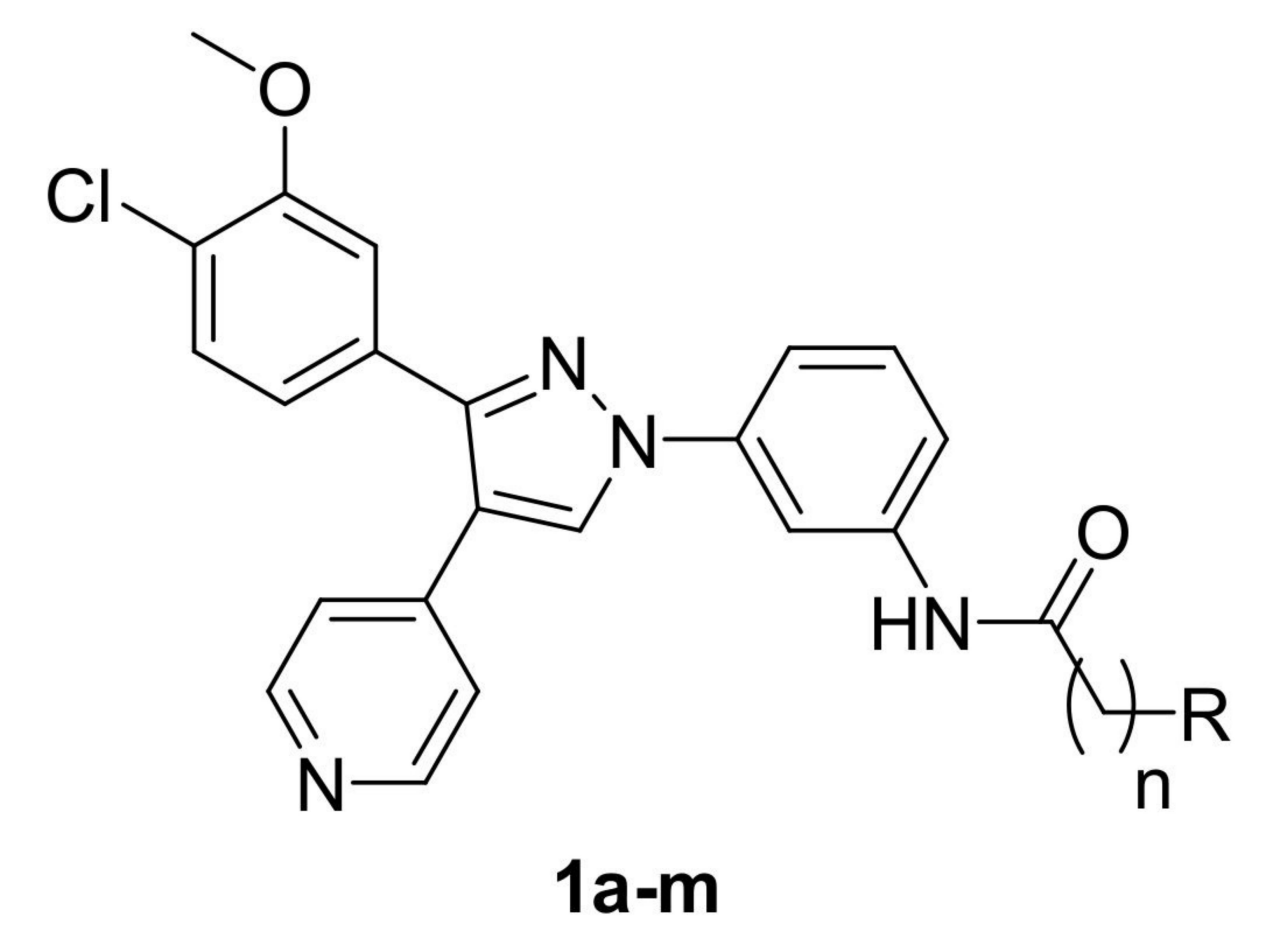
Structures of compounds **1a**–**m** and their cell viability results at 1 and 10 μM concentrations against murine RAW 264.7 macrophages.

Compound No.	n	R	Cell Viability (%)	
			1 μM ^a^	10 μM ^a^
**1a**	1	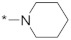	99 ± 5.9	10 ± 1.2
**1b**	1	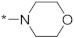	92 ± 4.5	65 ± 2.8
**1c**	1	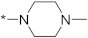	78 ± 3.3	5 ± 0.8
**1d**	1	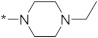	75 ± 2.9	3 ± 0.4
**1e**	1	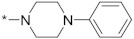	87 ± 4.1	65 ± 4.1
**1f**	1	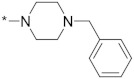	66 ± 1.8	**107** **± 8.5**
**1g**	2	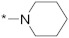	87 ± 3.8	5 ± 1.1
**1h**	2	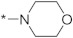	99 ± 2.8	75 ± 1.7
**1i**	2	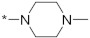	99 ± 3.4	5 ± 1.8
**1j**	2	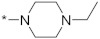	98 ± 3.1	5 ± 0.8
**1k**	2	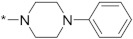	89 ± 2.8	**112** ± **7.6**
**1l**	2	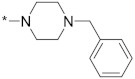	86 ± 3.7	34 ± 1.1
**1m**	2	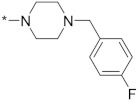	90 ± 4.1	**97** ± **3.9**

^a^ Data are presented as the means ± SDs of three independent experiments. Bold figures indicate non-cytotoxicity. * Indicates the site of connection to the main structure.

**Table 2:** Inhibitory effects of compounds **1f** and **1m** against NO production in LPS-induced RAW 264.7 cells.

Compound	Inhibition Rate (%)	
	1 (μM)	10 (μM)
**1f**	0	11.06 ± 1.5
**1m**	0	37.19 ± 3.4
**L-NIL**	3.9 ± 2.1	31.32 ± 2.9

## Data Availability

Not applicable.

## Supplementary Materials

The experimental procedures and the spectral analysis charts are available.

## Experimental

The synthetic procedures and the protocols for the biological assay are provided in the Supplementary File.

## Sample Availability

Samples of the compounds are available from the authors upon request.
