# Supplementary material for: Evaluation of the Inhibitory Effects of Pyridylpyrazole Derivatives on LPS-Induced PGE2 Productions and Nitric Oxide in Murine RAW 264.7 Macrophages
Source: Molecules. 2021 Oct 27;26(21):6489. doi: 10.3390/molecules26216489 (PMC8587869; doi:10.3390/molecules26216489)

Evaluation of the inhibitory effects of pyridylpyrazole derivatives on LPS-induced nitric oxide and PGE_2 productions in murine RAW 264.7 macrophages

Mahmoud M. Gamal El-Din[1] , Mohammed I. El-Gamal[2,3,4], Young-Do Kwon[5,6], Su-Yeon Kim[7,8], Hee-Soo Han[7,8] , Sang-Eun Park[7,8], Chang-Hyun Oh[9] , Kyung-Tae Lee[7,8*] and Hee-Kwon Kim[5,6,*].

[1] Pharmaceutical and Drug Industries Research Division, National Research Centre, Dokki-Giza, 12622, Egypt

[2] Department of Medicinal Chemistry, College of Pharmacy, University of Sharjah, Sharjah 27272, United Arab Emirates

[3] Sharjah Institute for Medical Research, University of Sharjah, Sharjah 27272, United Arab Emirates

[4] Department of Medicinal Chemistry, Faculty of Pharmacy, Mansoura University, Mansoura 35516, Egypt

[5] Department of Nuclear Medicine, Molecular Imaging & Therapeutic Medicine Research Center, Jeonbuk National University Medical School and Hospital, 20 Geonji-ro, Deokjin-gu, Jeonju 54907, Republic of Korea

[6] Research Institute of Clinical Medicine of Jeonbuk National University-Biomedical Research Institute of Jeonbuk National University Hospital, 20 Geonji-ro, Deokjin-gu, Jeonju 54907, Republic of Korea

[7] Department of Pharmaceutical Biochemistry, Kyung Hee University, Seoul 02447, Republic of Korea

[8] Department of Life and Nanopharmaceutical Sciences, Graduate School, Kyung Hee University, 26, Kyungheedae-ro, Seoul 02447, Republic of Korea

[9] Center for Biomaterials, Korea Institute of Science and Technology, PO Box 131, Cheongryang, Seoul 130-650, Republic of Korea

* Corresponding author: Tel.: +82 63 250 2768; fax: +82 63 255 1172; E-mail address: hkkim717@jbnu.ac.kr .
Address: Department of Nuclear Medicine, Molecular Imaging & Therapeutic Medicine Research Center, Jeonbuk National University Medical School and Hospital, 20 Geonji-ro, Deokjin-gu, Jeonju 54907, Republic of Korea.
* Corresponding author: Tel.: +82 2 961 0860; fax: +82 2 961 9580; E-mail address ktlee@khu.ac.kr
. Address: Department of Pharmaceutical Biochemistry, Kyung Hee University, Seoul 02447, Republic of Korea.

Supplementary data

Synthetic procedures 3-8

Biological evaluation procedures …………………………………………… 9

Representative ^{1}H NMR, ^{13}C NMR, and MS charts ………………………..… 10-55

Synthetic procedures

*2-Chloro-N-(3-(3-(4-chloro-3-methoxyphenyl)-4-(pyridin-4-yl)-1H-pyrazol-1-yl)phenyl)-acetamide (**9a**)* or *3-chloro-N-(3-(3-(4-chloro-3-methoxyphenyl)-4-(pyridin-4-yl)-1H-pyrazol-1-yl)phenyl)propanamide (**9b**)*

To a cooled solution of compound **8** (0.5 g, 1.325 mmol) in anhydrous methylene chloride (15 mL) at -10 ℃, triethylamine (148 mg, 1.4575 mmol) was added dropwise within 5 minutes then either chloroacetyl chloride or chloropropionyl chloride were added dropwise keeping the reaction mixture temperature at -10 ℃ for another 5 minutes. To the reaction mixture, water (15 mL) was added, then reaction mixture was swirled then the organic layer was separated. The water layer was further extracted with methylene chloride once again. The combined organic layers were combined, washed with brine and dried using anhydrous Na_2SO_4. Then evaporated under reduced pressure to give the target compounds that were used in the next step without further purification with yield 65%.

*N-(3-(3-(4-Chloro-3-methoxyphenyl)-4-(pyridin-4-yl)-1H-pyrazol-1-yl)phenyl)-2-(piperidin-1-yl)acetamide (**1a**)*

To a solution of **9a** (100 mg, 0.175 mmol) in anhydrous methylene chloride (5 mL), triethylamine (20 mg, 0.193 mmol) was added at 0 ℃, stirred for 15 min, then morpholine (18.3 mg, 0.21 mmol) was added dropwise. The reaction mixture was stirred for 24 hours at r.t. Upon completion of the reaction, the mixture was dried and then was partitioned between ethyl acetate and aqueous phase. Followed by separation of the organic layer and extraction of the aqueous layer with organic solvent (3x10 mL) was repeated. The collected organic layers were washed with NaCl solution (5%) and the organic solvent was evaporated under vacuum. The residue was purified using column chromatography (silica gel, hexane:ethyl acetate 4:1 v/v) to give the required product as a yellow oil. Yield: 55%; ^{1}H NMR (400 MHz, $CDCl_3$) δ 8.48 (d, 2H, *J* = 4.8 Hz, pyridyl protons), 8.14 (s, 1H, pyrazolyl-H), 7.45 (d, 2H, *J* = 4.8 Hz, pyridyl protons), 7.39 (d, 1H, *J* = 8.0 Hz, Ph), 7.38 (t, 1H, *J* = 8.0 Hz, Ph), 7.28 (d, 1H, *J* = 8.0 Hz, Ph), 7.19-7.18 (m, 2H, Ph), 7.07 (brs, 1H, NH), 7.01 (dd, 1H, *J* = 2.0, 8.0 Hz, Ph), 3.74 (s, 3H, OCH_3), 3.22 (s, 2H, $COCH_2N$), 2.65 (brs, 4H, piperidinyl protons, 2',6'), 1.67 (t, 4H, *J* = 5.2 Hz, piperidinyl protons,

3’,5’), 1.47 (brs, 2H, piperidinyl protons, 4’); ^{13}C NMR (100 MHz, $CDCl_3$) δ 170.6 (C=O), 154.9, 150.0, 139.6, 139.3, 138.9, 132.1, 130.3, 130.1, 127.6, 122.9, 122.8, 121.5, 120.3, 117.9, 114.9, 112.3, 110.2 [Ar-C], 62.2 (CO$\underline{C}H_2$N), 56.3 (OCH_3), 55.2 (piperidinyl carbons, 2’,6’), 25.7 (piperidinyl carbons, 3’,5’), 23.2 (piperidinyl carbon, 4’); MS m/z: 502.43 $[M+H]^+$.

Compounds **1b-g** were synthesized from **9a** and appropriate amine derivatives as reported in the synthesis of **1a**.

*N-(3-(3-(4-Chloro-3-methoxyphenyl)-4-(pyridin-4-yl)-1H-pyrazol-1-yl)phenyl)-2-morpholino-acetamide (**1b**)*

Yellow oil; yield: 46%; ^{1}H NMR (400 MHz, $CDCl_3$) δ 9.33 (brs, 1H, NH), 8.56 (d, 2H, *J* = 4.8 Hz, pyridyl protons), 8.03 (s, 1H, pyrazolyl-H), 7.57 (d, 1H, *J* = 7.6 Hz, Ph), 7.51-7.45 (m, 2H, pyridyl protons), 7.36 (d, 1H, *J* = 8.4 Hz, Ph), 7.29-7.27 (m, 2H, Ph), 7.14 (s, 1H, Ph), 7.09 (d, 1H, *J* = 8.0 Hz, Ph), 3.80 (m, 7H, OCH_3 and morpholino-4H), 3.23 (s, 2H, $COCH_2N$), 2.69-2.67 (m, 4H, morpholino-4H); ^{13}C NMR (100 MHz, $CDCl_3$) δ 168.1 (C=O), 154.6, 150.1, 149.7, 140.8, 139.9, 138.7, 132.2, 130.5, 130.2, 127.6, 122.9, 122.8, 121.2, 117.9, 114.9, 112.3, 110.2 [Ar-C], 66.9 (morpholino $\underline{C}H_2O\underline{C}H_2$), 62.4 (CO$\underline{C}H_2$N), 56.1 ($OCH_3$), 50.8 (morpholino $\underline{C}H_2N\underline{C}H_2$); MS m/z: 504.1 $[M+H]^+$.

*N-(3-(3-(4-Chloro-3-methoxyphenyl)-4-(pyridin-4-yl)-1H-pyrazol-1-yl)phenyl)-2-(4-methyl-piperazin-1-yl)acetamide (**1c**)*

Yellow oil; yield: 48%; ^{1}H NMR (400 MHz, $CDCl_3$) δ 9.28 (brs, 1H, NH), 8.58 (s, 2H, pyridyl protons), 8.13-8.10 (m, 2H, pyrazolyl-H and Ph), 7.59-7.56 (m, 2H, pyridyl protons), 7.46 (t, 1H, *J* = 8.0 Hz, Ph), 7.38 (d, 1H, *J* = 8.0 Hz, Ph), 7.29-7.27 (m, 2H, Ph), 7.15-7.08 (m, 2H, Ph), 3.83 (s, 3H, OCH_3), 3.31 (s, 2H, $COCH_2N$), 2.94-2.60 (m, 8H, piperazinyl protons), 1.28 (s, 3H, N-CH_3); ^{13}C NMR (100 MHz, $CDCl_3$) δ 170.0 (C=O), 154.9, 149.9, 149.7, 140.6, 139.9, 139.8, 132.3, 130.2, 130.0, 127.6, 122.8, 122.6, 121.4, 120.0, 117.5, 114.2, 112.3, 110.2 [Ar-C], 62.7 (CO$\underline{C}H_2$N), 56.0 (OCH_3), 55.1 (piperazinyl carbons), 45.8 (N-CH_3); MS m/z: 517.42 $[M+H]^+$.

*N-(3-(3-(4-Chloro-3-methoxyphenyl)-4-(pyridin-4-yl)-1H-pyrazol-1-yl)phenyl)-2-(4-ethyl-piperazin-1-yl)acetamide (**1d**)*

Yellow oil; yield: 49%; ^{1}H NMR (400 MHz, $CDCl_3$) δ 9.29 (brs, 1H, NH), 8.58 (brs, 2H, pyridyl protons), 8.22-8.15 (m, 2H, pyrazolyl-H and Ph), 7.59-7.55 (m, 2H, pyridyl protons), 7.48 (t, 1H, J = 8.0 Hz, Ph), 7.38 (d, 1H, J = 8.0 Hz, Ph), 7.16-7.08 (m, 4H, Ph), 3.83 (m, 3H, OCH_3), 3.27 (s, 2H, $COCH_2N$), 2.83-2.50 (m, 10H, piperazinyl protons and C$\underline{H_2}$$CH_3$), 1.25 (t, 3H, J = 7.6 Hz, $CH_2C\underline{H_3}$); ^{13}C NMR (100 MHz, $CDCl_3$) δ 169.0 (C=O), 155.0, 150.1, 149.9, 139.9, 139.3, 138.9, 132.1, 130.3, 130.0, 127.6, 127.5, 122.5, 122.3, 122.1, 121.5, 121.4, 120.3, 117.9, 114.8,114.7 112.3, 110.2 [Ar-C], 61.7 ($CO\underline{C}H_2N$), 56.1 (OCH_3), 52.4 (piperazinyl carbons), 52.2 (piperazinyl carbons and $\underline{C}H_2CH_3$), 29.7 ($CH_2\underline{C}H_3$); MS m/z: 531.0 $[M+H]^+$.

*N-(3-(3-(4-Chloro-3-methoxyphenyl)-4-(pyridin-4-yl)-1H-pyrazol-1-yl)phenyl)-2-(4-phenyl-piperazin-1-yl)acetamide (**1e**)*

Yellow oil; yield: 60%; ^{1}H NMR (400 MHz, $CDCl_3$) δ 8.48 (d, 1H, J = 4.8 Hz, pyridyl protons), 8.13 (s, 1H, pyrazolyl-H), 7.63 (brs, 1H, NH), 7.51-7.32 (m, 3H, pyridyl protons and Ph), 7.32-7.18 (m, 6H, Ph), 7.06 (s, 1H, Ph), 6.98 (d,1H, J = 8.0 Hz, Ph), 6.88-6.83 (m, 3H, Ph), 3.71 (s, 3H, OCH_3), 3.25-3.24 (m, 4H, piperazinyl protons), 2.81 (s, 2H, $COCH_2N$), 2.45-2.30 (m, 4H, piperazinyl protons); MS m/z: 578.9 $[M+H]^+$.

*2-(4-Benzylpiperazin-1-yl)-N-(3-(3-(4-chloro-3-methoxyphenyl)-4-(pyridin-4-yl)-1H-pyrazol-1-yl)phenyl)acetamide (**1f**)*

Yellow oil; yield: 65%; ^{1}H NMR (400 MHz, $CDCl_3$) δ 8.57 (d, 2H, J = 5.6 Hz, pyridyl protons), 8.38 (brs, 1H, NH), 8.18 (s, 1H, pyrazolyl-H), 7.78-7.75 (m, 3H, Ph), 7.59-7.53 (m, 3H, Ph), 7.45-7.32 (m, 6H, Ph), 7.14-7.07 (m, 2H, Ph), 3.84 (s, 3H, OCH_3), 3.31 (s, 2H, benzyl-C$\underline{H_2}$), 3.10 (brs, 4H, piperazinyl protons and $COCH_2N$), 2.36 (brs, 4H, piperazinyl protons), 2.32 (brs, 2H, piperazinyl protons); MS m/z: 592.9 $[M+H]^+$.

Compounds **1g-m** were prepared from **9b** and appropriate acid chloride as reported in the synthesis of **1a**

*N-(3-(3-(4-Chloro-3-methoxyphenyl)-4-(pyridin-4-yl)-1H-pyrazol-1-yl)phenyl)-3-(piperidin-1-yl)propanamide (**1g**)*

Yellow oil; yield: 62%; ^{1}H NMR (400 MHz, $CDCl_3$) δ 8.59 (brs, 1H, NH), 8.36 (s, 1H, Ph), 8.19 (s, 1H, pyrazolyl-H), 7.57 (d, 1H, *J* = 8.0 Hz, pyridyl protons), 7.44 (t, 1H, *J* = 8.0 Hz, Ph), 7.36 (d, 2H, *J* = 8.0 Hz, pyridyl protons), 7.32-7.29 (m, 3H, Ph), 7.17 (s, 1H, Ph), 7.10 (d, 1H, *J* = 7.6 Hz, Ph), 3.83 (s, 3H, OCH_3), 2.84-2.67 (m, 6H, N-CH_2 and piperidinyl protons), 1.79-1.28 (m, 8H, $COCH_2$ and piperidinyl protons); ^{13}C NMR (100 MHz, $CDCl_3$) δ 170.7 (C=O), 154.9, 150.1, 149.9, 149.8, 140.1, 139.9, 132.6, 130.5, 130.2, 129.6, 127.6, 122.7, 120.5, 120.2, 117.7, 114.9, 112.2, 110.2 [Ar-C], 56.1 (OCH_3), 54.1 (piperidinyl $\underline{C}H_2N\underline{C}H_2$), 53.6 ($\underline{C}H_2CH_2CO$), 32.6 ($CH_2\underline{C}H_2CO$), 25.7 (piperidinyl 3’, 4’,5’-carbons); MS m/z: 516.21 $[M+H]^+$.

*N-(3-(3-(4-Chloro-3-methoxyphenyl)-4-(pyridin-4-yl)-1H-pyrazol-1-yl)phenyl)-3-morpholino-propanamide (**1h**)*

Yellow oil; yield: 58%; ^{1}H NMR (400 MHz, $CDCl_3$) δ 9.33 (brs, 1H, NH), 8.56 (d, 2H, *J* = 4.8 Hz, pyridyl protons), 8.03 (s, 1H, pyrazolyl-H), 7.57 (d, 1H, *J* = 7.6 Hz, pyridyl protons), 7.51-7.45 (m, 2H, Ph), 7.46 (d, 1H, *J* = 8.4 Hz, Ph), 7.29-7.27 (m, 2H, Ph), 7.16 (s, 1H, Ph), 7.07 (d, 1H, *J* = 8.0 Hz, Ph), 3.83-3.75 (m, 9H, OCH_3, morpholino protons, and $C\underline{H_2}CH_2CO$), 3.23 (s, 2H, $CH_2C\underline{H_2}CO$), 2.69-2.67 (m, 4H, morpholino protons); ^{13}C NMR (100 MHz, $CDCl_3$) δ 168.1 (C=O), 154.6, 150.1, 149.7, 140.8, 139.9, 138.7, 132.2, 130.5, 130.2, 127.6, 122.9, 122.8, 122.2, 117.9, 114.9, 112.3, 110.2 [Ar-C], 66.9 (morpholino $\underline{C}H_2O\underline{C}H_2$), 62.4 (morpholino $\underline{C}H_2N\underline{C}H_2$), 56.1 ($OCH_3$), 50.8 ($\underline{C}H_2CH_2CO$), 25.2 ($CH_2\underline{C}H_2CO$); MS m/z: 518.43 $[M+H]^+$.

*N-(3-(3-(4-Chloro-3-methoxyphenyl)-4-(pyridin-4-yl)-1H-pyrazol-1-yl)phenyl)-3-(4-methyl-piperazin-1-yl)propanamide (**1i**)*

Yellow oil; yield: 56%; ^{1}H NMR (400 MHz, $CDCl_3$) δ 8.49 (d, 2H, *J* = 6.4 Hz, pyridyl protons), 8.29 (s, 1H, Ph), 8.15 (s, 1H, pyrazolyl-H), 7.49 (dd, 1H, *J* = 1.2, 8.0 Hz, pyridyl protons), 7.36 (t, 1H, *J* = 8.0 Hz, Ph), 7.28-7.20 (m, 4H, Ph), 7.08 (s, 1H, Ph), 7.03 (dd, 1H, *J* = 1.6, 8.0 Hz, Ph), 3.73 (s, 3H, OCH_3), 2.73-2.51 (m, 12H, piperazinyl protons and $C\underline{H_2}C\underline{H_2}CO$), 2.31 (s, 3H,

N-CH_3); ^{13}C NMR (100 MHz, $CDCl_3$) δ 170.7 (C=O), 154.6, 149.9, 149.6, 140.6, 139.9, 139.8, 132.3, 130.1, 129.6, 127.6, 122.9, 122.6, 121.4, 120.1, 117.6, 114.1, 112.3, 110.2 [Ar-C], 56.0 (OCH_3), 55.1 (piperazinyl carbons), 53.4 (piperazinyl carbons), 53.1 (piperazinyl carbons and $\underline{C}H_2CH_2CO$), 45.8 (N-CH_3), 32.5 ($CH_2\underline{C}H_2CO$); MS m/z: 529.9 $[M+H]^+$.

*N-(3-(3-(4-Chloro-3-methoxyphenyl)-4-(pyridin-4-yl)-1H-pyrazol-1-yl)phenyl)-3-(4-ethyl-piperazin-1-yl)propanamide (**1j**)*

Yellow oil; yield: 61%; ^{1}H NMR (400 MHz, $CDCl_3$) δ 8.56 (d, 2H, J = 6.4 Hz, pyridyl protons), 8.32 (s, 1H, NH), 8.19 (s, 1H, pyrazolyl-H), 7.56 (dd, 1H, J = 1.2, 8.0 Hz, Ph), 7.42 (t, 1H, J = 8.0 Hz, Ph), 7.39-7.35 (m, 2H, Ph), 7.28 (d, 2H, J = 7.2 Hz, pyridyl protons), 7.14-7.09 (m, 2H, Ph), 3.73 (s, 3H, OCH_3), 2.73-2.51 (m, 15H, piperazinyl proton, $C\underline{H_2}CH_2CO$, and ethyl protons), 2.31 (brs, 2H, $COCH_2$); ^{13}C NMR (100 MHz, $CDCl_3$) δ 170.7 (C=O), 154.6, 149.9, 149.6, 140.6, 139.9, 139.8, 132.3, 130.1, 129.6, 127.6, 122.9, 122.6, 121.4, 120.1, 117.6, 114.1, 112.3, 110.2 [Ar-C], 56.1 (OCH_3), 55.5 (piperazinyl carbons), 52.5 (piperazinyl carbons), 52.3 ($\underline{C}H_2CH_2CO$), 52.1 ($\underline{C}H_2CH_3$), 32.3 ($CH_2\underline{C}H_2CO$), 11.9 ($CH_2\underline{C}H_3$); MS m/z: 544.9 $[M+H]^+$.

*N-(3-(3-(4-Chloro-3-methoxyphenyl)-4-(pyridin-4-yl)-1H-pyrazol-1-yl)phenyl)-3-(4-phenyl-piperazin-1-yl)propanamide (**1k**)*

Yellow oil; yield: 67%; ^{1}H NMR (400 MHz, $CDCl_3$) δ 8.58 (d, 2H, J = 6.0 Hz, pyridyl protons), 8.34 (s, 1H, NH), 8.21 (s, 1H, pyrazolyl-H), 7.58 (d, 1H, J = 7.2 Hz, Ph), 7.43 (t, 1H, J = 8.0 Hz, Ph), 7.35-7.28 (m, 5H, pyridyl protons and Ph), 7.15-7.08 (m, 2H, Ph), 6.96-6.93 (m, 3H, Ph), 3.79 (s, 3H, OCH_3), 2.96-2.65 (m, 12H, piperazinyl protons and CH_2CH_2CO); ^{13}C NMR (100 MHz, $CDCl_3$) δ 169.7 (C=O), 155.1, 151.9, 150.9, 150.8, 146.9, 142.9, 140.0, 132.0, 130.3, 129.3, 127.5, 123.1, 122.4, 121.5, 120.5, 120.3, 117.6, 117.2, 116.4, 114.5, 112.2, 110.2 [Ar-C], 56.1 (OCH_3), 53.1 (piperazinyl carbons), 52.4 (piperazinyl carbons and $\underline{C}H_2CH_2CO$), 32.5 ($CH_2\underline{C}H_2CO$); MS m/z: 593.43 $[M+H]^+$.

*3-(4-Benzylpiperazin-1-yl)-N-(3-(3-(4-chloro-3-methoxyphenyl)-4-(pyridin-4-yl)-1H-pyrazol-1-yl)phenyl)propanamide (**1l**)*

Yellow oil; yield: 71%; ^{1}H NMR (400 MHz, $CDCl_3$) δ 8.57 (d, 2H, *J* = 6.0 Hz, pyridyl protons), 8.36 (s, 1H, NH), 8.21 (s, 1H, pyrazolyl-H), 7.58 (dd, 1H, *J* = 1.2, 9.2 Hz, Ph), 7.43 (t, 1H, *J* = 8.0 Hz, Ph), 7.38-7.29 (m, 9H, pyridyl protons and Ph), 7.16-7.11 (m, 2H, Ph), 3.79 (s, 3H, OCH_3), 3.58 (s, 2H, benzyl-C$\underline{H}_2$), 2.81-2.58 (m, 12H, piperazinyl protons and CH_2CH_2CO); ^{13}C NMR (100 MHz, $CDCl_3$) δ 170.9 (C=O), 156.9, 150.9, 150.8, 146.9, 142.9, 140.0, 137.6, 132.5, 130.3, 129.1, 127.6, 123.0, 121.5, 120.2, 117.6, 114.3, 112.3, 110.2 [Ar-C], 62.9 (benzyl-$\underline{C}H_2$), 56.1 (OCH_3), 53.1 (piperazinyl carbons), 52.3 (piperazinyl carbons and $\underline{C}H_2CH_2CO$), 32.6 ($CH_2\underline{C}H_2CO$); MS m/z: 608.1 $[M+H]^+$.

*3-(4-(4-Fluorobenzyl)piperazin-1-yl)-N-(3-(3-(4-chloro-3-methoxyphenyl)-4-(pyridin-4-yl)-1H-pyrazol-1-yl)phenyl)propanamide (**1m**)*

Yellow oil; yield: 68%; ^{1}H NMR (400 MHz, $CDCl_3$) δ 8.49 (d, 2H, *J* = 6.0 Hz, pyridyl protons), 8.26 (s, 1H, NH), 8.09 (s, 1H, pyrazolyl-H), 7.48 (d, 1H, *J* = 8.0 Hz, Ph), 7.36 (t, 1H, *J* = 8.0 Hz, Ph), 7.26 (d, 1H, *J* = 8.0 Hz, Ph)), 7.19-7.17 (m, 4H, , pyridyl protons and Ph), 7.08-7.01 (m, 2H, Ph), 6.92 (t, 3H, *J* = 8.8 Hz, Ph), 3.71 (s, 3H, OCH_3), 3.44 (s, 2H, benzyl-C$\underline{H}_2$), 2.74-2.51 (m, 12H, piperazinyl protons and CH_2CH_2CO); ^{13}C NMR (100 MHz, $CDCl_3$) δ 170.7 (C=O), 154.9, 149.9 (d, *J* = 17.0 Hz, C-F coupling), 140.6, 140.0, 133.2, 132.2, 130.6, 130.3, 127.1, 122.9 (d, *J* = 30.0 Hz, C-F coupling), 121.5, 120.3, 117.5, 115.2 (d, *J* = 21.0 Hz, C-F coupling), 114.3, 112.3, 110.2 [Ar-C], 61.9 (benzyl-$\underline{C}H_2$), 56.1 (OCH_3), 53.4 (piperazinyl carbons), 53.2 (piperazinyl carbons and $\underline{C}H_2CH_2CO$), 32.5 ($CH_2\underline{C}H_2CO$); MS m/z: 625.0 $[M+H]^+$.

Biological evaluation

Cell culture and sample treatment, nitrite determination, PGE_2 assay, and MTT assay for cell viability have been carried out following the reported procedures [*Bioorg. Med. Chem. Lett.* **2014**, *24*, 571 & *Eur. J. Pharmacol.* **2008**, *584*, 175].

Western blot

RAW 264.7 macrophage cells were re-suspended in PRO-PREP^{TM} protein extraction solution (Intron Biotechnology, Seoul, Korea) and incubated with 20 min at 4 °C. The cell debris was removed by microcentrifugation, and the supernatants were quickly frozen. The protein concentration was determined using the Bio-Rad protein assay reagent according to the manufacture's instruction. Cellular proteins were electroblotted onto a PVDF membrane following separation on 8-12% SDS-polyacrylamide gel electrophoresis. The immunoblot was incubated with blocking solution (5% skimmed milk) at room temperature for 1 h, followed by incubation overnight with a primary antibody at 4 °C. The blots were washed four times with a Tween 20/Tris-buffered saline (T/TBS) and incubated with a 1:2000 dilution of horseradish peroxidase-conjugated secondary antibody for 2 h at room temperature. The blots were again washed three times with T/TBS, and then developed by enhanced chemiluminescence (GE healthcare, WI, USA).

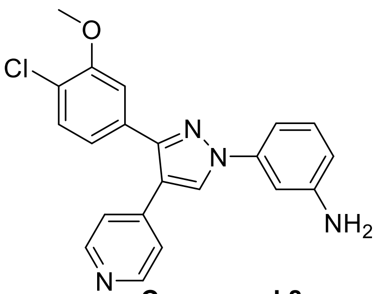
O
Cl
N
N
NH2
N

Compound 8

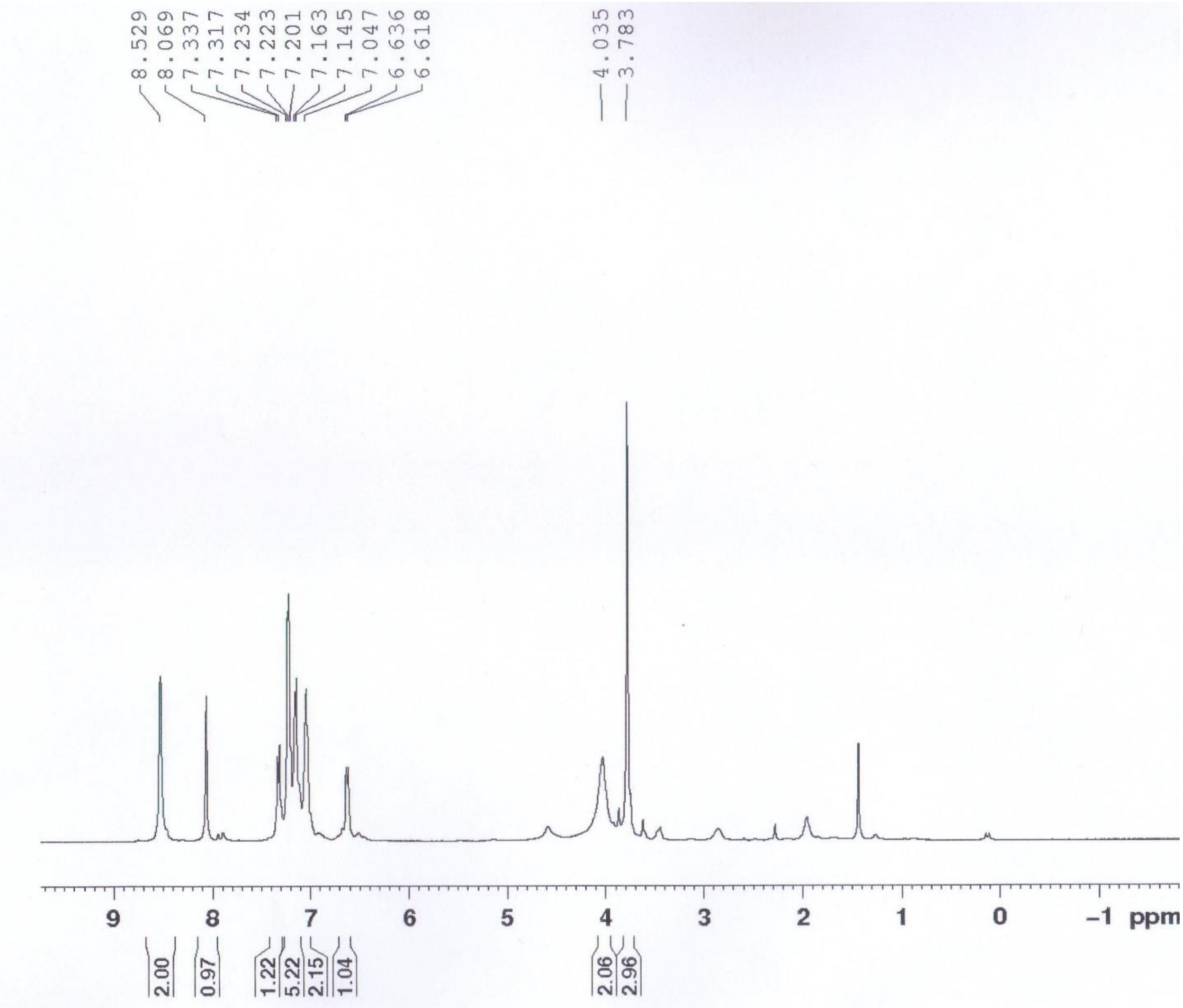
8.529
8.069
7.337
7.317
7.234
7.223
7.201
7.163
7.145
7.047
6.636
6.618
4.035
3.783
9
8
7
6
5
4
3
2
1
0
–1
ppm
2.00
0.97
1.22
5.22
2.15
1.04
2.06
2.96

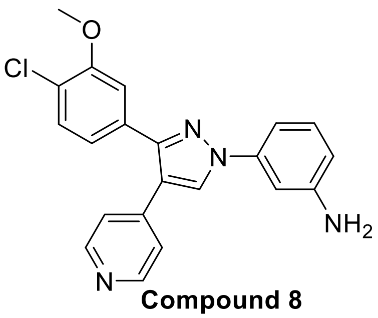

Compound 8

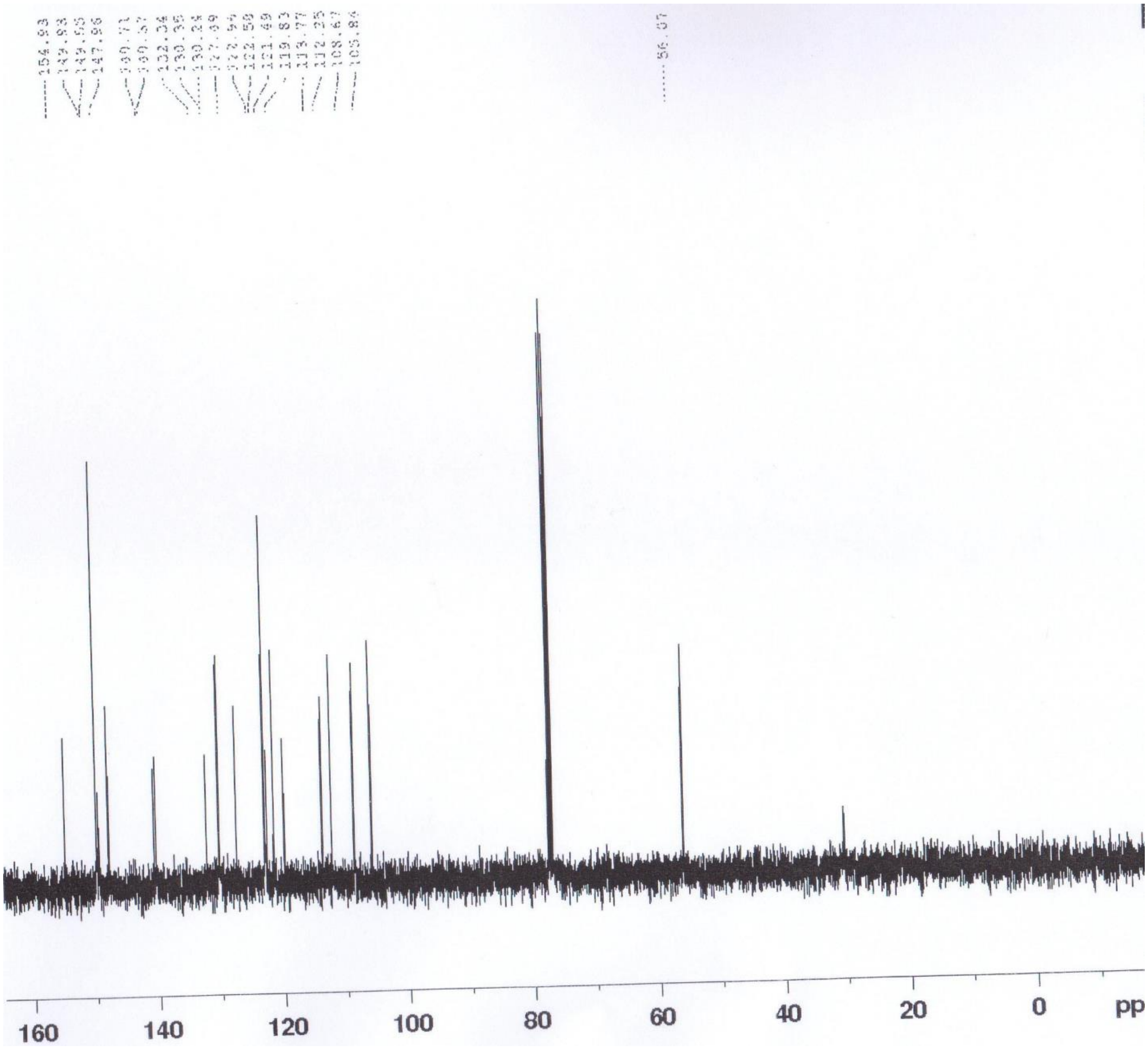

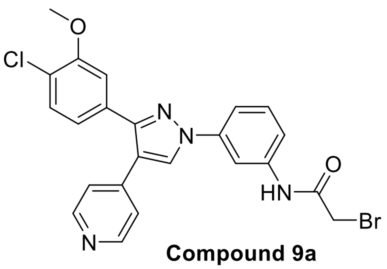

Cl
O
N
N
HN
O
Br
N

Compound 9a

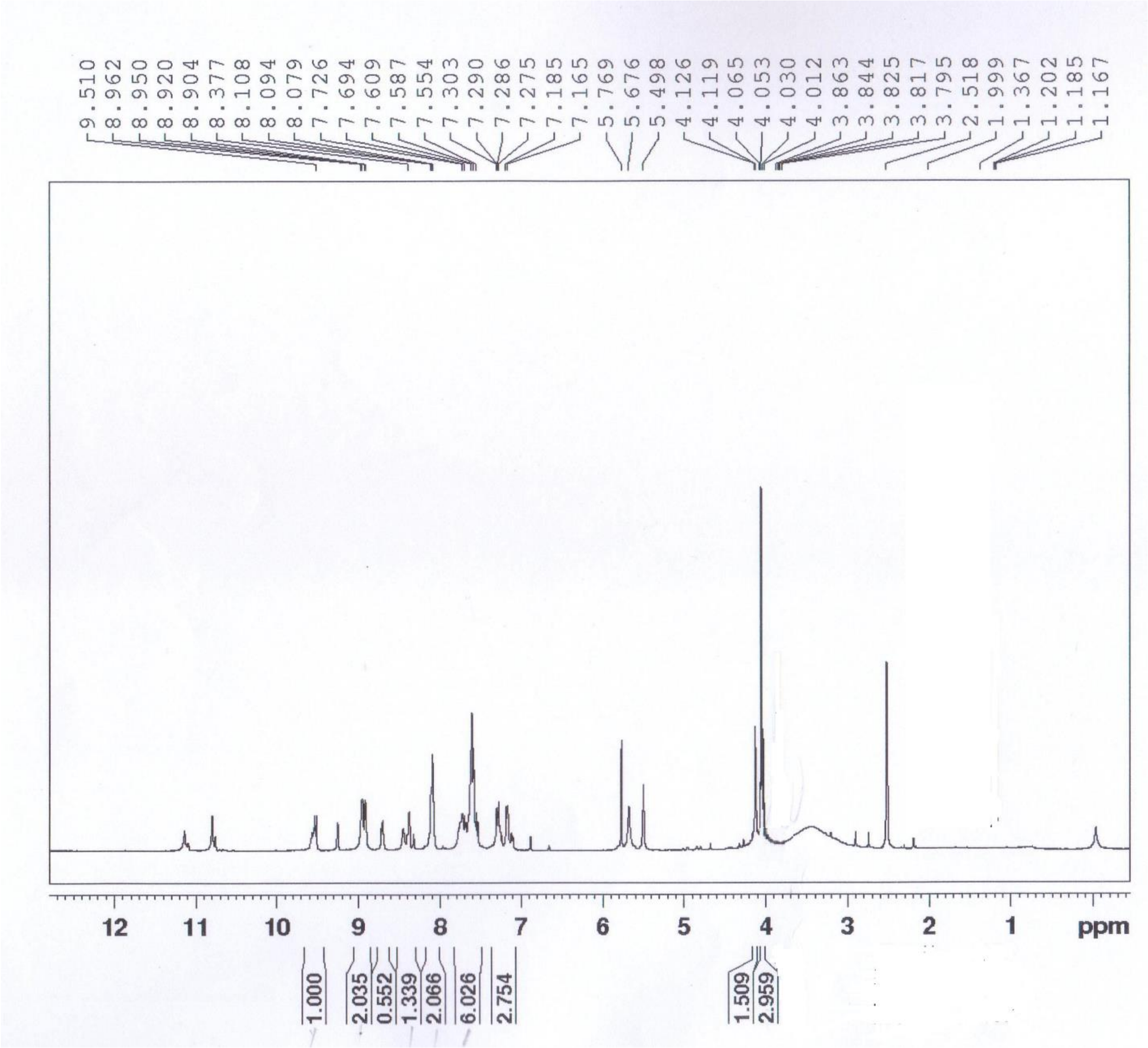

9.510
8.962
8.950
8.920
8.904
8.377
8.108
8.094
8.079
7.726
7.694
7.609
7.587
7.554
7.303
7.290
7.286
7.275
7.185
7.165
5.769
5.676
5.498
4.126
4.119
4.065
4.053
4.030
4.012
3.863
3.844
3.825
3.817
3.795
2.518
1.999
1.367
1.202
1.185
1.167
12 11 10 9 8 7 6 5 4 3 2 1 ppm
1.000
2.035
0.552
1.339
2.066
6.026
2.754
1.509
2.959

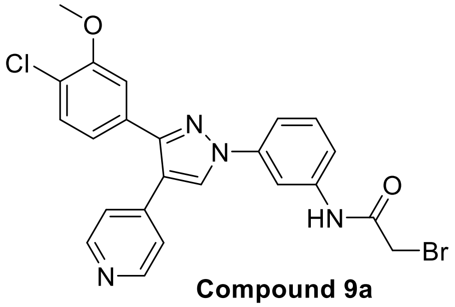
Cl
O
N
N
N
HN
O
Br

Compound 9a

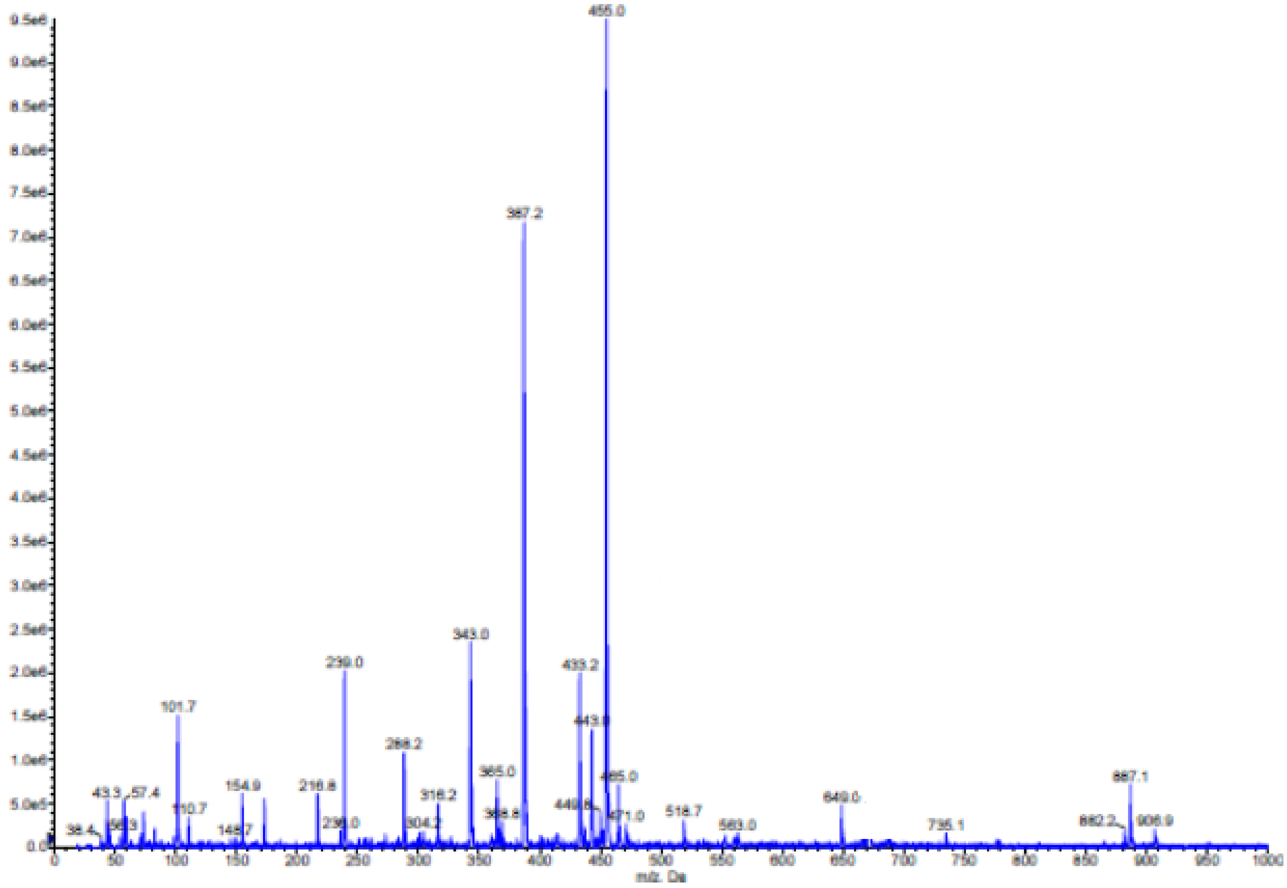
455.0
387.2
343.0
239.0
433.2
101.7
288.2
365.0
485.0
887.1
649.0
518.7
735.1
906.9
882.2
m/z, Da

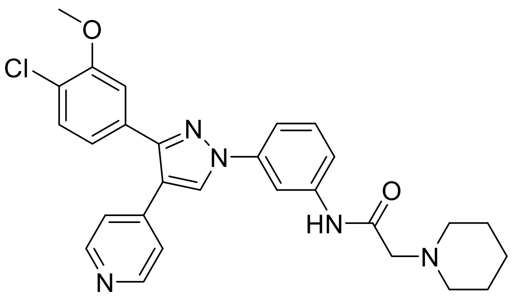
O
Cl
N
N
HN
O
N
N

Compound 1a

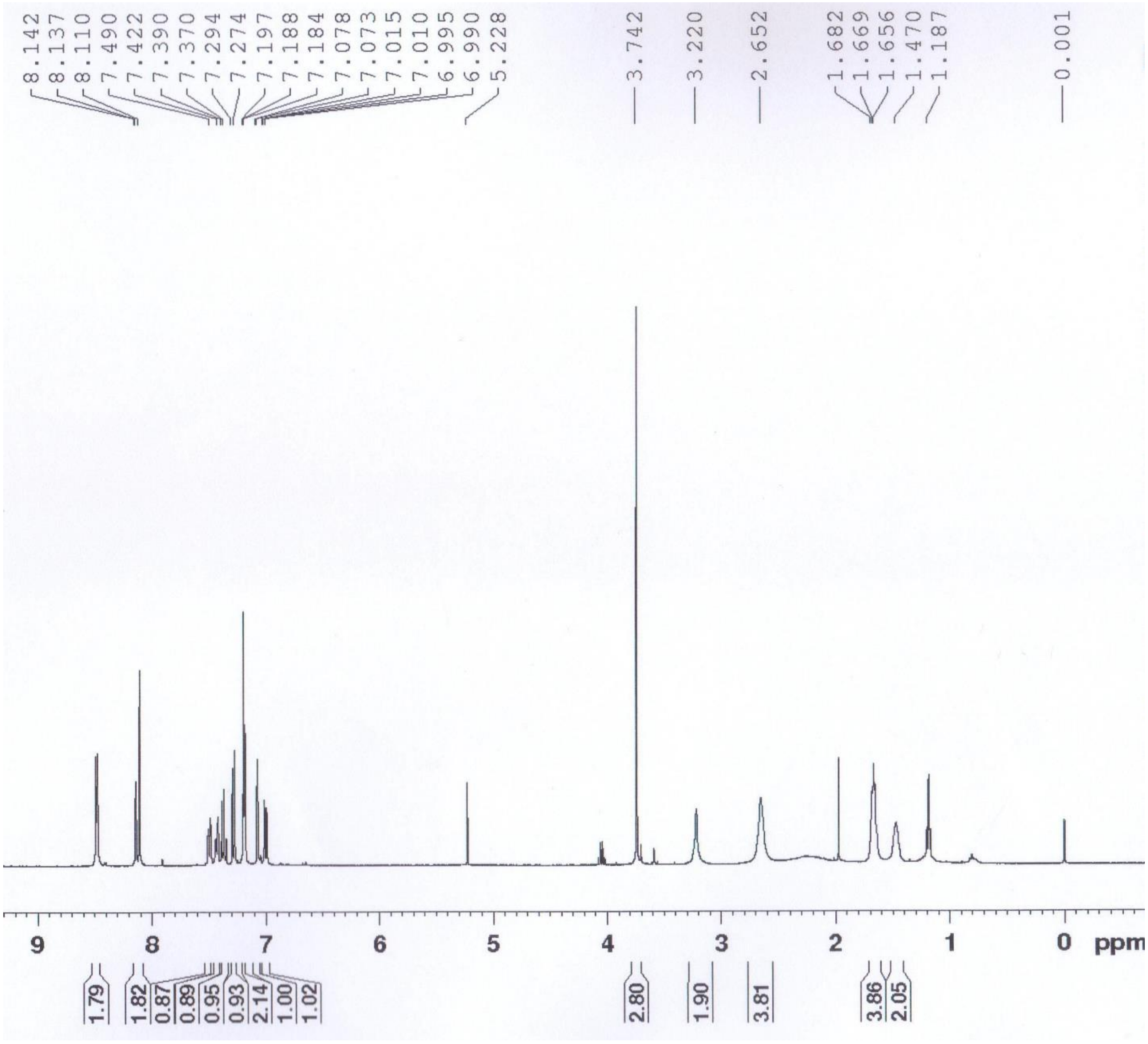
8.142
8.137
8.110
7.490
7.422
7.390
7.370
7.294
7.274
7.197
7.188
7.184
7.078
7.073
7.015
7.010
6.995
6.990
5.228
3.742
3.220
2.652
1.682
1.669
1.656
1.470
1.187
0.001
9
8
7
6
5
4
3
2
1
0
ppm
1.79
1.82
0.87
0.89
0.95
0.93
2.14
1.00
1.02
2.80
1.90
3.81
3.86
2.05

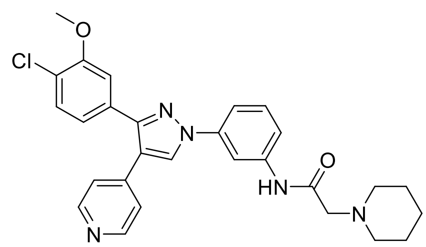

Compound 1a

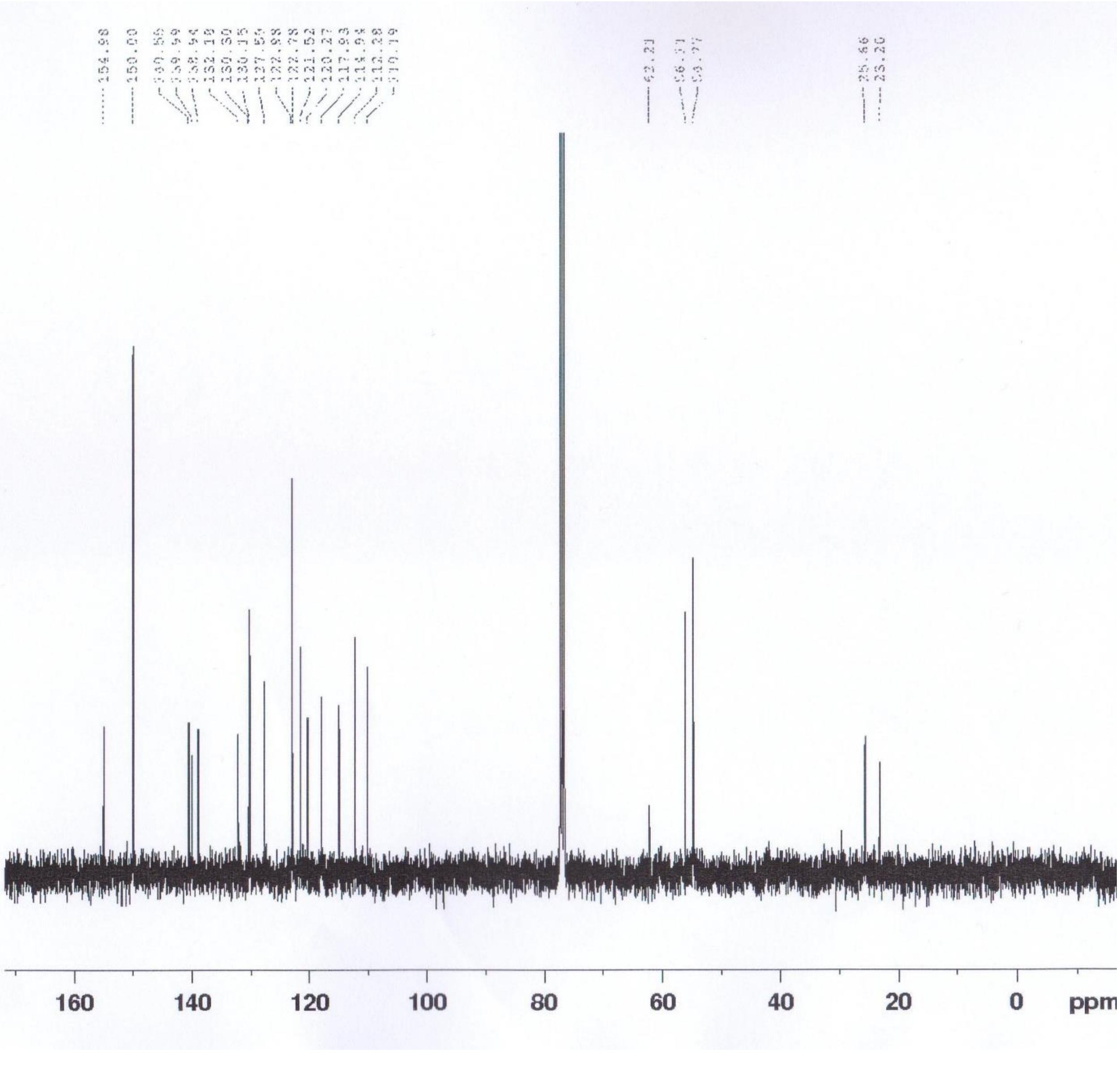

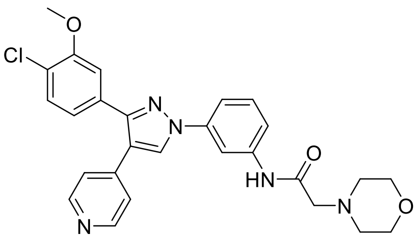
O
Cl
N
N
HN
O
N
O
N

Compound 1b

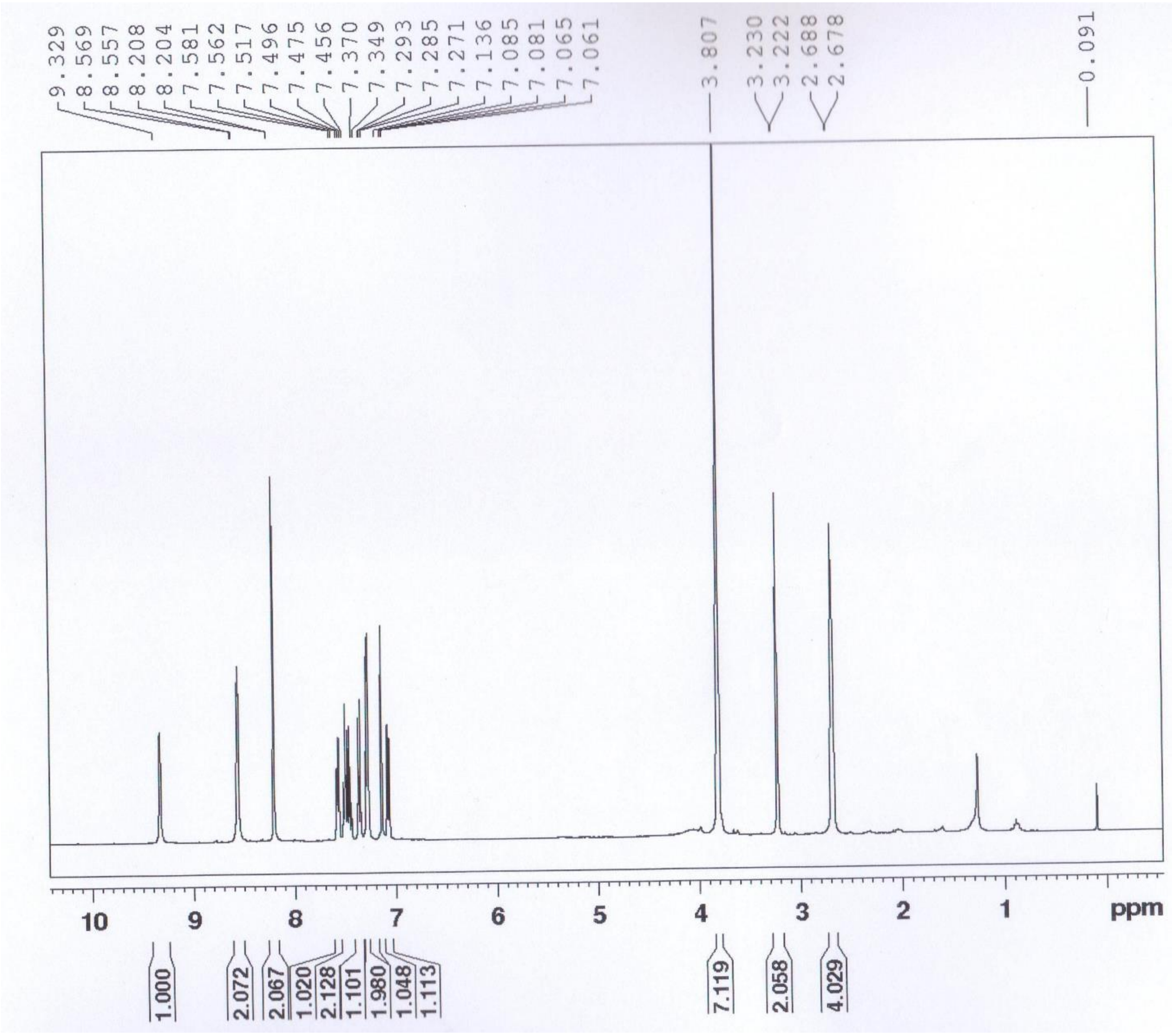
9.329
8.569
8.557
8.208
8.204
7.581
7.562
7.517
7.496
7.475
7.456
7.370
7.349
7.293
7.285
7.271
7.136
7.085
7.081
7.065
7.061
3.807
3.230
3.222
2.688
2.678
0.091
10
9
8
7
6
5
4
3
2
1
ppm
1.000
2.072
2.067
1.020
2.128
1.101
1.980
1.048
1.113
7.119
2.058
4.029

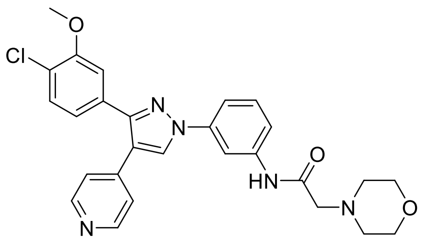

Compound 1b

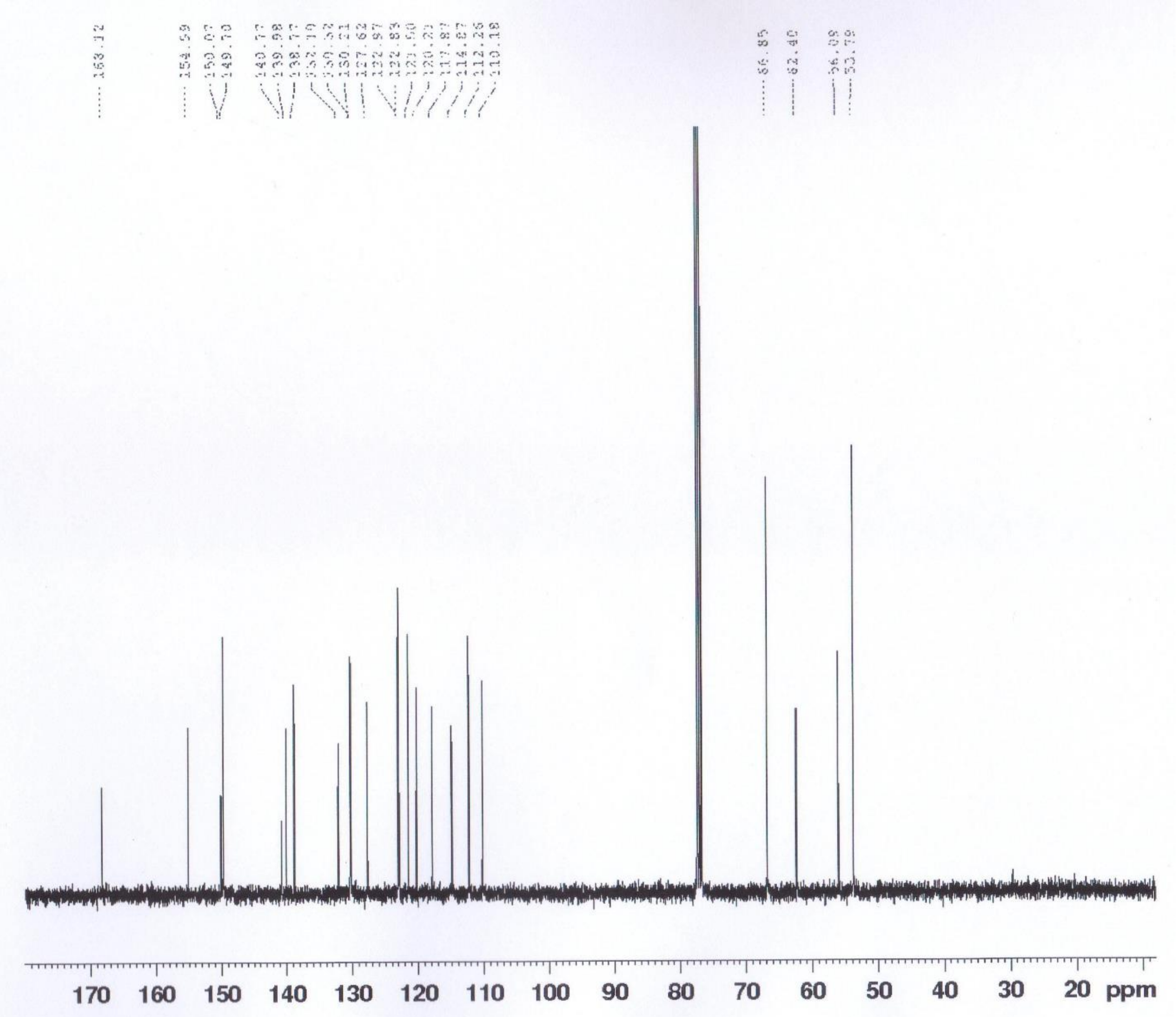
154.59
149.70
139.98
130.21
127.62
122.83
114.87
112.26
110.18
62.40
56.09
53.79
170
160
150
140
130
120
110
100
90
80
70
60
50
40
30
20
ppm

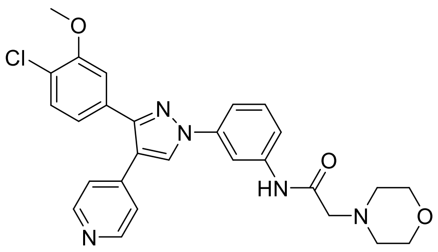

Compound 1b
Chemical Formula: $C_{27}H_{26}ClN_5O_3$
m/z + H^+: 505.1

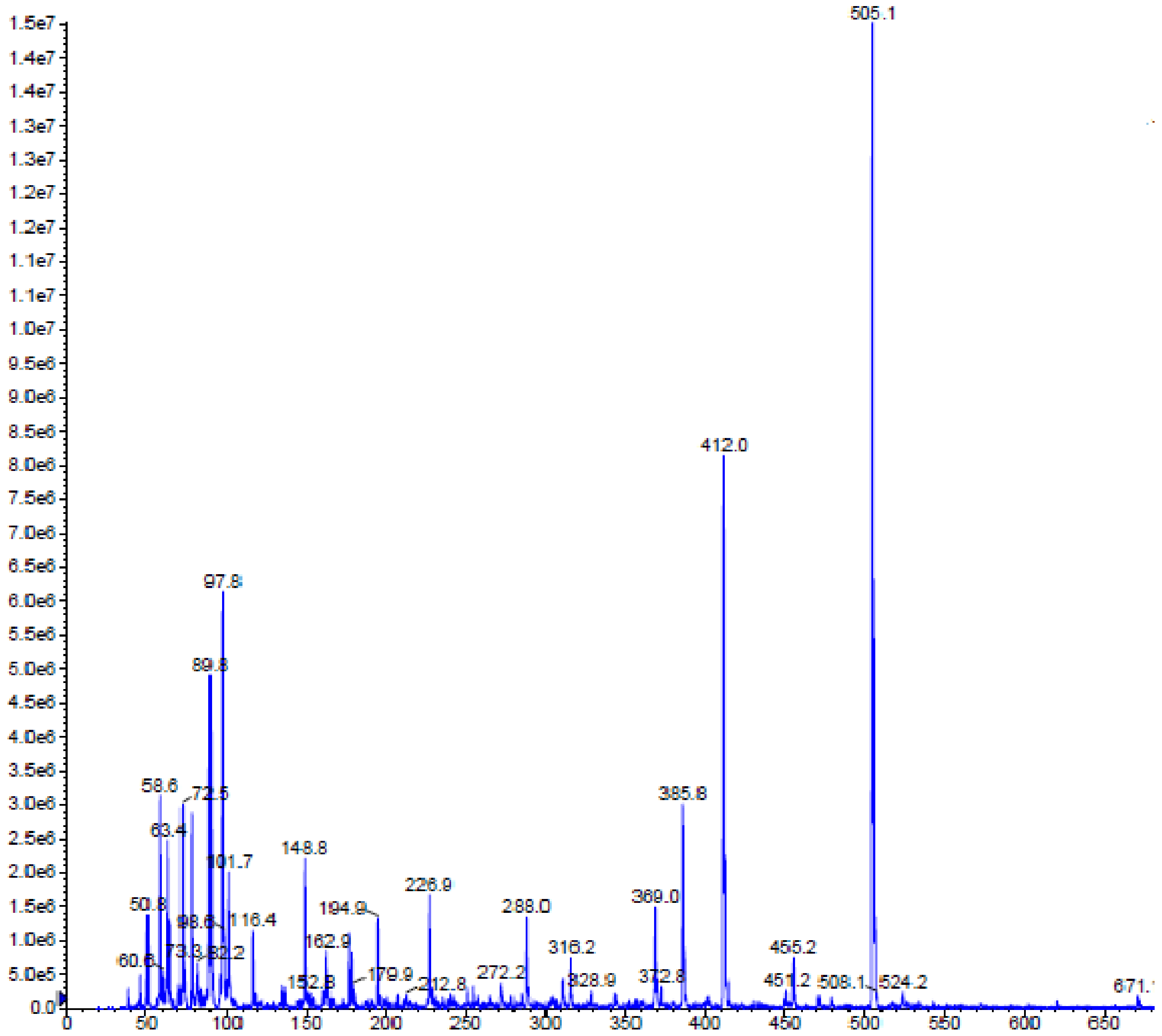

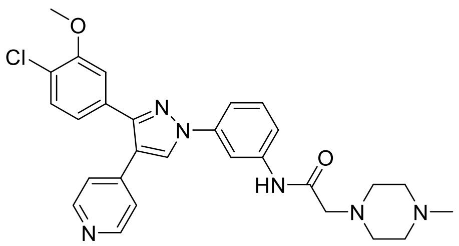

Compound 1c

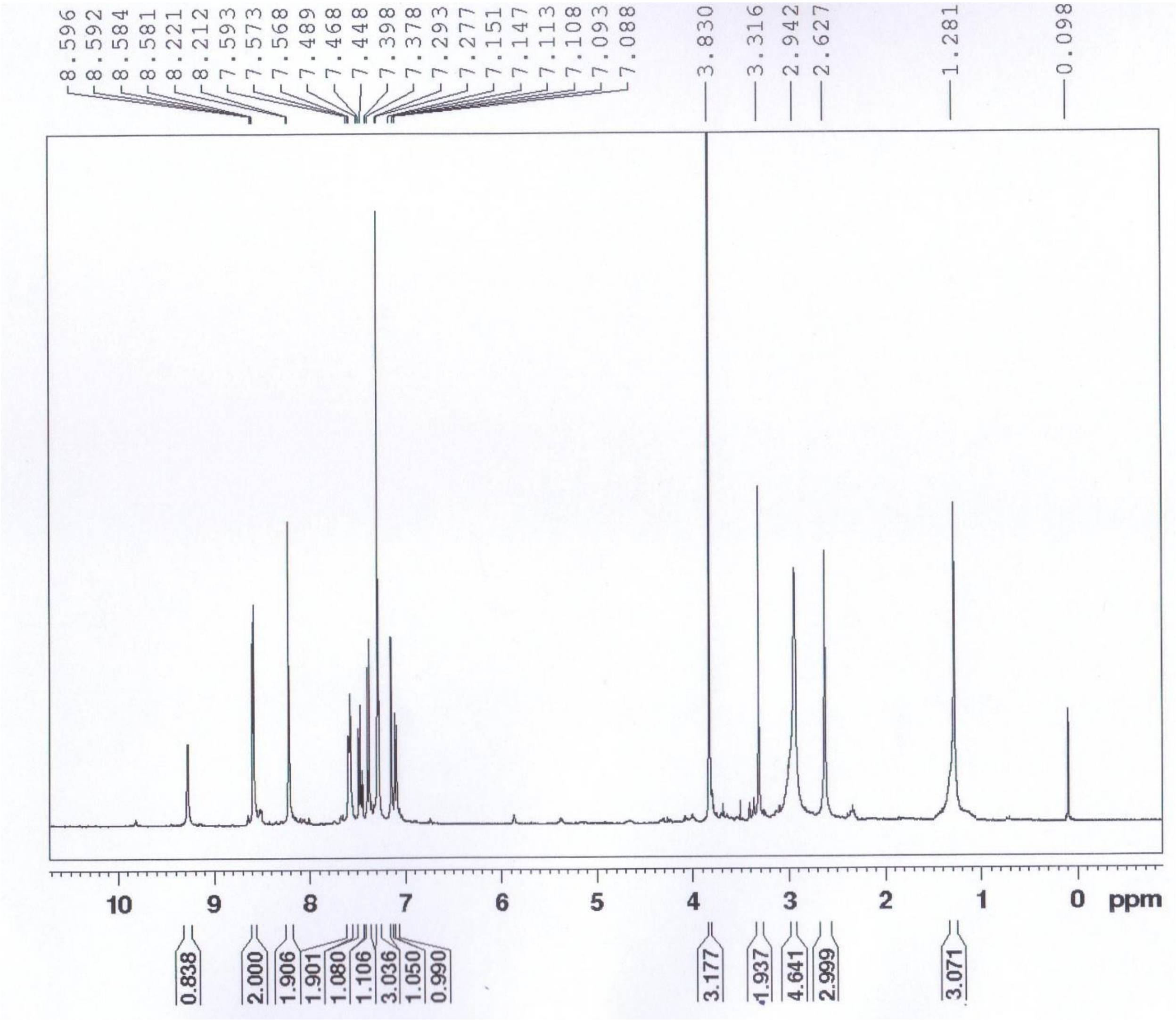
8.596
8.592
8.584
8.581
8.221
8.212
7.593
7.573
7.568
7.489
7.468
7.448
7.398
7.378
7.293
7.277
7.151
7.147
7.113
7.108
7.093
7.088
3.830
3.316
2.942
2.627
1.281
0.098
10
9
8
7
6
5
4
3
2
1
0
ppm
0.838
2.000
1.906
1.901
1.080
1.106
3.036
1.050
0.990
3.177
1.937
4.641
2.999
3.071

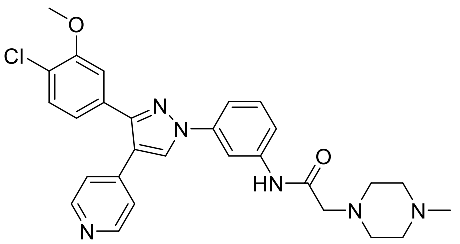
O
Cl
N
N
HN
O
N
N
N

Compound 1c

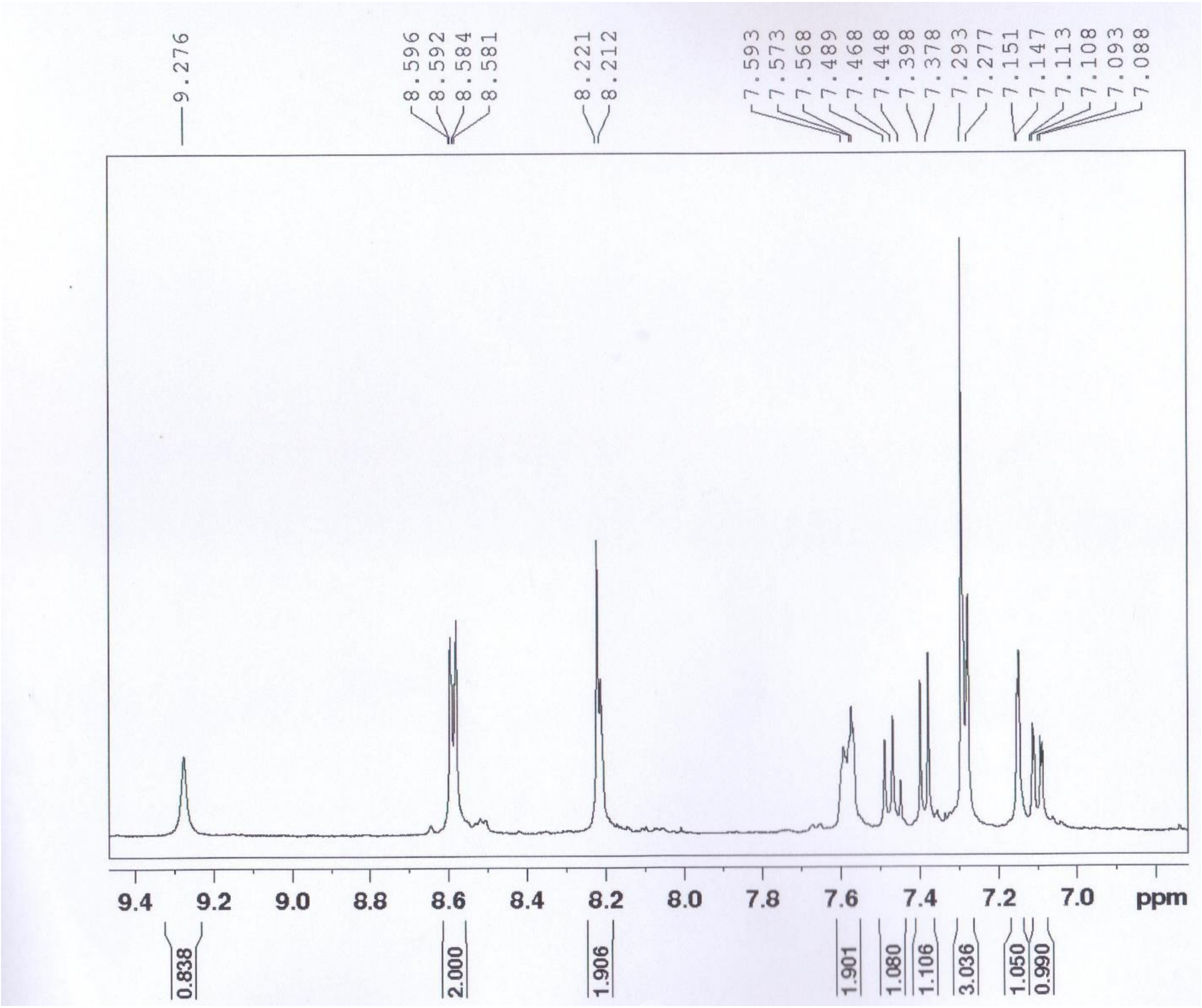
9.276
8.596
8.592
8.584
8.581
8.221
8.212
7.593
7.573
7.568
7.489
7.468
7.448
7.398
7.378
7.293
7.277
7.151
7.147
7.113
7.108
7.093
7.088
9.4
9.2
9.0
8.8
8.6
8.4
8.2
8.0
7.8
7.6
7.4
7.2
7.0
ppm
0.838
2.000
1.906
1.901
1.080
1.106
3.036
1.050
0.990

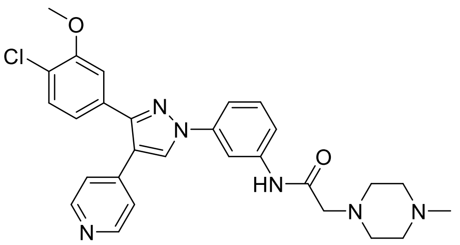

Compound 1c

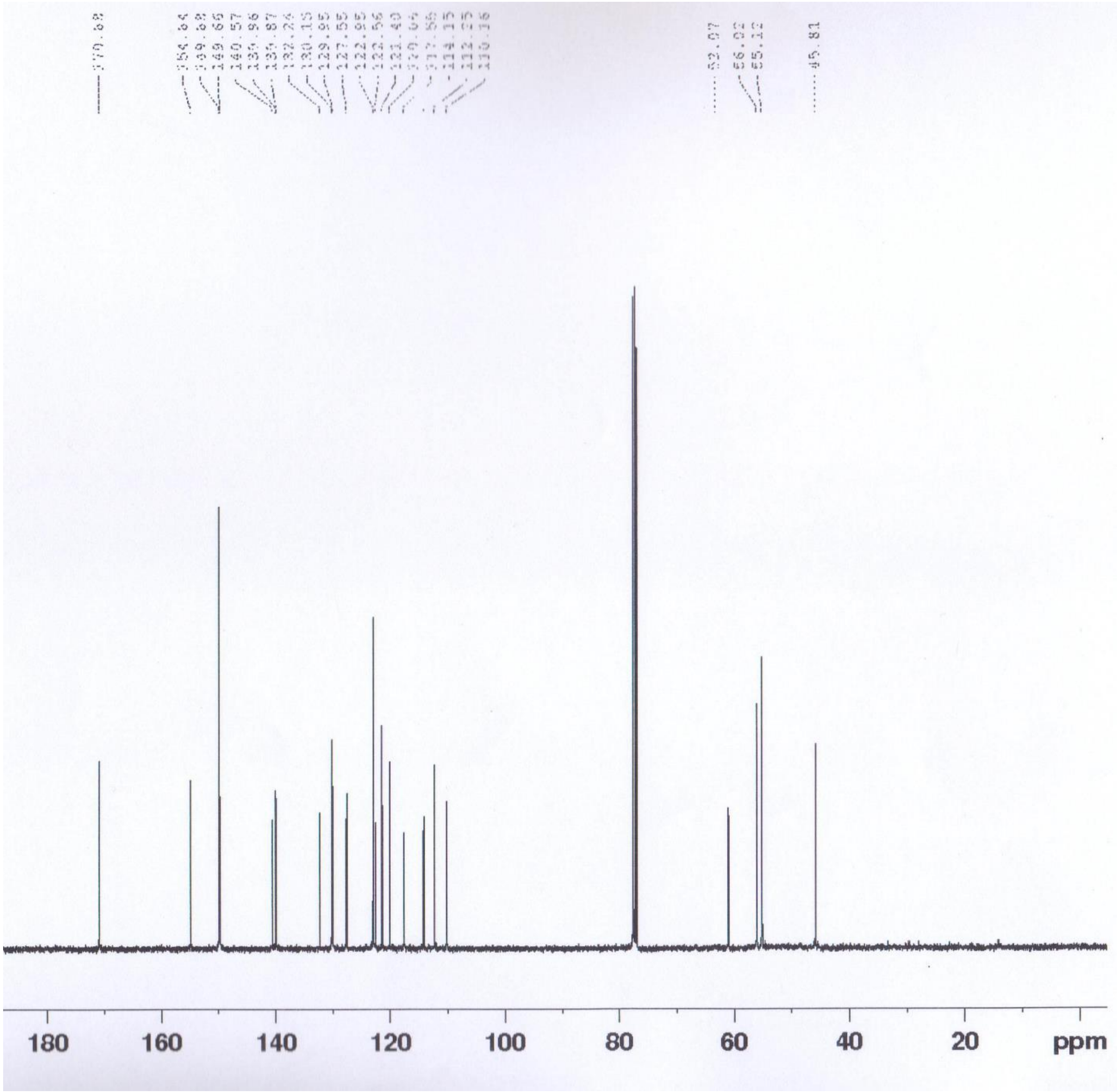

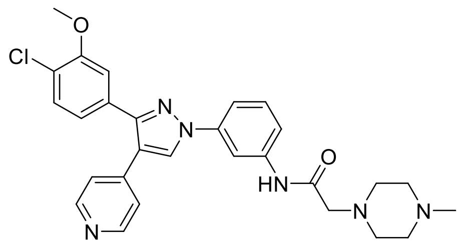
O
Cl
N
N
HN
O
N
N
N

Compound 1c

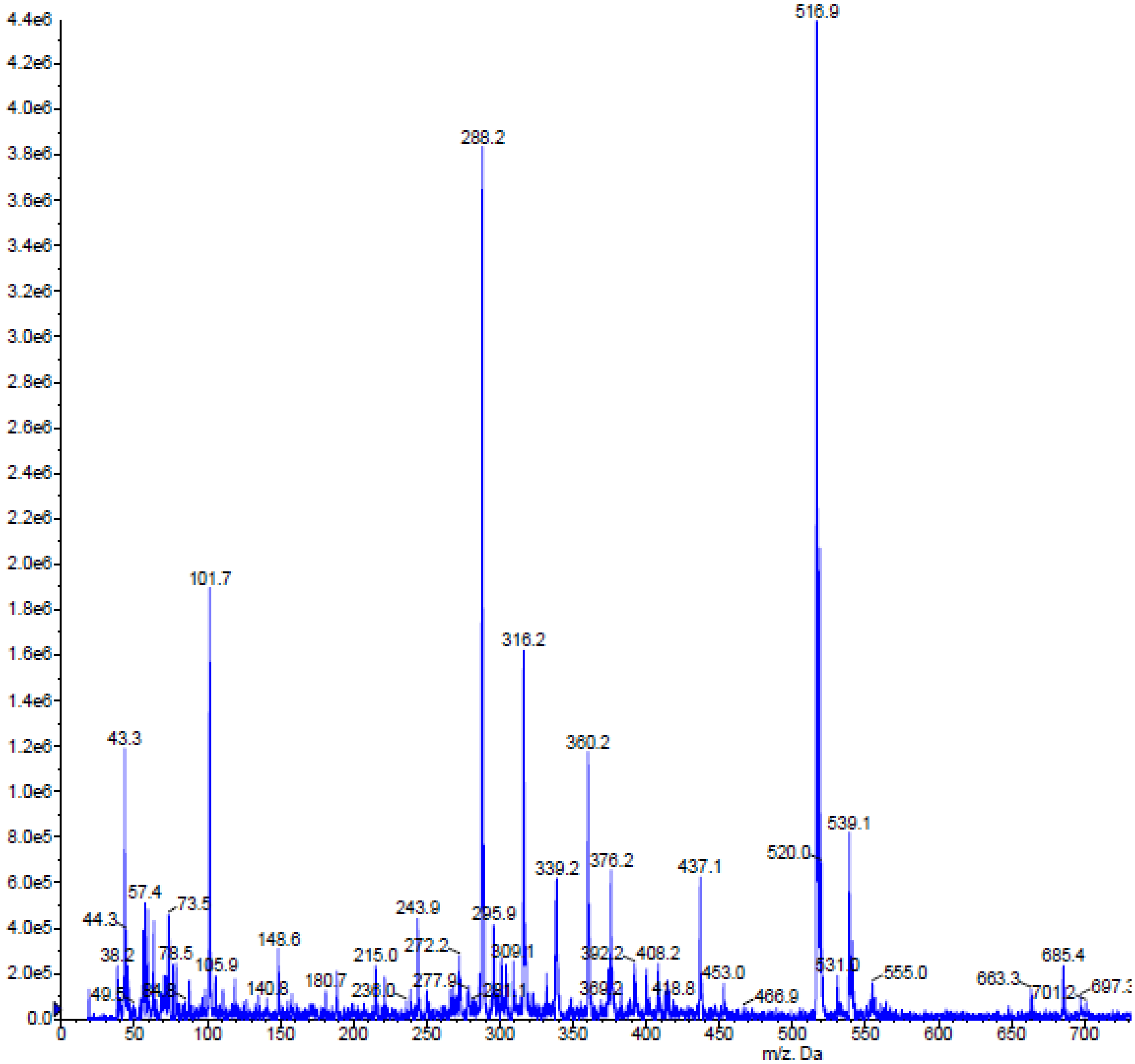
516.9
288.2
101.7
316.2
43.3
360.2
539.1
520.0
339.2
376.2
437.1
57.4
73.5
44.3
243.9
295.9
148.6
38.2
78.5
105.9
215.0
272.2
309.1
392.2
408.2
453.0
531.0
555.0
663.3
685.4
701.2
697.3
49.5
140.8
180.7
230.0
277.9
369.2
418.8
466.9
m/z, Da

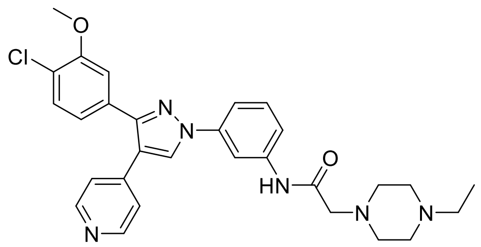
O
Cl
N
N
N
HN
O
N
N

Compound 1d

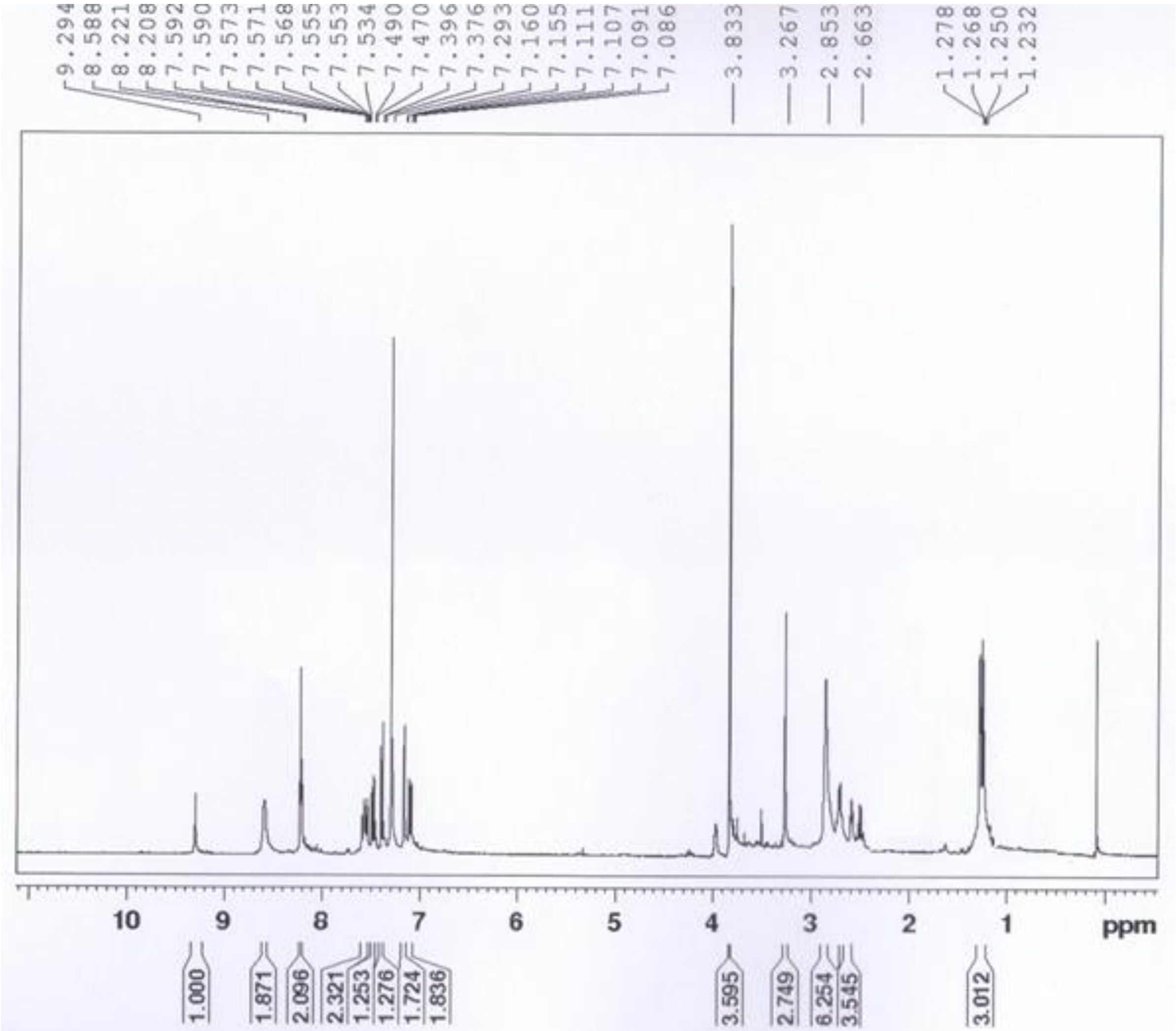
9.294
8.588
8.221
8.208
7.592
7.590
7.573
7.571
7.568
7.555
7.553
7.534
7.490
7.470
7.396
7.376
7.293
7.160
7.155
7.111
7.107
7.091
7.086
3.833
3.267
2.853
2.663
1.278
1.268
1.250
1.232
10
9
8
7
6
5
4
3
2
1
ppm
1.000
1.871
2.096
2.321
1.253
1.276
1.724
1.836
3.595
2.749
6.254
3.545
3.012

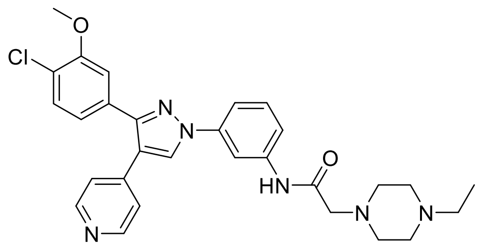

Cl
O
N
N
N
HN
O
N
N

Compound 1d

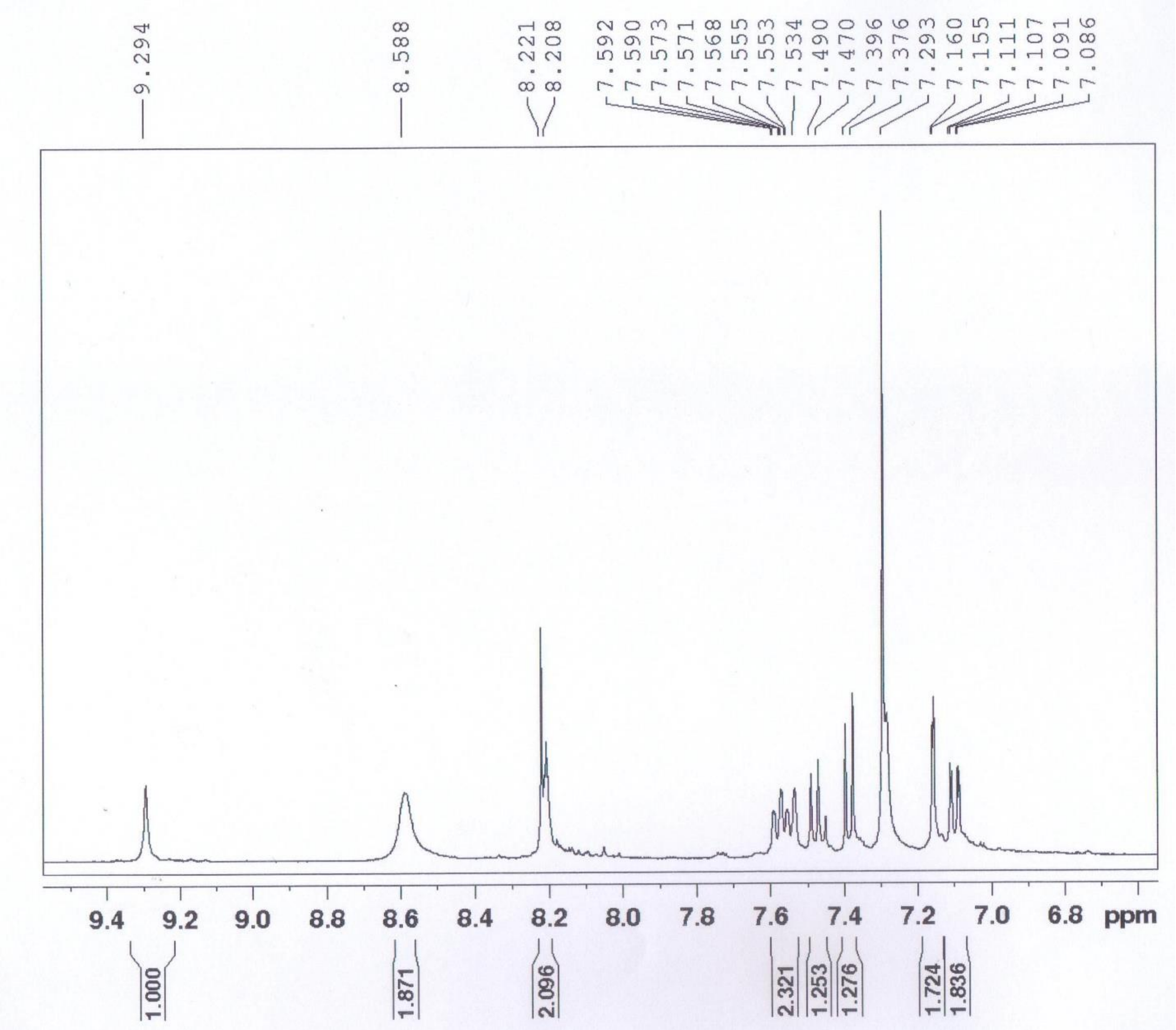

9.294
8.588
8.221
8.208
7.592
7.590
7.573
7.571
7.568
7.555
7.553
7.534
7.490
7.470
7.396
7.376
7.293
7.160
7.155
7.111
7.107
7.091
7.086
9.4 9.2 9.0 8.8 8.6 8.4 8.2 8.0 7.8 7.6 7.4 7.2 7.0 6.8 ppm
1.000
1.871
2.096
2.321
1.253
1.276
1.724
1.836

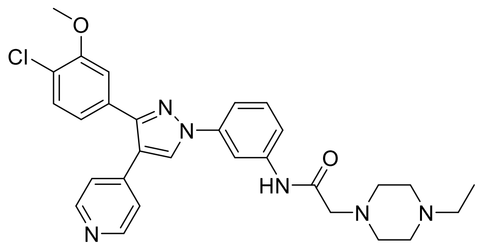

Compound 1d

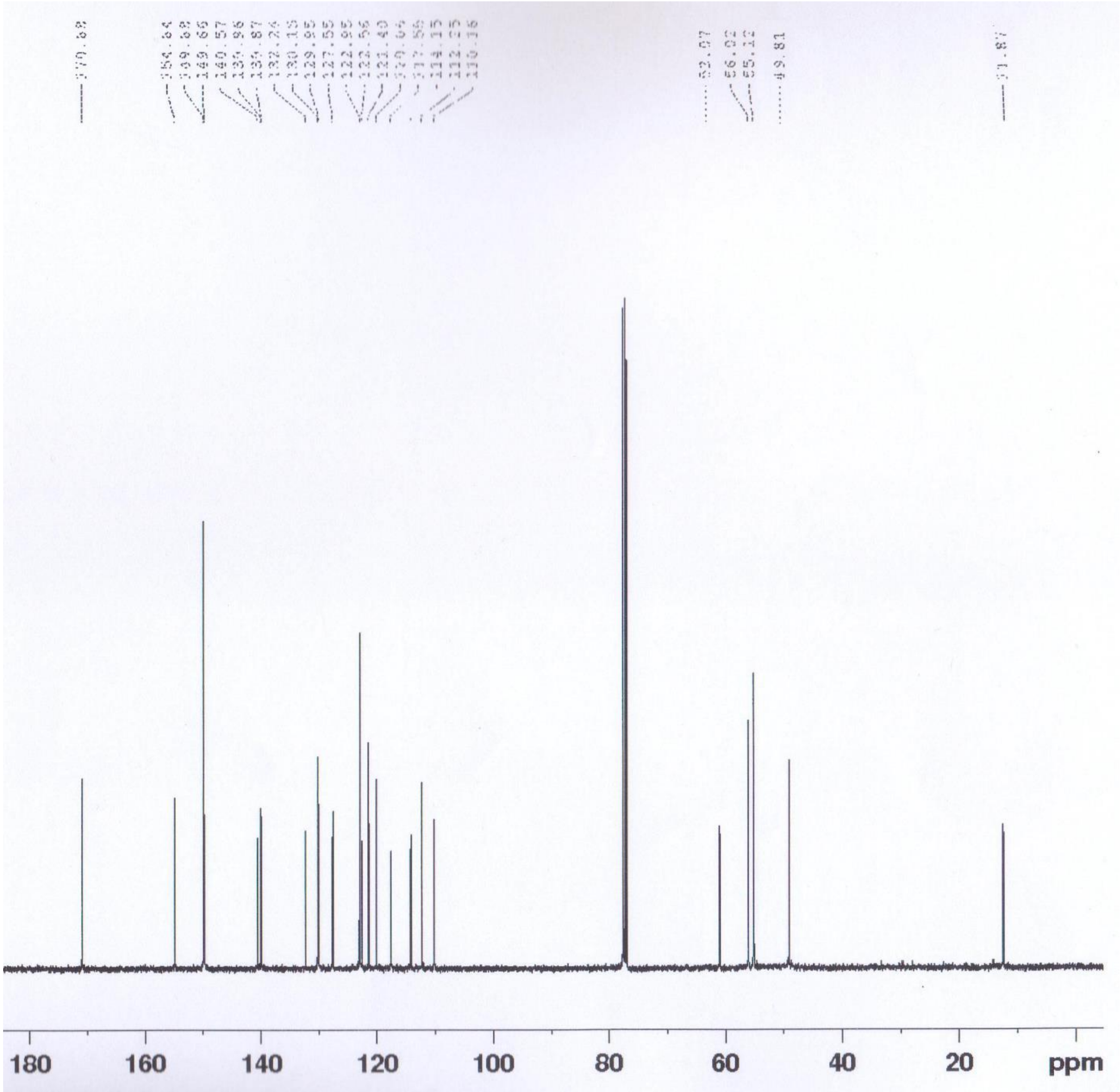

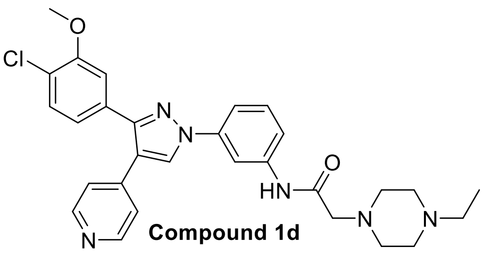

Compound 1d

Chemical Formula: $C_{29}H_{31}ClN_6O_2$
m/z: 531.0

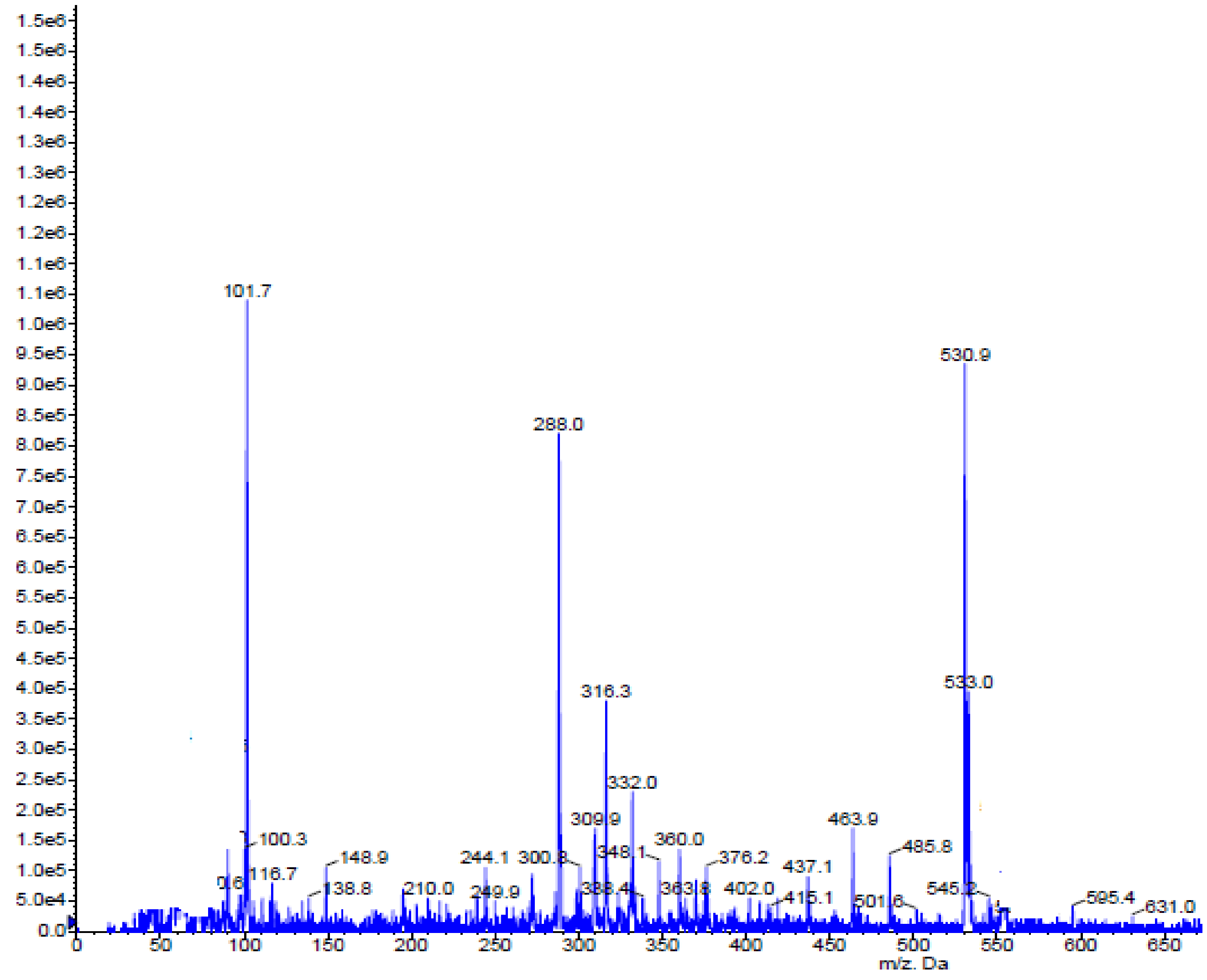

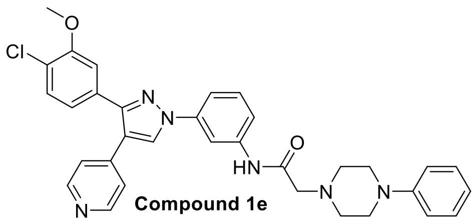

Compound 1e

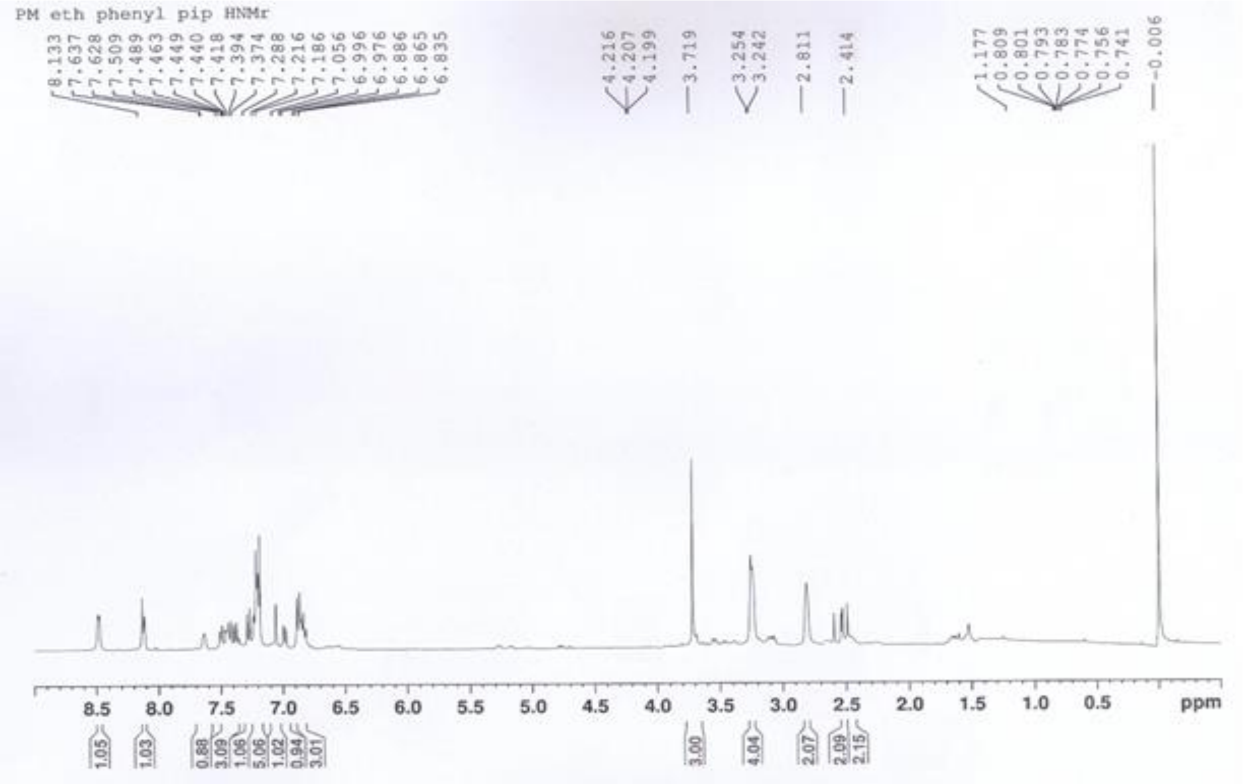

PM eth phenyl pip HNMr
8.133
7.637
7.628
7.509
7.489
7.463
7.449
7.440
7.418
7.394
7.374
7.288
7.216
7.186
7.056
6.996
6.976
6.886
6.865
6.835
4.216
4.207
4.199
3.719
3.254
3.242
2.811
2.414
1.177
0.809
0.801
0.793
0.783
0.774
0.756
0.741
-0.006
8.5 8.0 7.5 7.0 6.5 6.0 5.5 5.0 4.5 4.0 3.5 3.0 2.5 2.0 1.5 1.0 0.5 ppm
1.05
1.03
0.88
3.09
1.06
5.06
1.02
0.94
3.01
3.00
4.04
2.07
2.09
2.15

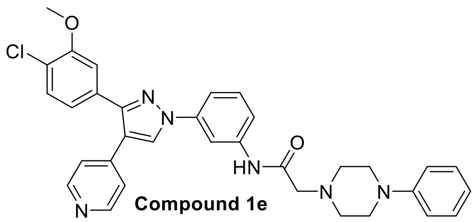

Compound 1e

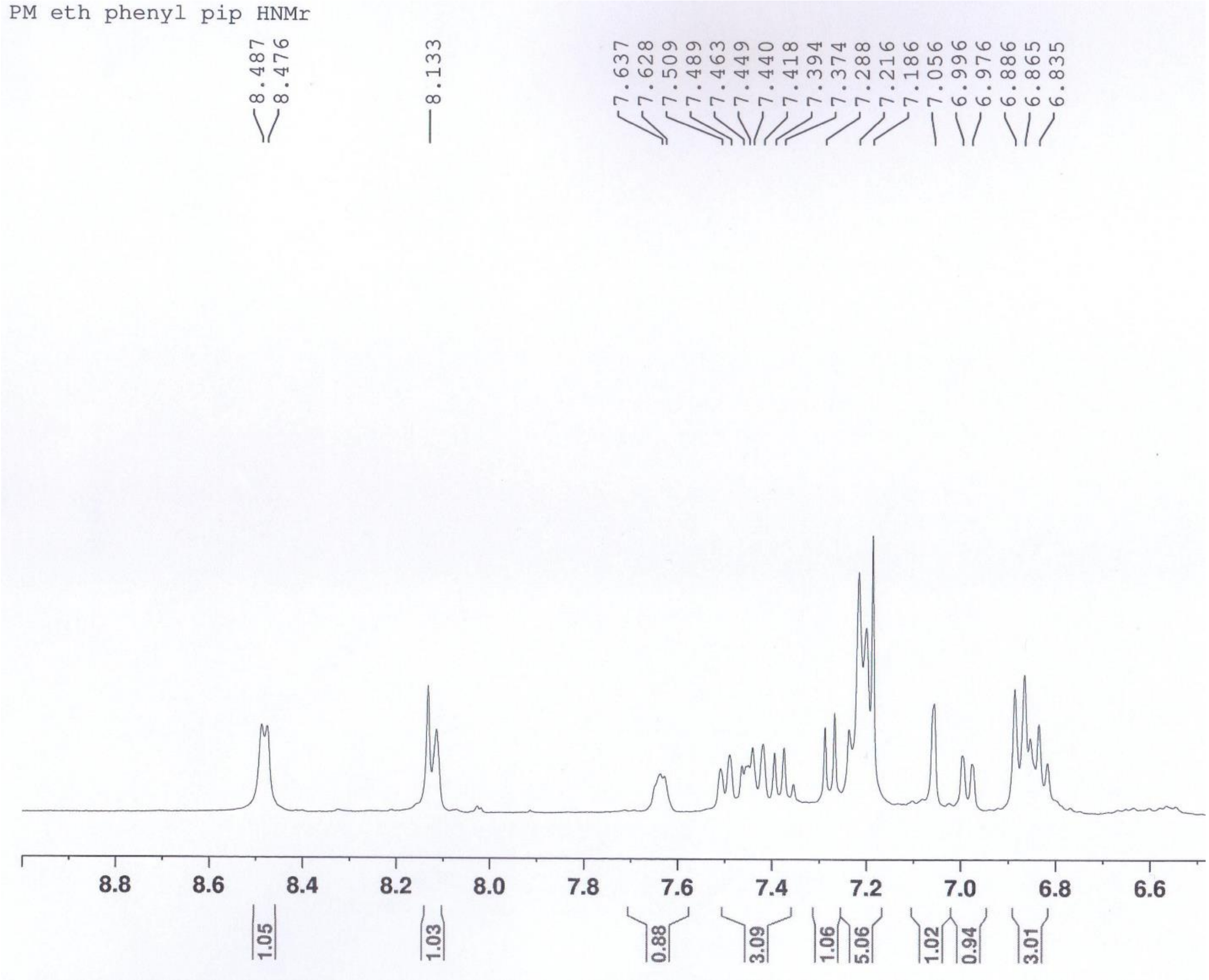
PM eth phenyl pip HNMr
8.487
8.476
8.133
7.637
7.628
7.509
7.489
7.463
7.449
7.440
7.418
7.394
7.374
7.288
7.216
7.186
7.056
6.996
6.976
6.886
6.865
6.835
8.8
8.6
8.4
8.2
8.0
7.8
7.6
7.4
7.2
7.0
6.8
6.6
1.05
1.03
0.88
3.09
1.06
5.06
1.02
0.94
3.01

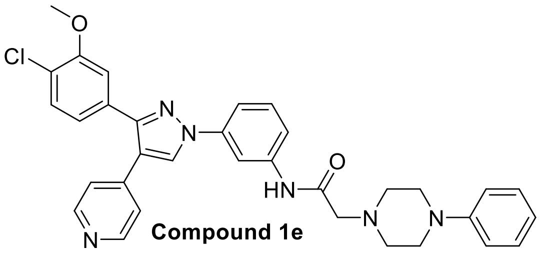

Compound 1e

Chemical Formula: $C_{33}H_{31}ClN_6O_2$

m/z:+ $2H^+$ 579.9

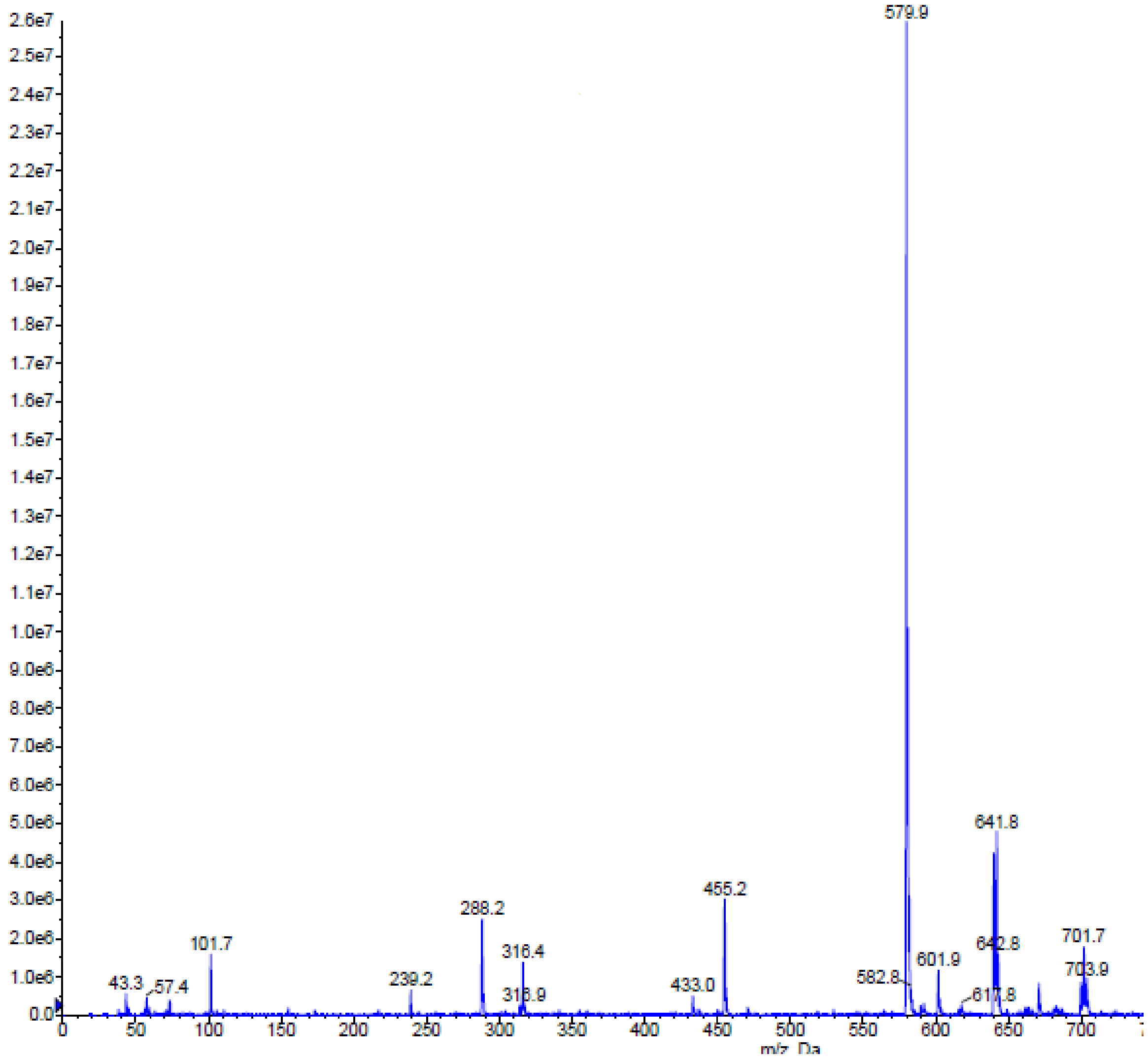

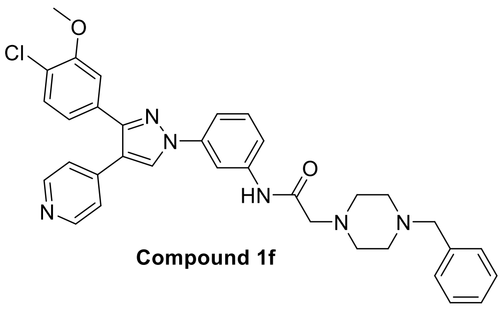
Cl
O
N
N
N
HN
O
N
N

Compound 1f

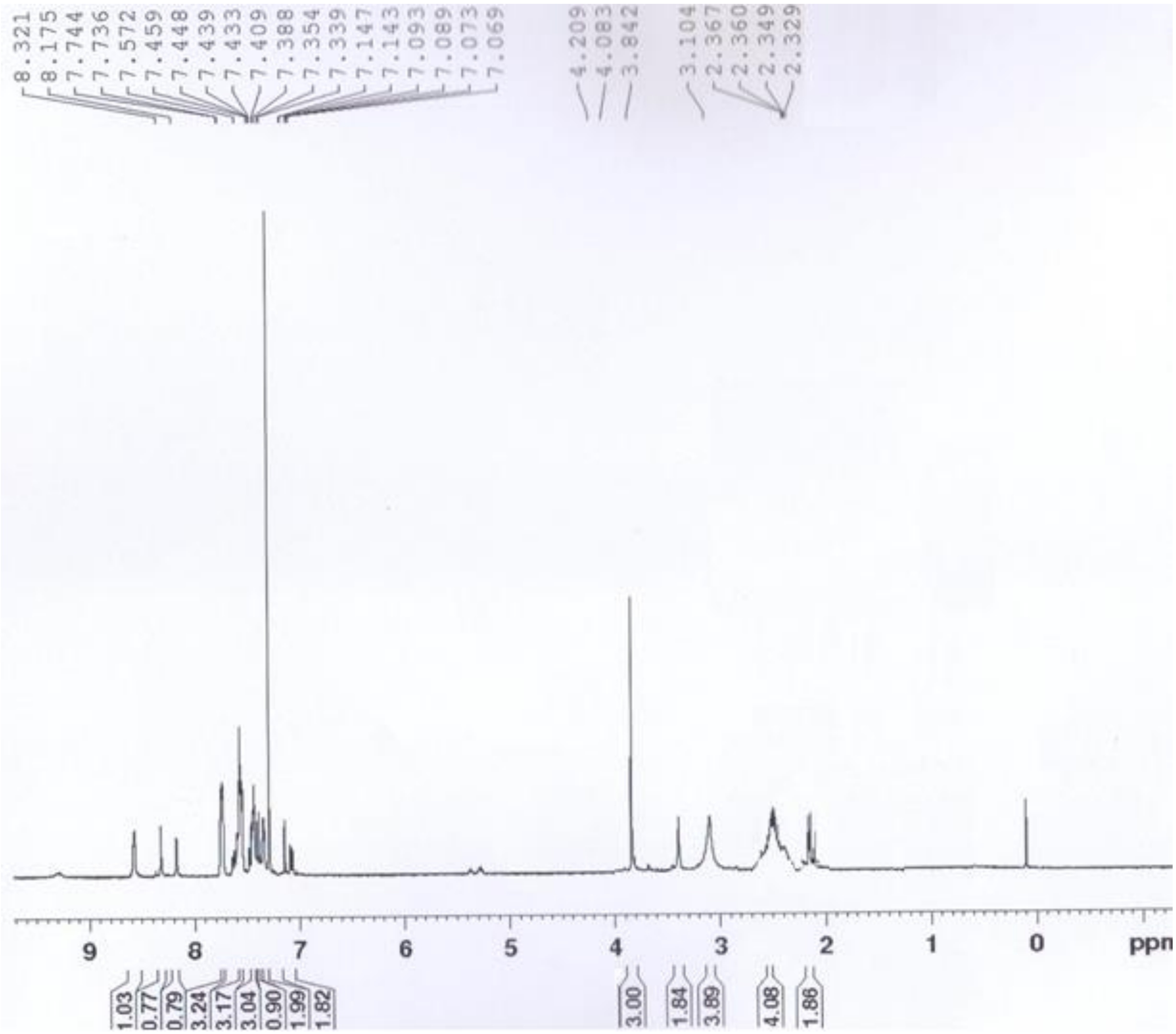
8.321
8.175
7.744
7.736
7.572
7.459
7.448
7.439
7.433
7.409
7.388
7.354
7.339
7.147
7.143
7.093
7.089
7.073
7.069
4.209
4.083
3.842
3.104
2.367
2.360
2.349
2.329
9
8
7
6
5
4
3
2
1
0
ppm
1.03
0.77
0.79
3.24
3.17
3.04
0.90
1.99
1.82
3.00
1.84
3.89
4.08
1.86

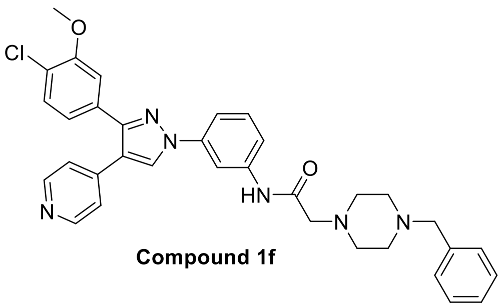
Cl
O
N
N
N
HN
O
N
N

Compound 1f

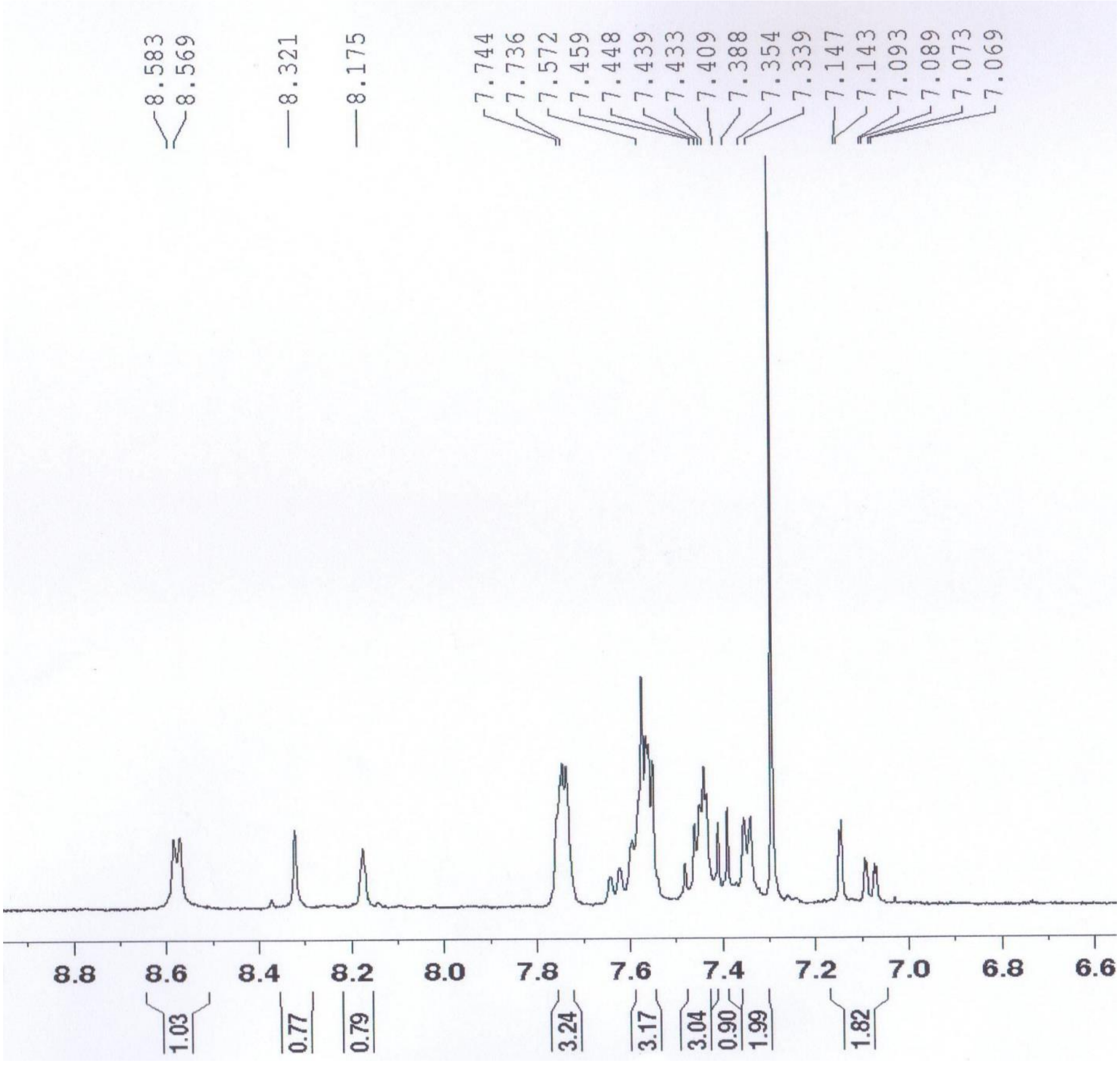
8.583
8.569
8.321
8.175
7.744
7.736
7.572
7.459
7.448
7.439
7.433
7.409
7.388
7.354
7.339
7.147
7.143
7.093
7.089
7.073
7.069
8.8
8.6
8.4
8.2
8.0
7.8
7.6
7.4
7.2
7.0
6.8
6.6
1.03
0.77
0.79
3.24
3.17
3.04
0.90
1.99
1.82

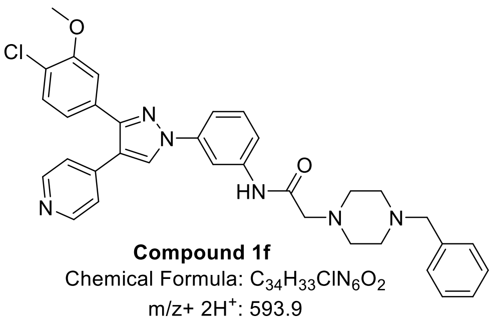

Compound 1f
Chemical Formula: $C_{34}H_{33}ClN_6O_2$
m/z+ 2H$^+$: 593.9

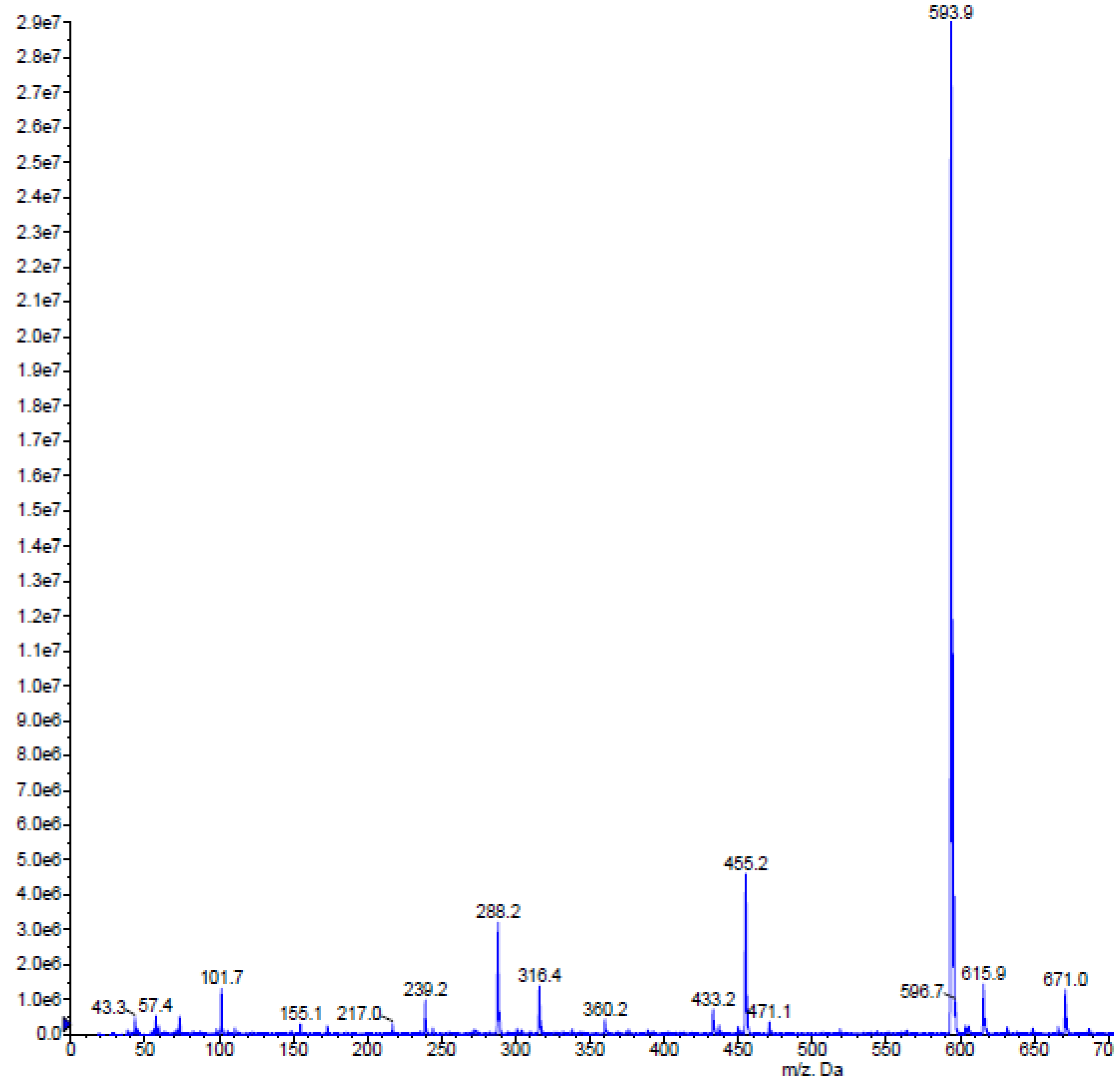

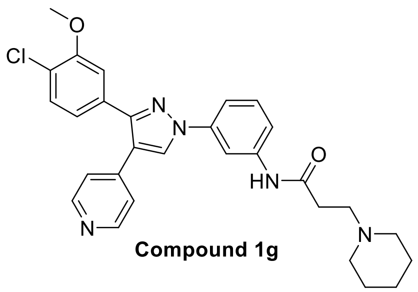
Cl
O
N
N
HN
O
N
N

Compound 1g

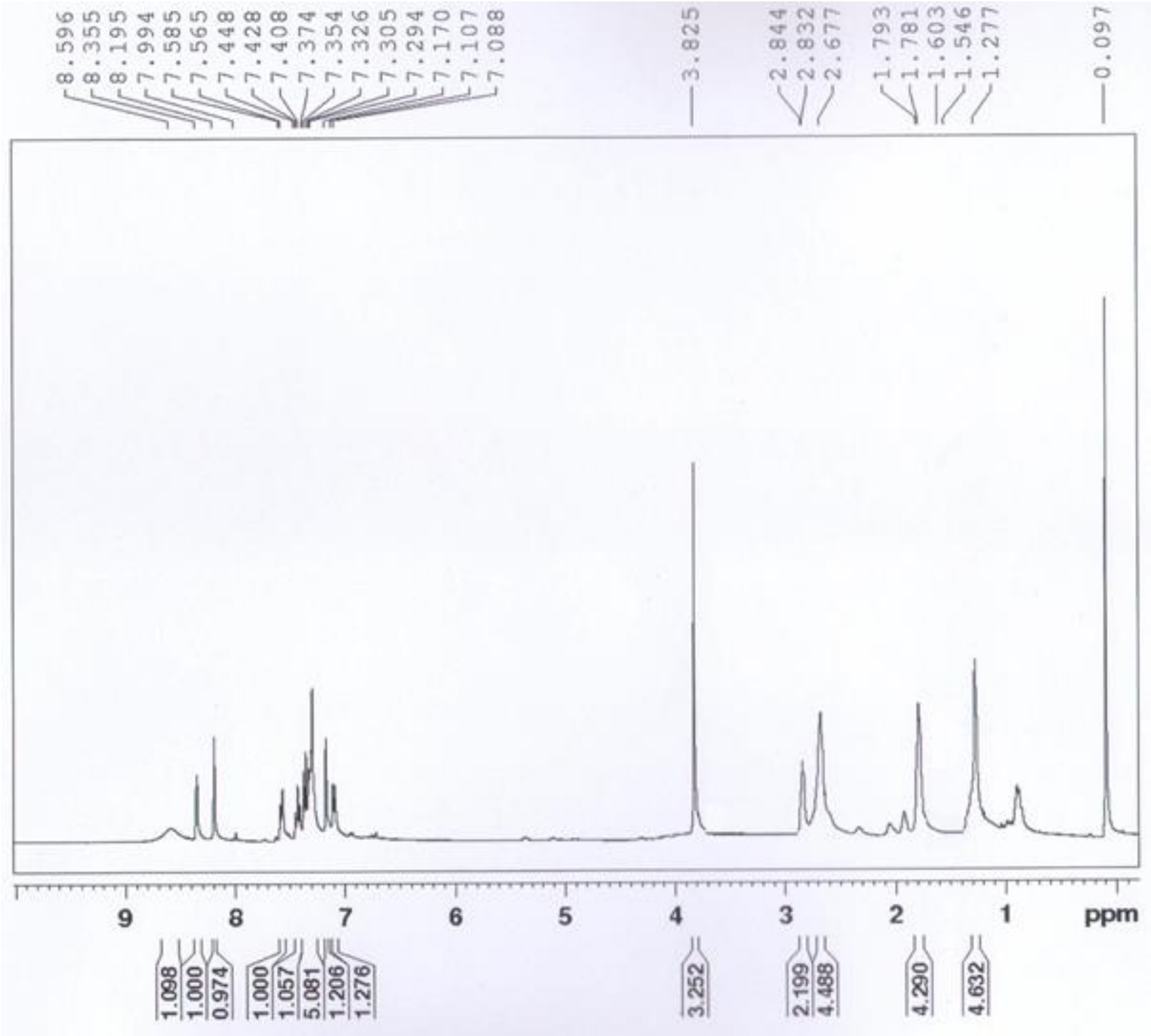
8.596
8.355
8.195
7.994
7.585
7.565
7.448
7.428
7.408
7.374
7.354
7.326
7.305
7.294
7.170
7.107
7.088
3.825
2.844
2.832
2.677
1.793
1.781
1.603
1.546
1.277
0.097
9
8
7
6
5
4
3
2
1
ppm
1.098
1.000
0.974
1.000
1.057
5.081
1.206
1.276
3.252
2.199
4.488
4.290
4.632

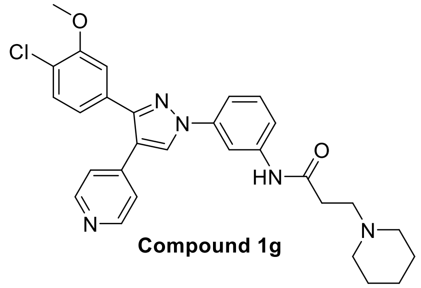

Cl
O
N
N
HN
O
N
N
Compound 1g

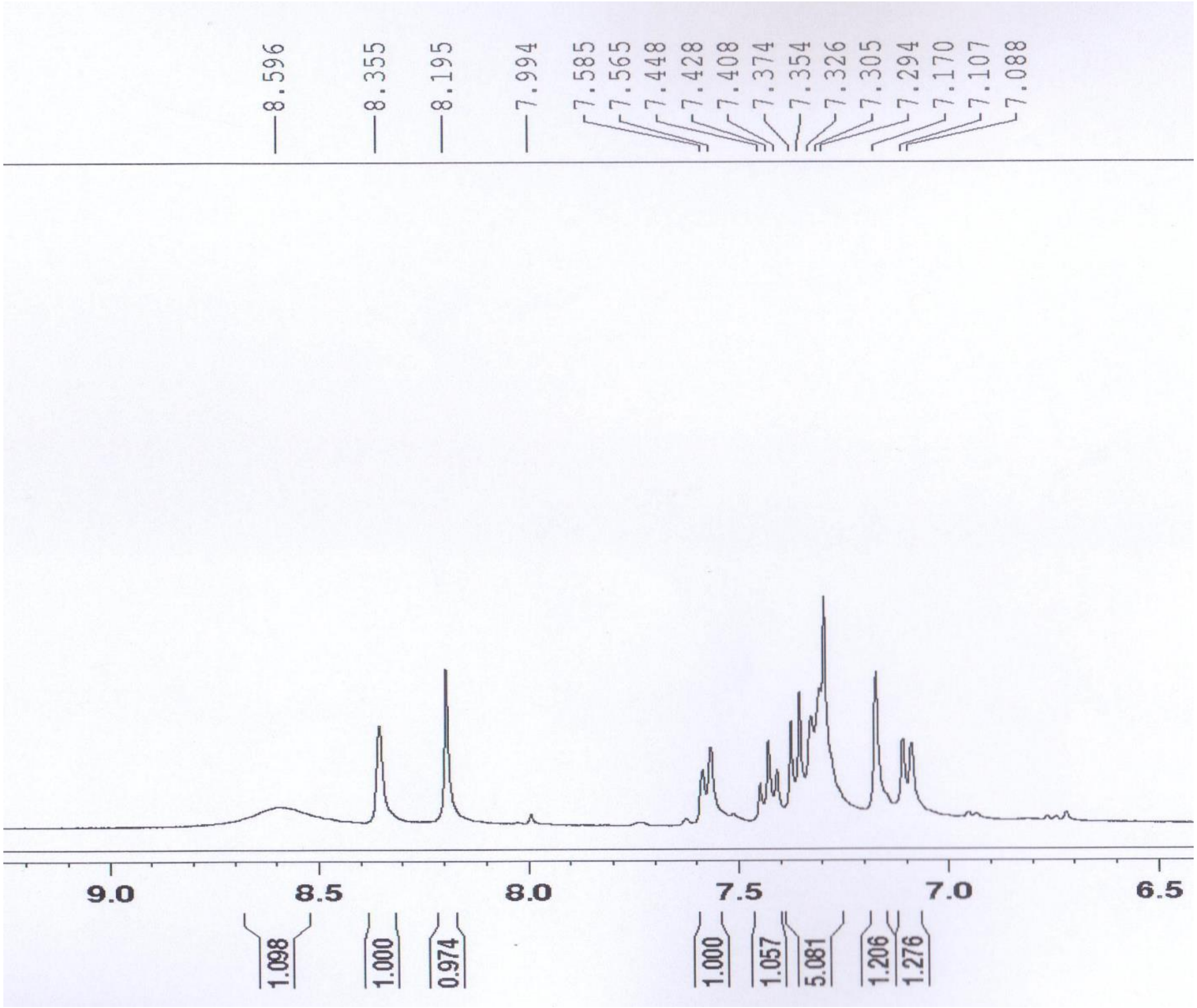

8.596
8.355
8.195
7.994
7.585
7.565
7.448
7.428
7.408
7.374
7.354
7.326
7.305
7.294
7.170
7.107
7.088
9.0
8.5
8.0
7.5
7.0
6.5
1.098
1.000
0.974
1.000
1.057
5.081
1.206
1.276

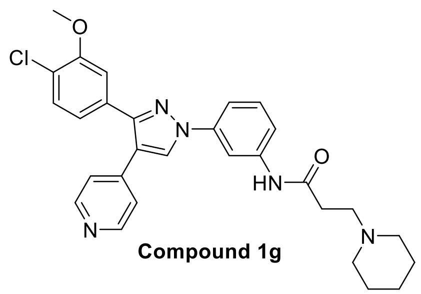

Compound 1g

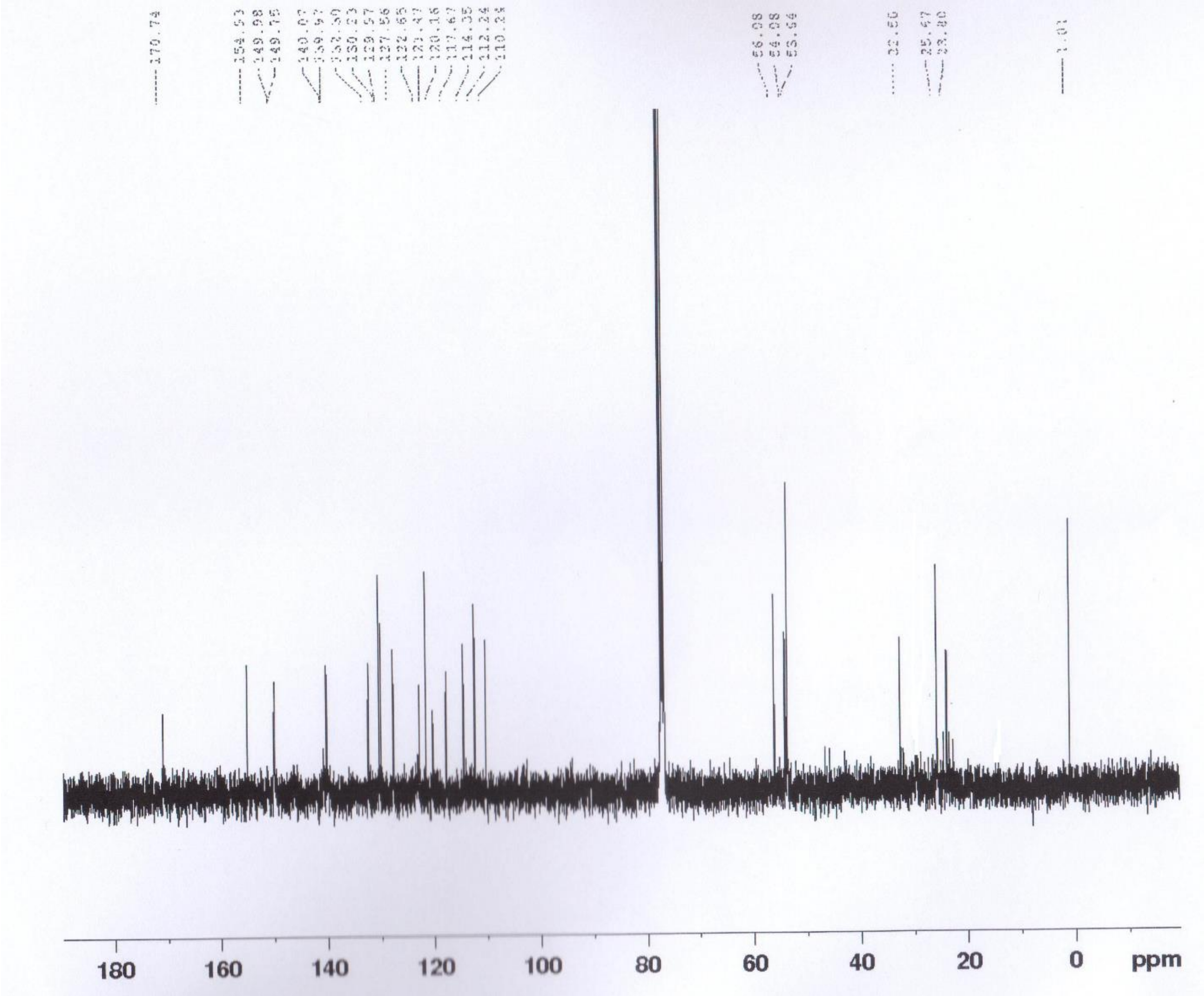

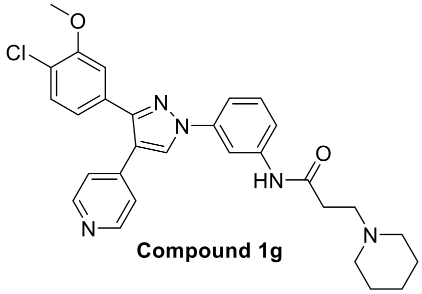

Compound 1g

Chemical Formula: $C_{29}H_{30}ClN_5O_2$
m/z+ 2: 517.21

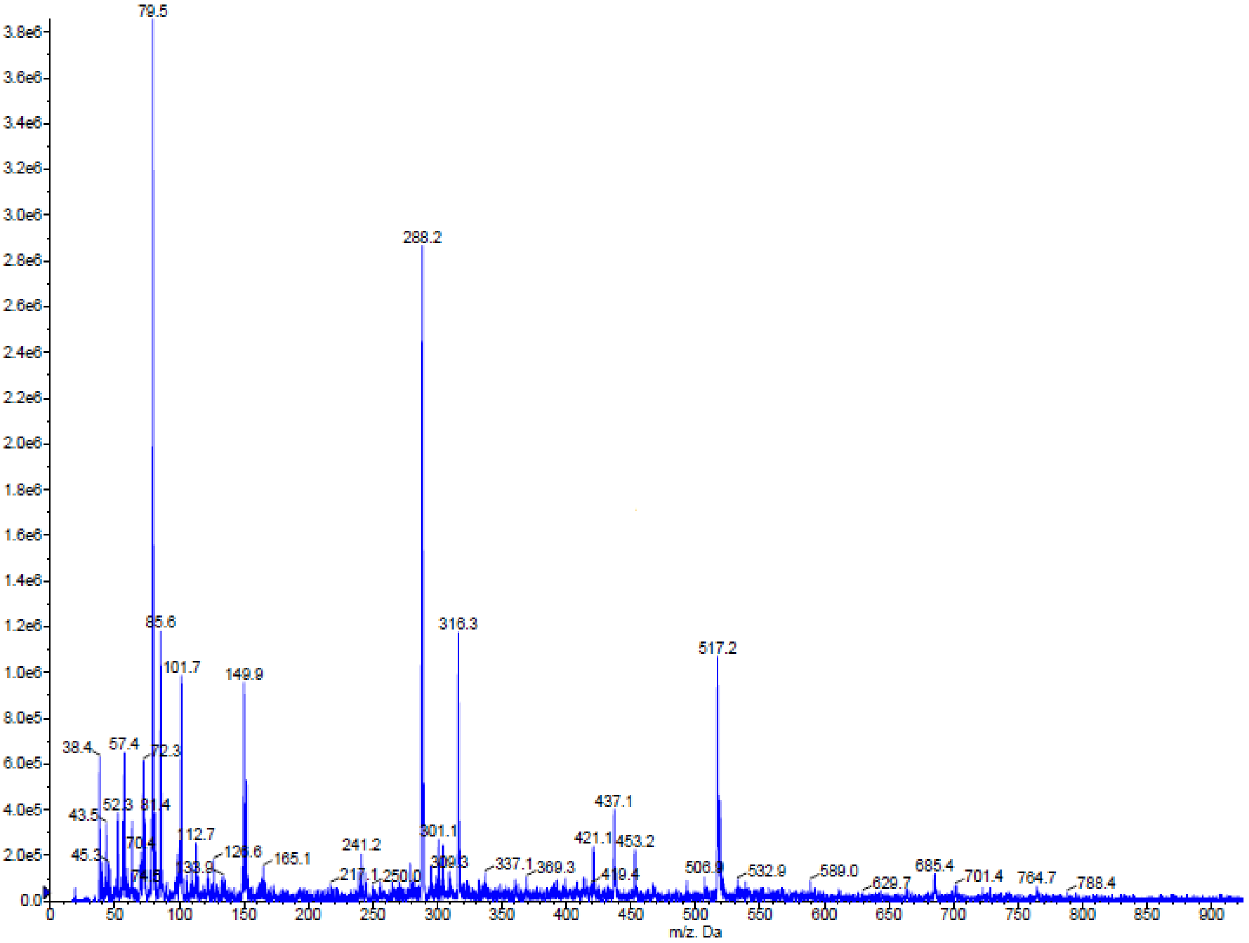

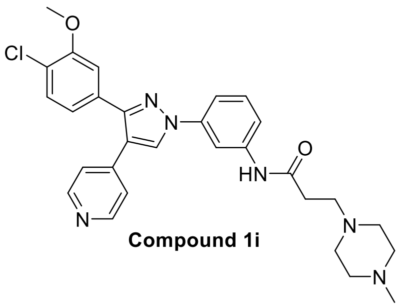

Compound 1i

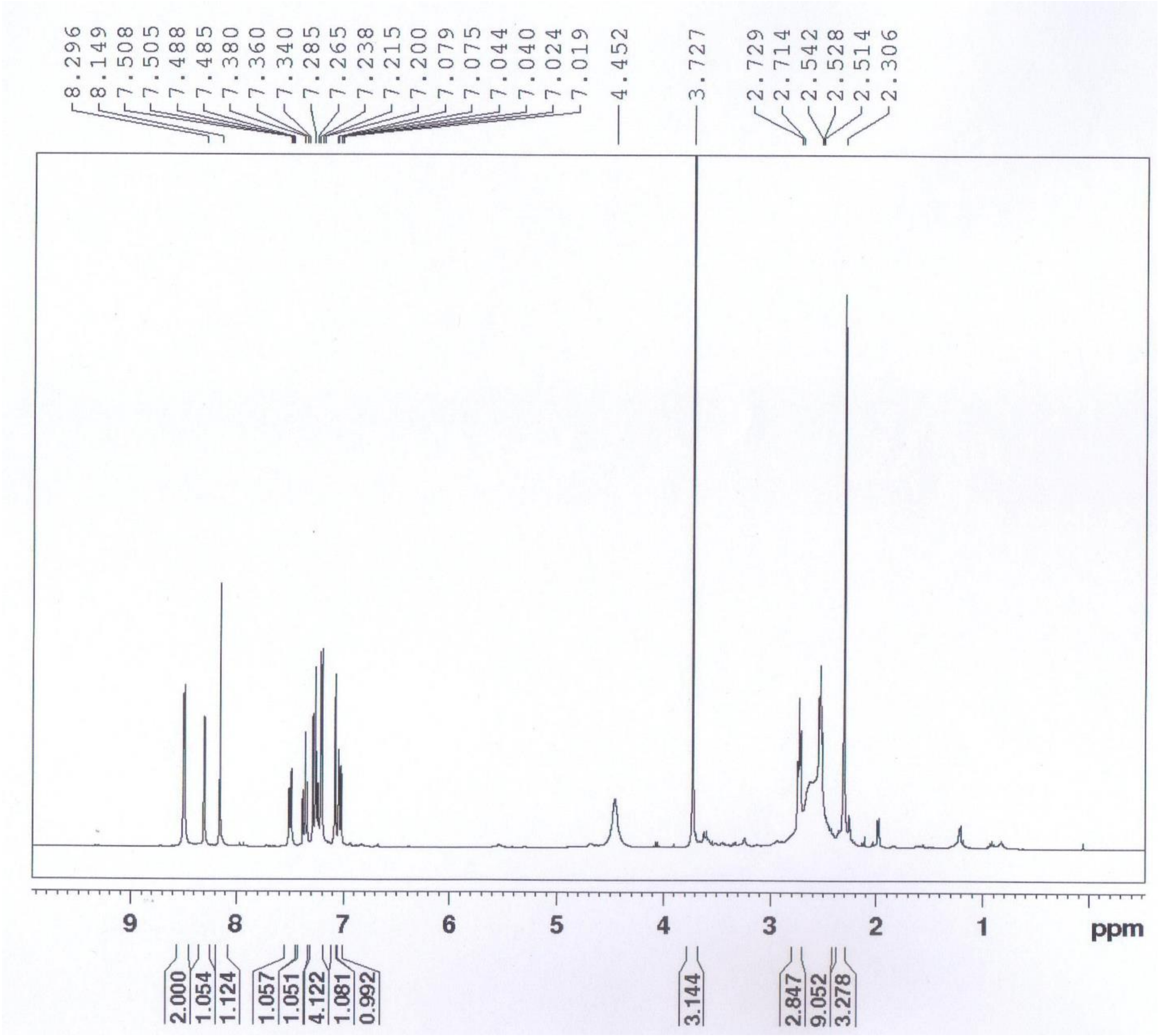

8.296
8.149
7.508
7.505
7.488
7.485
7.380
7.360
7.340
7.285
7.265
7.238
7.215
7.200
7.079
7.075
7.044
7.040
7.024
7.019
4.452
3.727
2.729
2.714
2.542
2.528
2.514
2.306
9
8
7
6
5
4
3
2
1
ppm
2.000
1.054
1.124
1.057
1.051
4.122
1.081
0.992
3.144
2.847
9.052
3.278

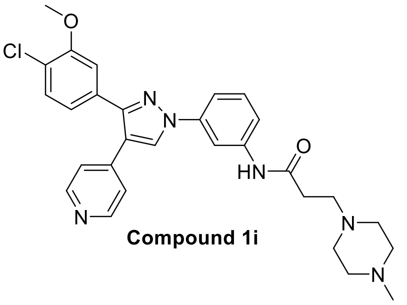
Cl
O
N
N
HN
O
N
N
N

Compound 1i

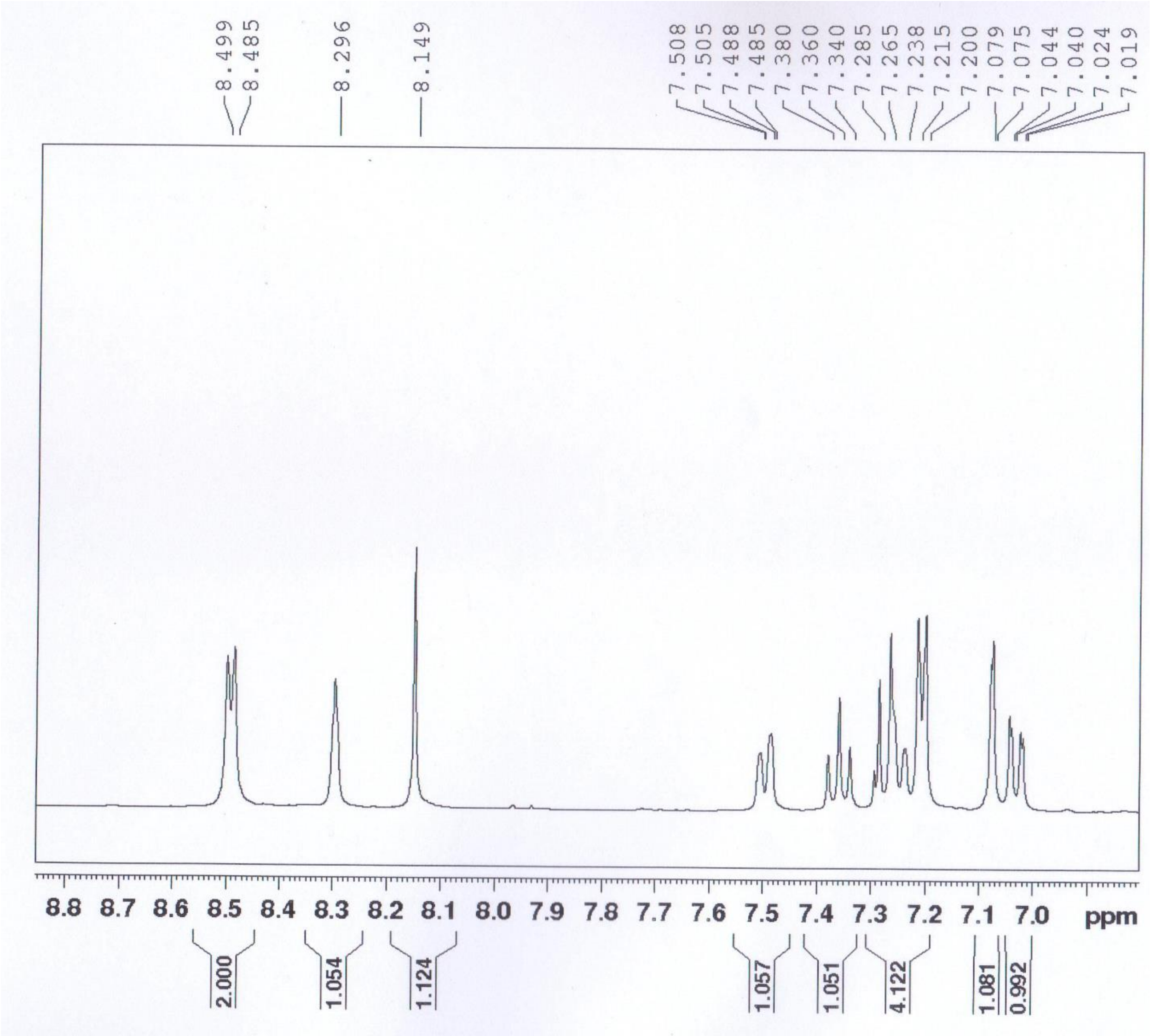
8.499
8.485
8.296
8.149
7.508
7.505
7.488
7.485
7.380
7.360
7.340
7.285
7.265
7.238
7.215
7.200
7.079
7.075
7.044
7.040
7.024
7.019
8.8 8.7 8.6 8.5 8.4 8.3 8.2 8.1 8.0 7.9 7.8 7.7 7.6 7.5 7.4 7.3 7.2 7.1 7.0 ppm
2.000
1.054
1.124
1.057
1.051
4.122
1.081
0.992

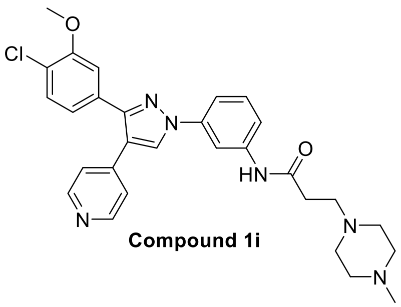

Compound 1i

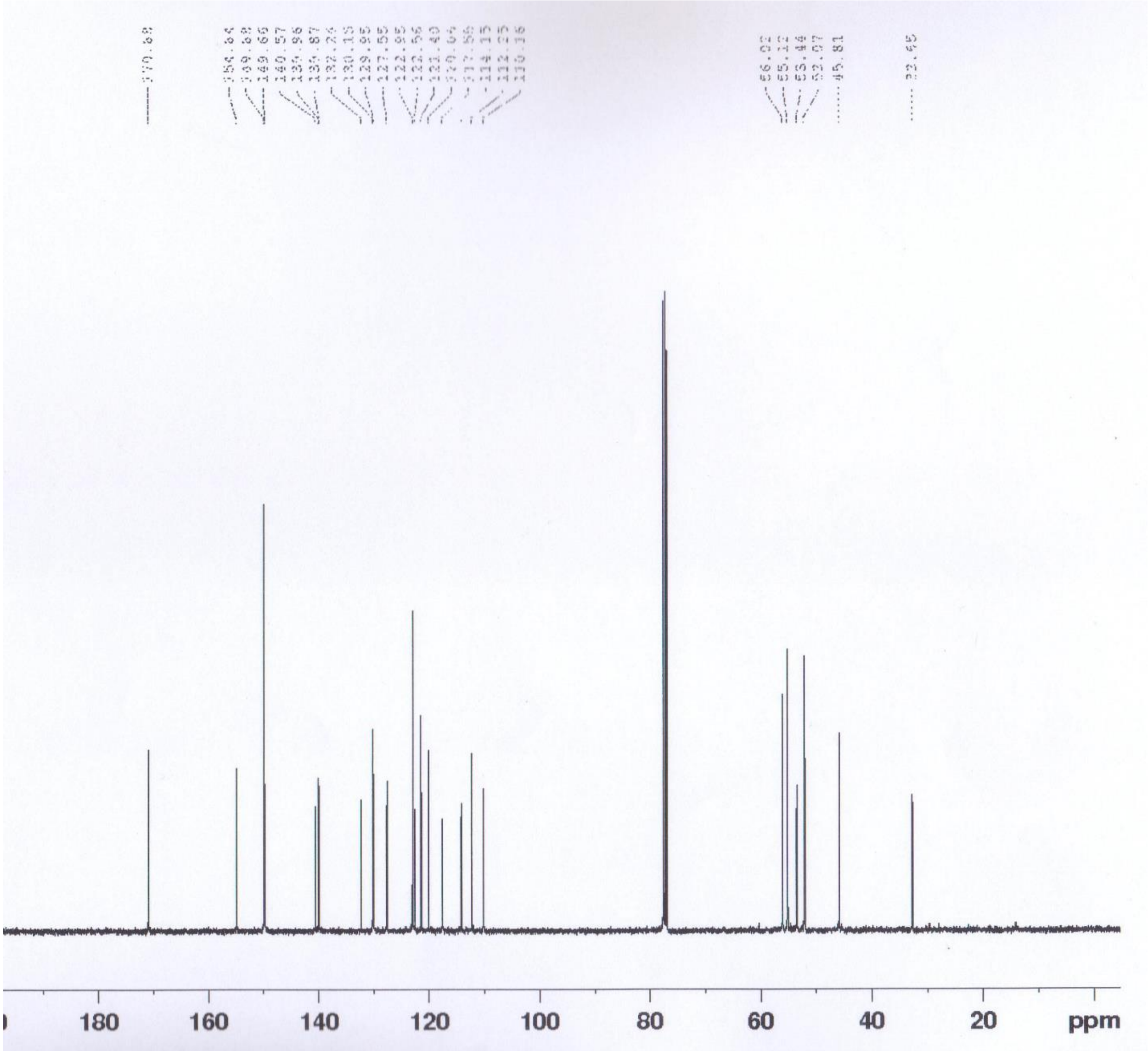

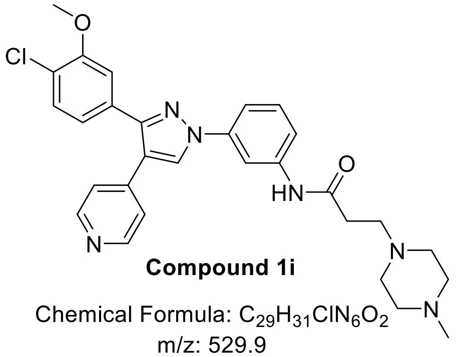

Compound 1i

Chemical Formula: $C_{29}H_{31}ClN_6O_2$

m/z: 529.9

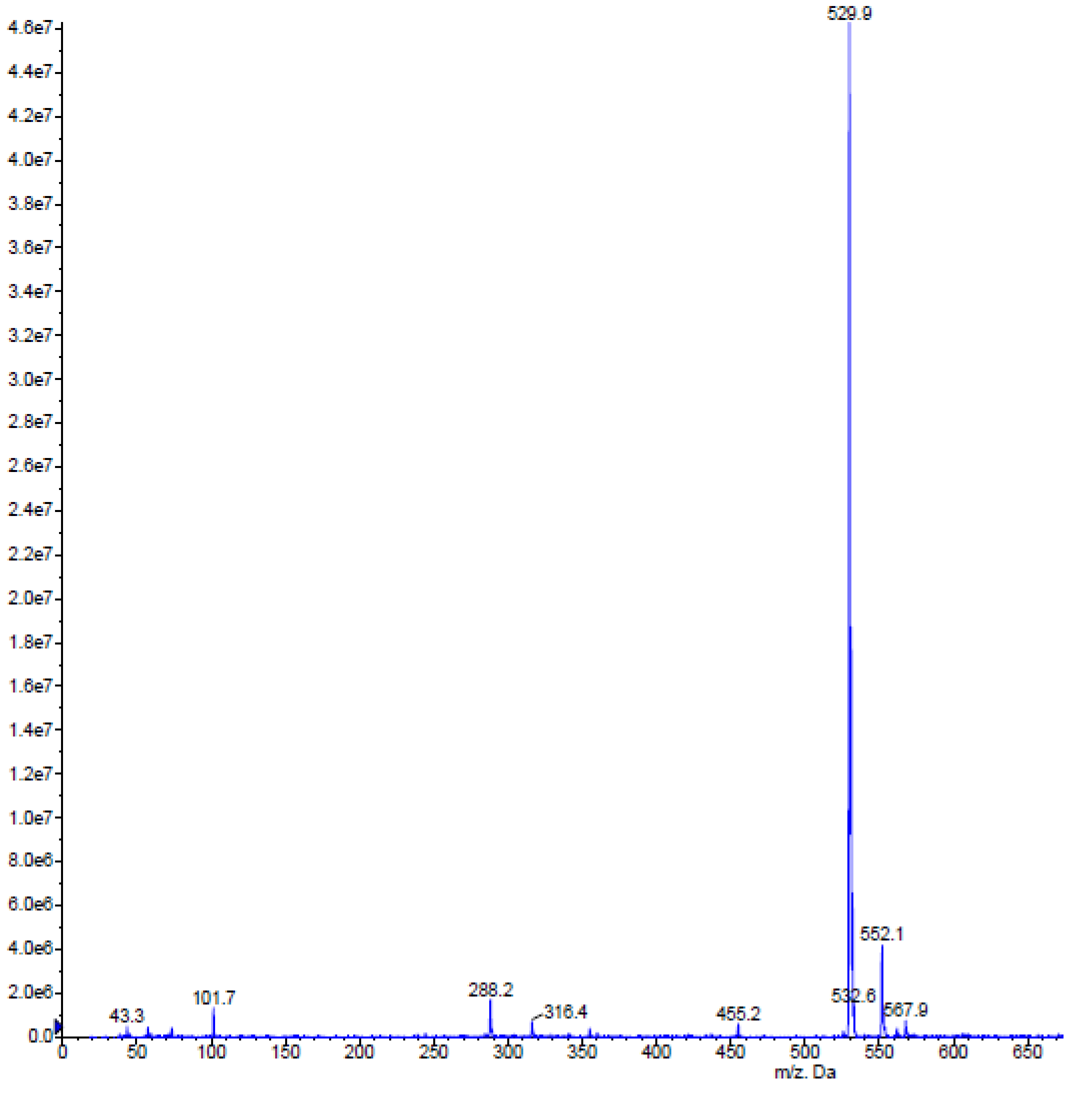

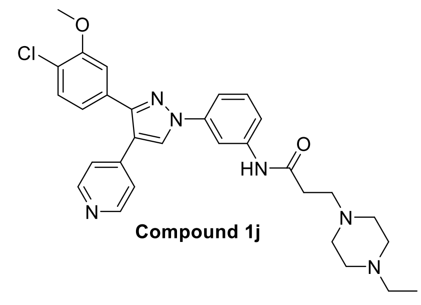
Cl
O
N
N
HN
O
N
N
N

Compound 1j

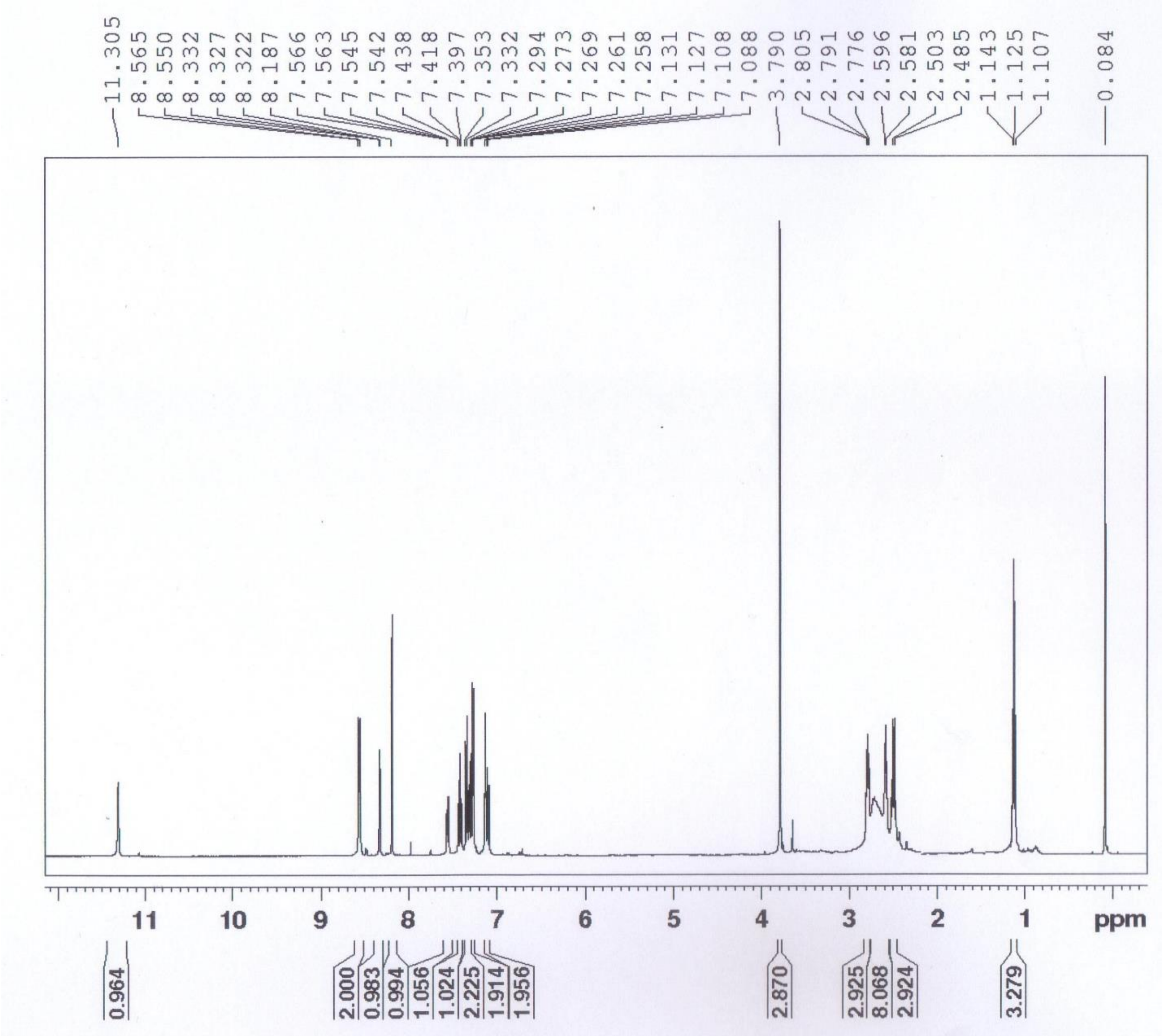
11.305
8.565
8.550
8.332
8.327
8.322
8.187
7.566
7.563
7.545
7.542
7.438
7.418
7.397
7.353
7.332
7.294
7.273
7.269
7.261
7.258
7.131
7.127
7.108
7.088
3.790
2.805
2.791
2.776
2.596
2.581
2.503
2.485
1.143
1.125
1.107
0.084
11
10
9
8
7
6
5
4
3
2
1
ppm
0.964
2.000
0.983
0.994
1.056
1.024
2.225
1.914
1.956
2.870
2.925
8.068
2.924
3.279

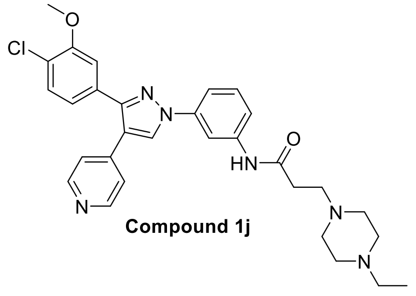

O
Cl
N
N
HN
O
N
N
N

Compound 1j

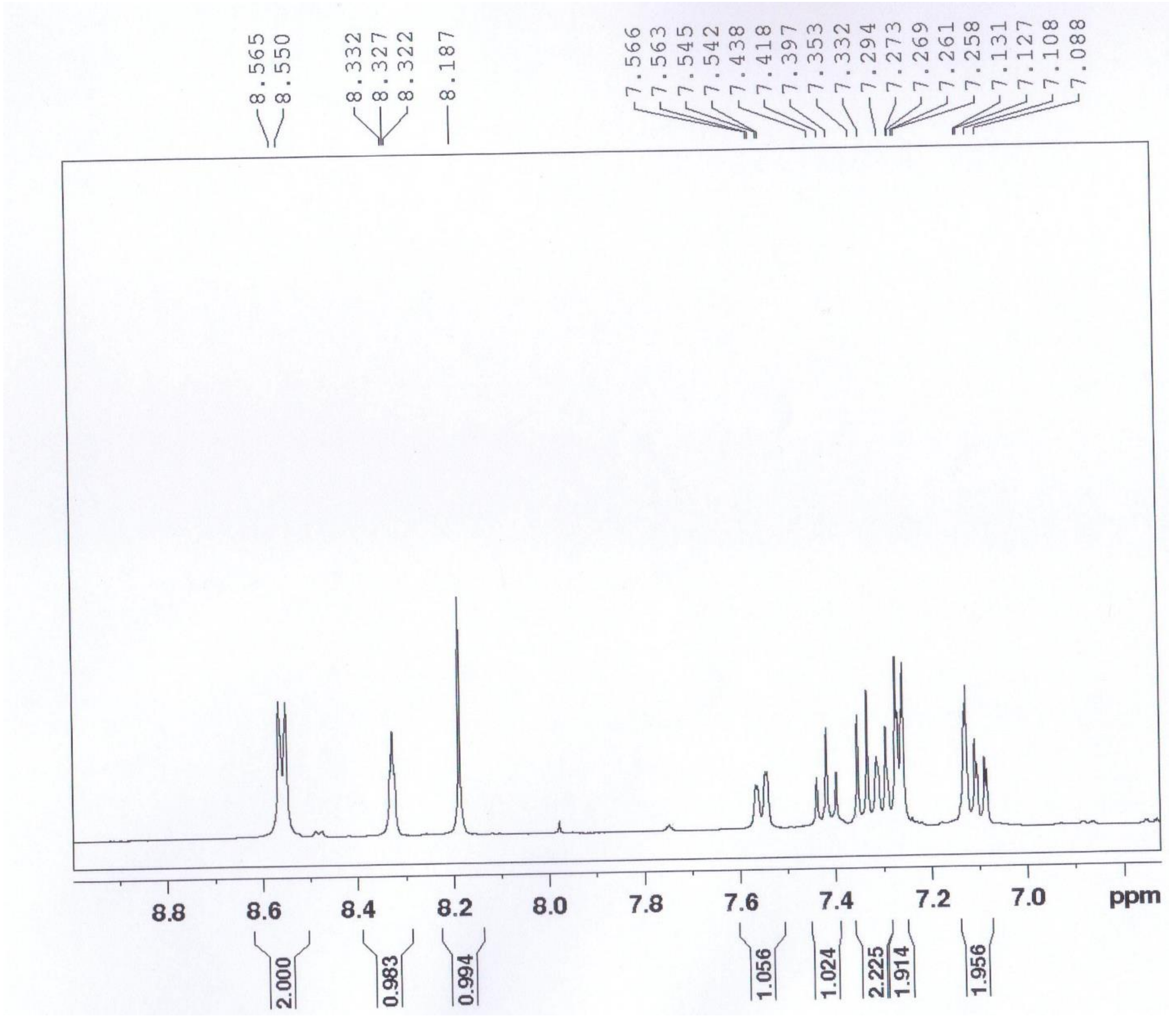

8.565
8.550
8.332
8.327
8.322
8.187
7.566
7.563
7.545
7.542
7.438
7.418
7.397
7.353
7.332
7.294
7.273
7.269
7.261
7.258
7.131
7.127
7.108
7.088
8.8
8.6
8.4
8.2
8.0
7.8
7.6
7.4
7.2
7.0
ppm
2.000
0.983
0.994
1.056
1.024
2.225
1.914
1.956

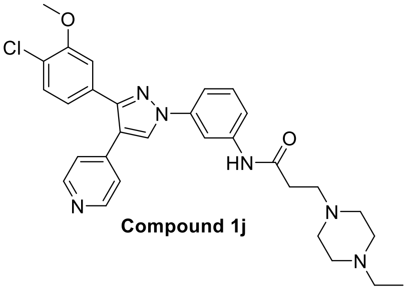

Compound 1j

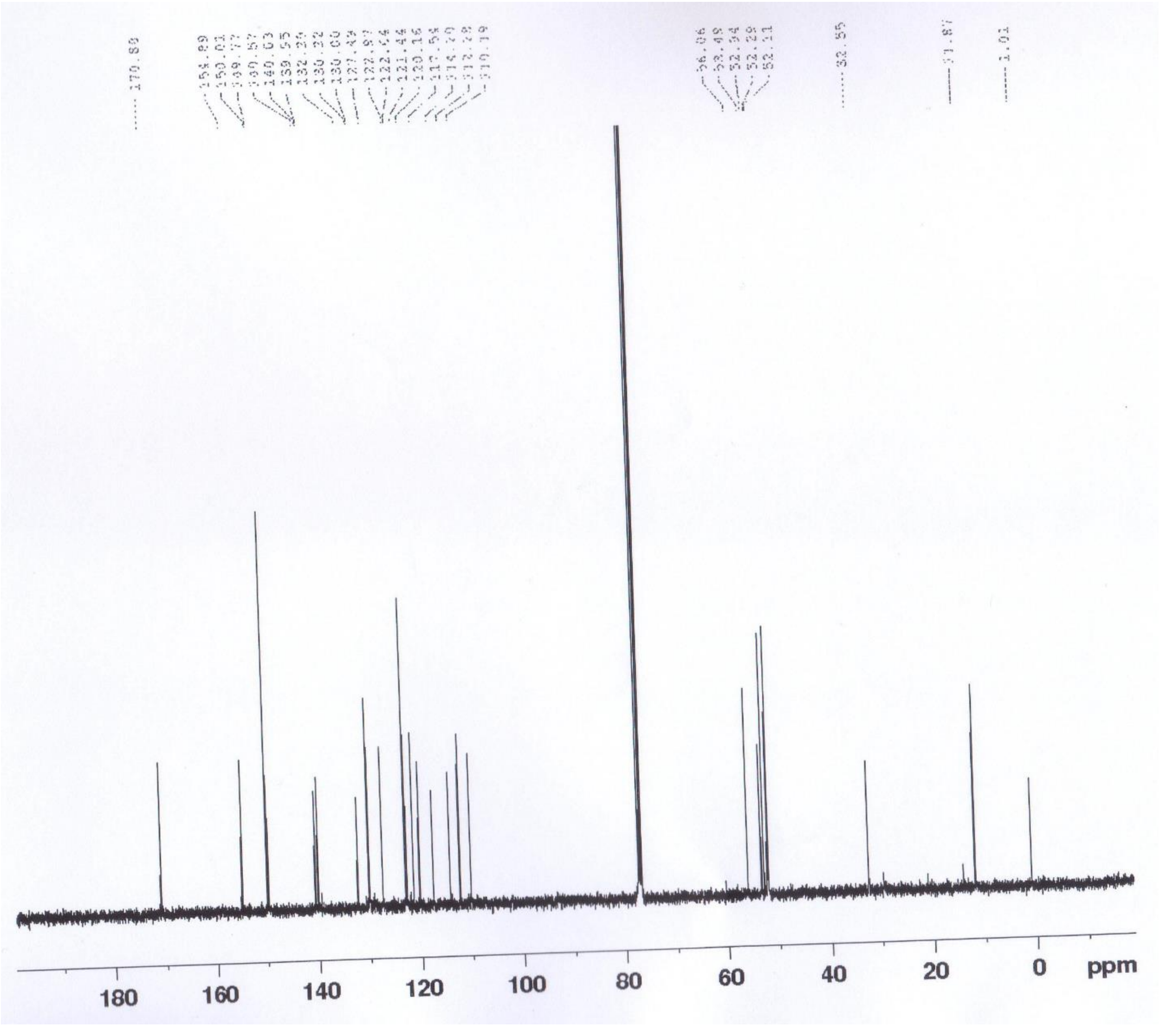

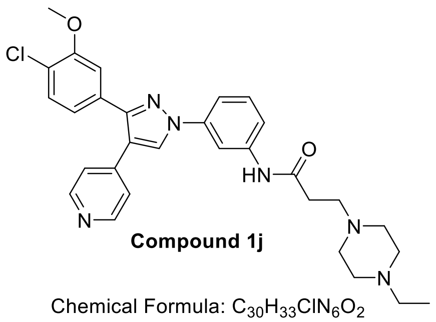

Compound 1j

Chemical Formula: $C_{30}H_{33}ClN_6O_2$
m/z+1: 545.9

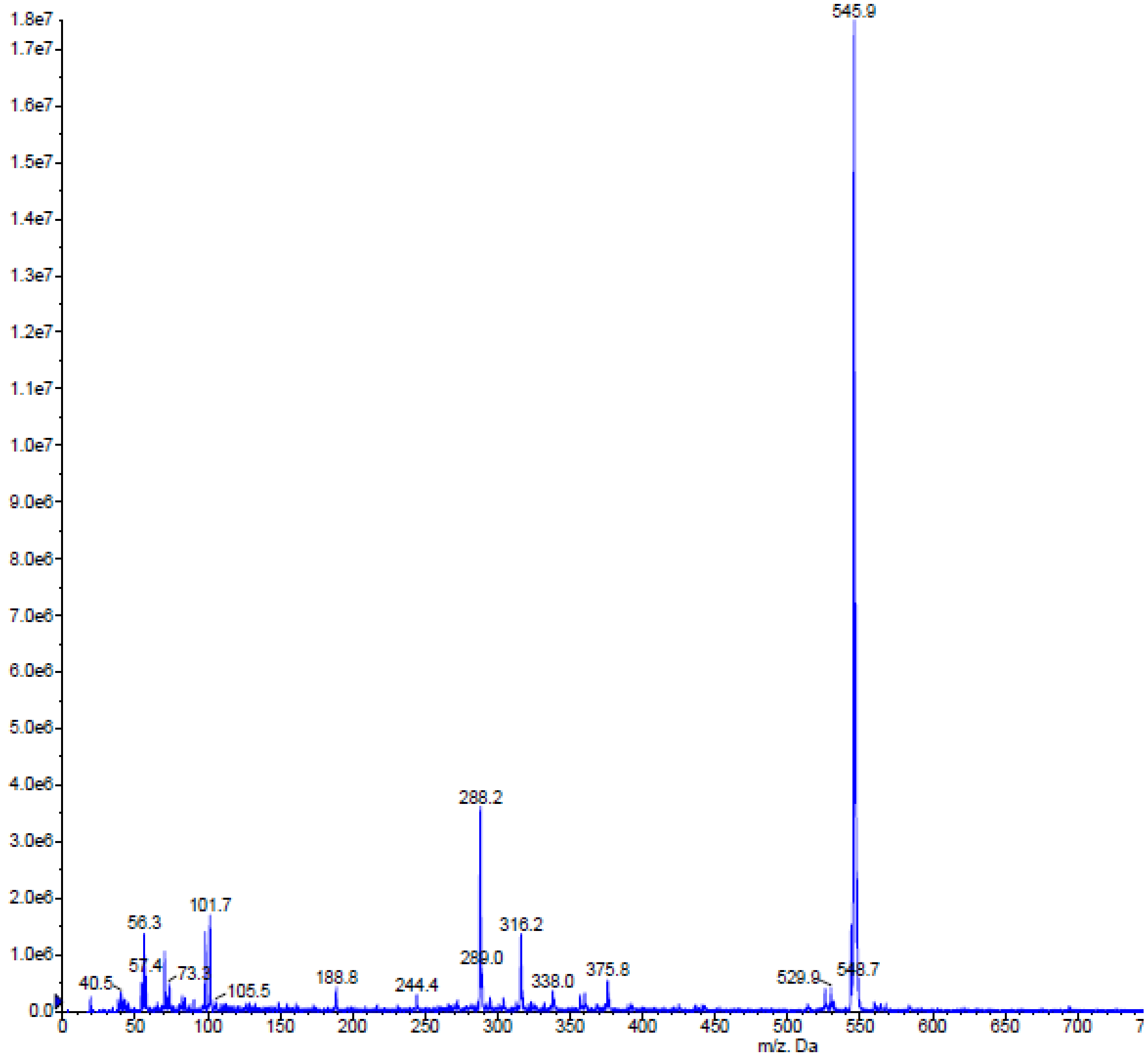

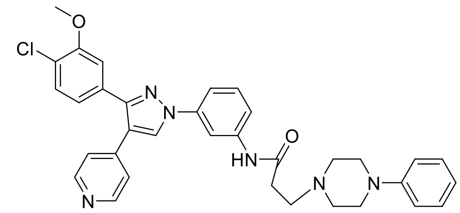

Compound 1K

Exact Mass: 592.24

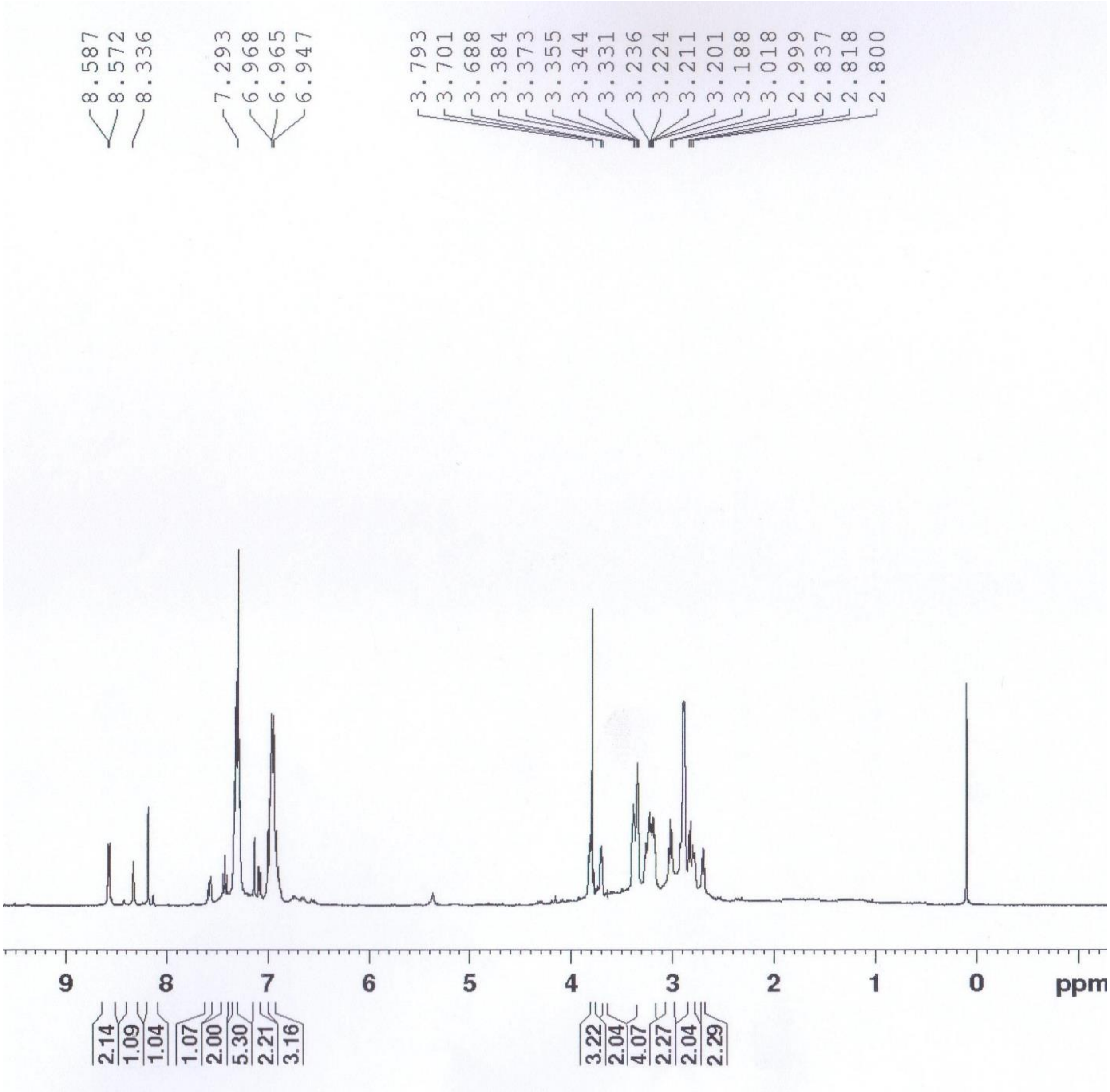

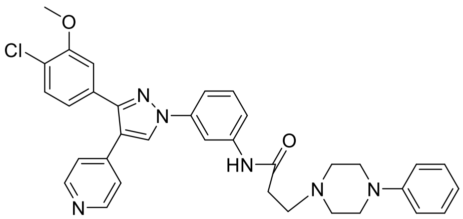

Compound 1K

Exact Mass: 592.24

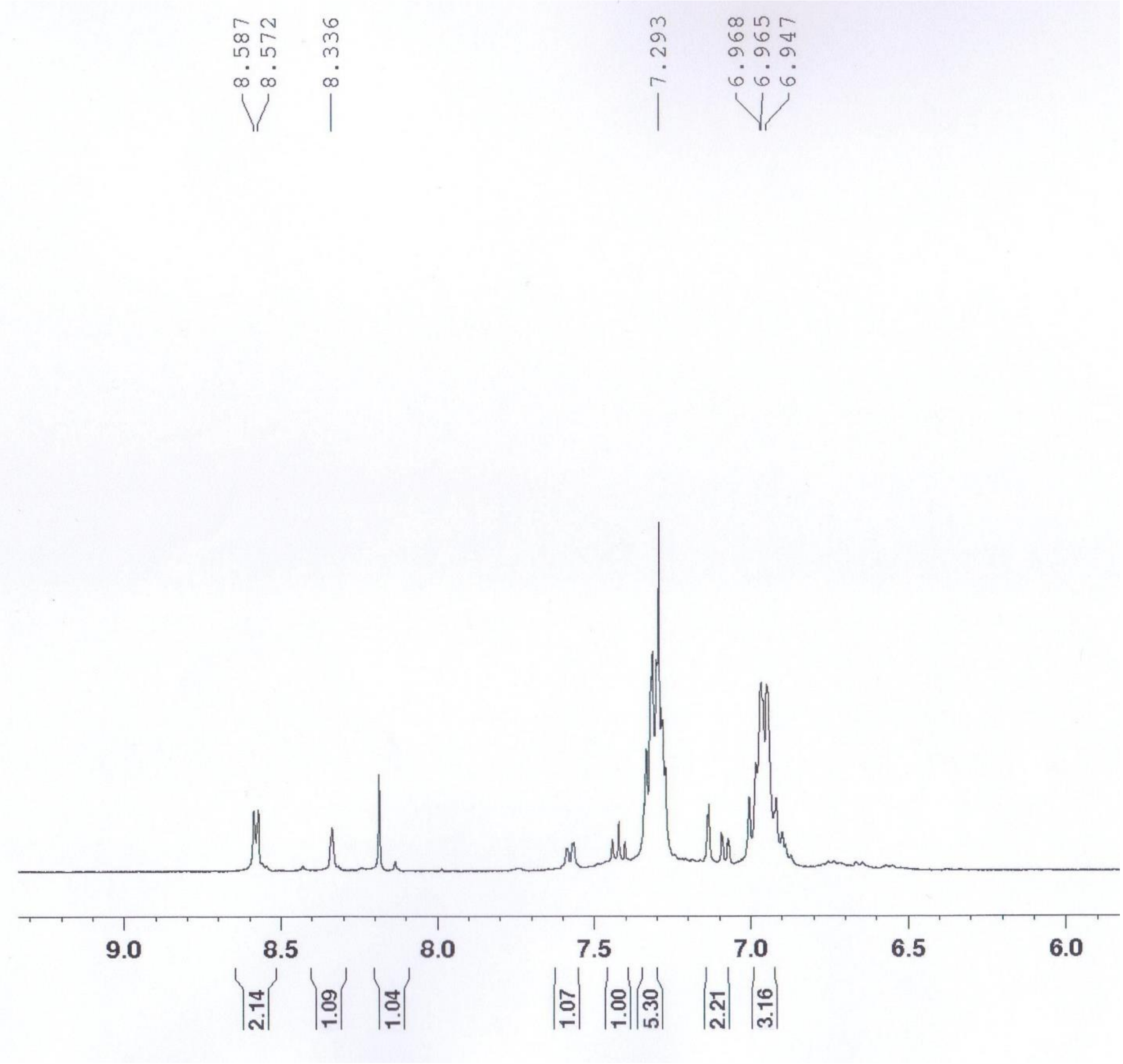

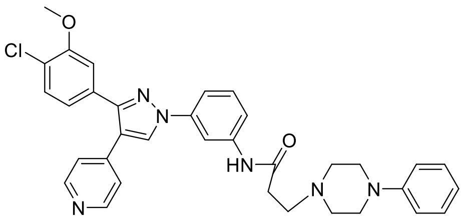

Compound 1K

Exact Mass: 592.24

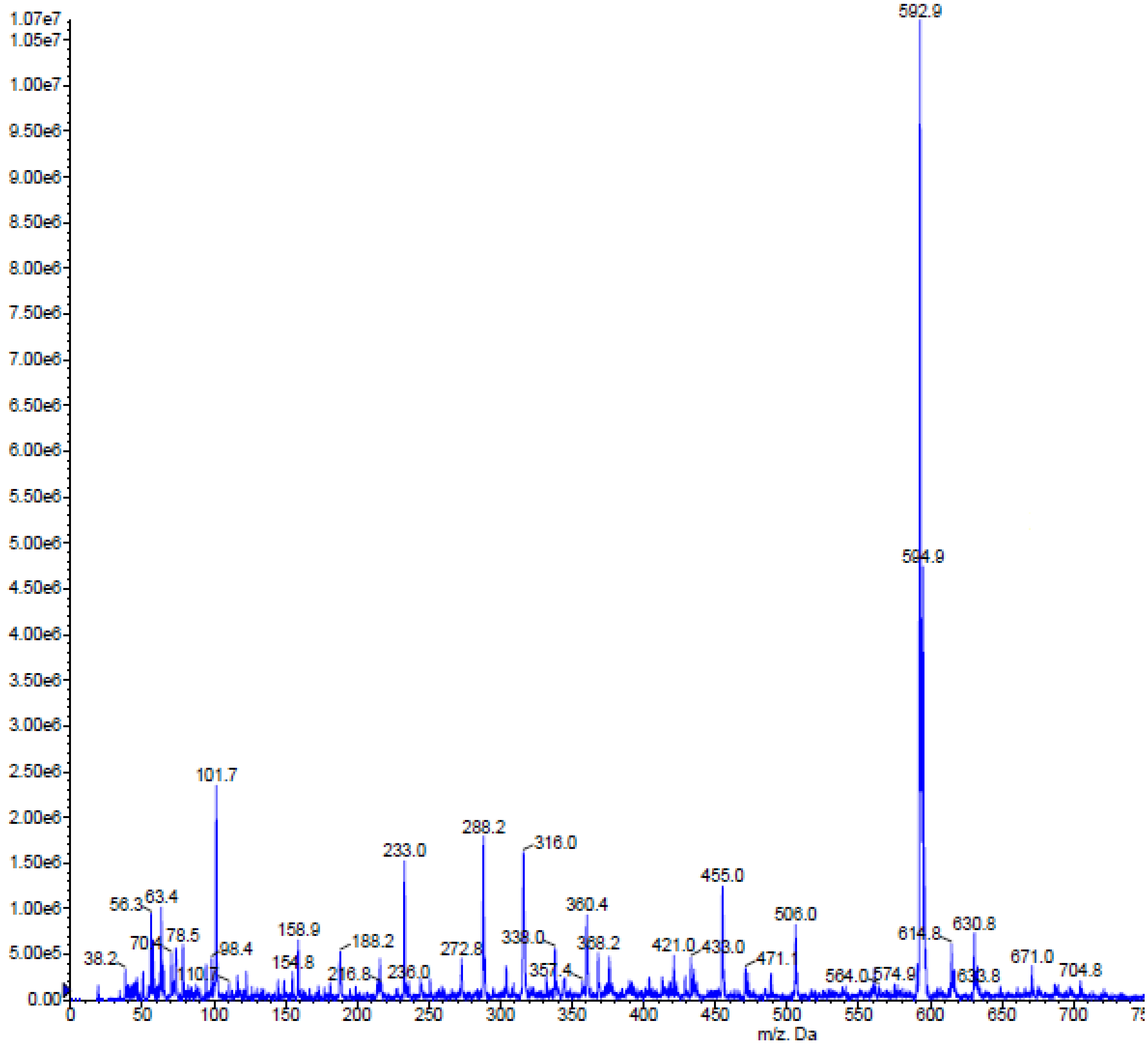

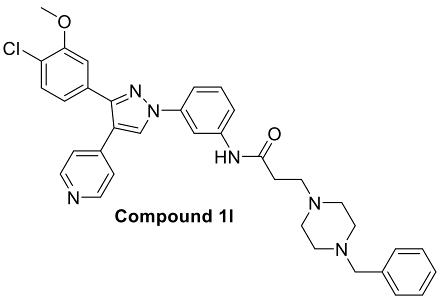

Cl
O
N
N
HN
O
N
N
N

Compound 1l

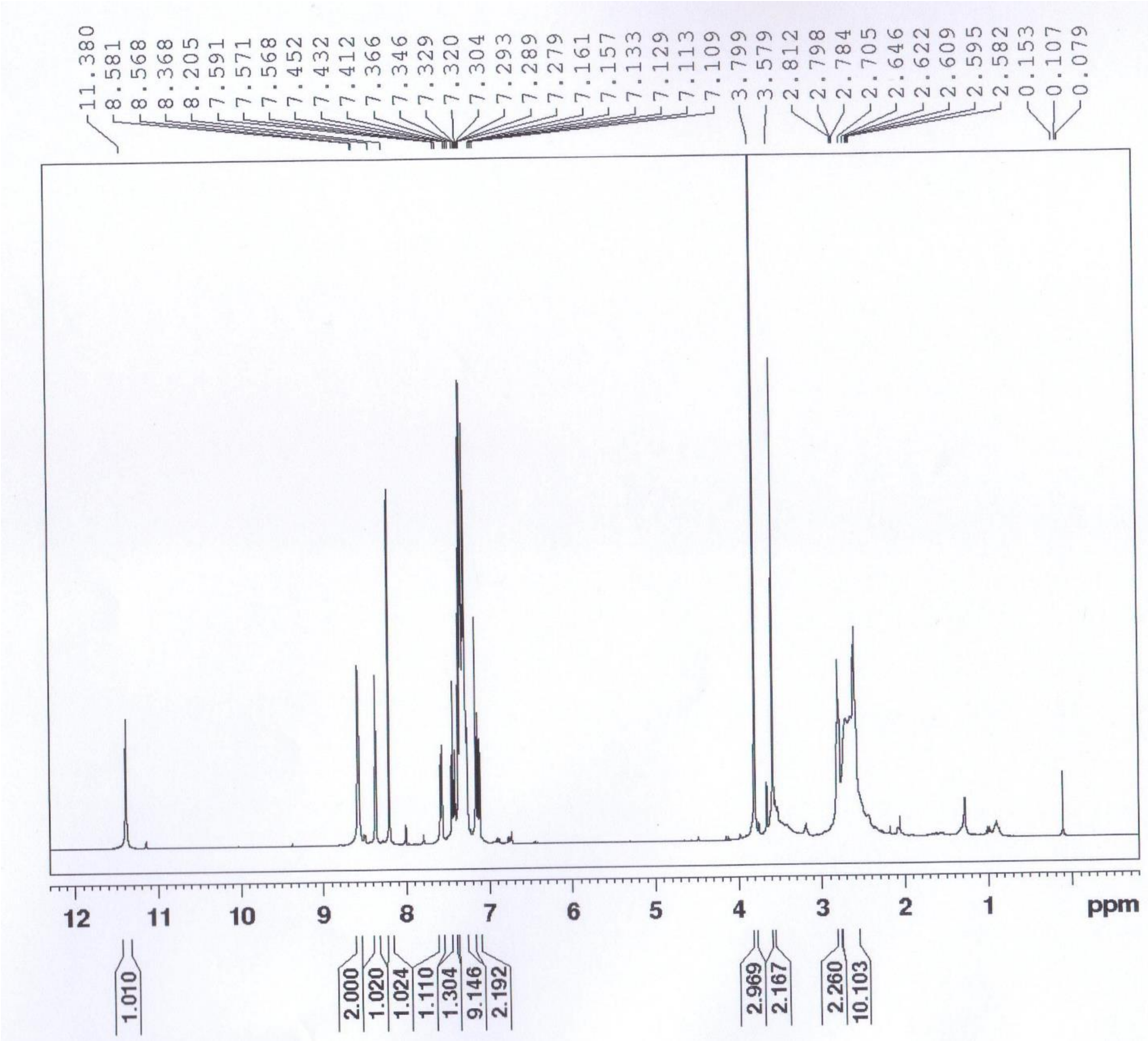

11.380
8.581
8.568
8.368
8.205
7.591
7.571
7.568
7.452
7.432
7.412
7.366
7.346
7.329
7.320
7.304
7.293
7.289
7.279
7.161
7.157
7.133
7.129
7.113
7.109
3.799
3.579
2.812
2.798
2.784
2.705
2.646
2.622
2.609
2.595
2.582
0.153
0.107
0.079
12 11 10 9 8 7 6 5 4 3 2 1 ppm
1.010
2.000
1.020
1.024
1.110
1.304
9.146
2.192
2.969
2.167
2.260
10.103

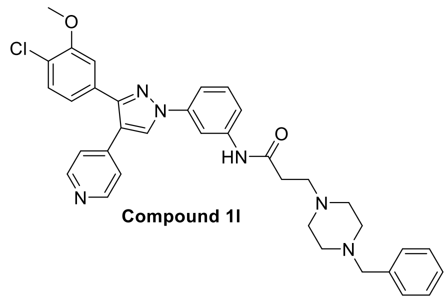
O
Cl
N
N
HN
O
N
N
N

Compound 1l

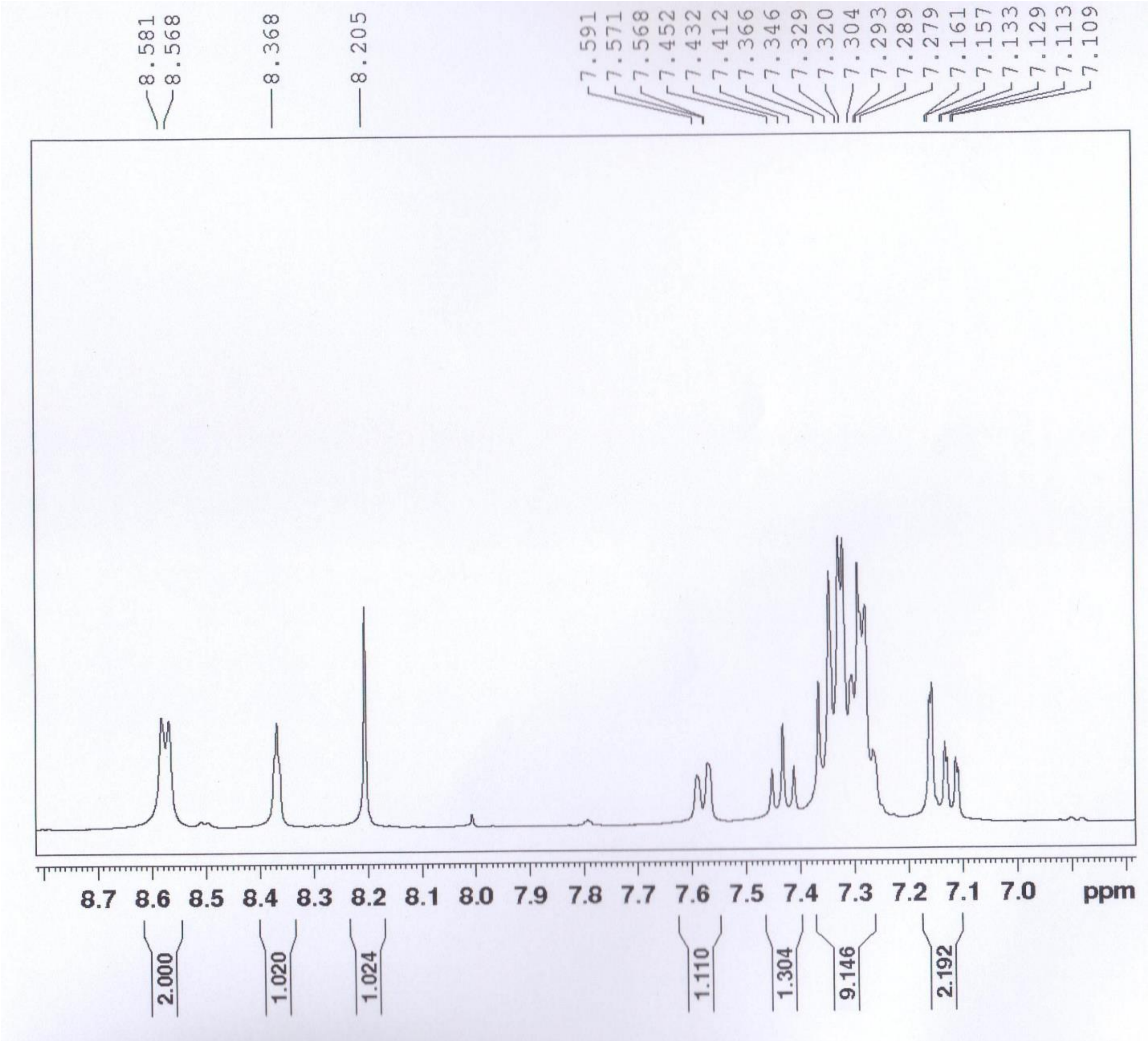
8.581
8.568
8.368
8.205
7.591
7.571
7.568
7.452
7.432
7.412
7.366
7.346
7.329
7.320
7.304
7.293
7.289
7.279
7.161
7.157
7.133
7.129
7.113
7.109
8.7 8.6 8.5 8.4 8.3 8.2 8.1 8.0 7.9 7.8 7.7 7.6 7.5 7.4 7.3 7.2 7.1 7.0 ppm
2.000
1.020
1.024
1.110
1.304
9.146
2.192

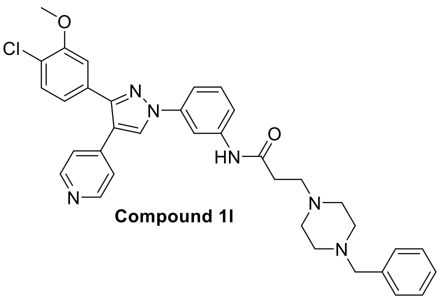

Compound 1l

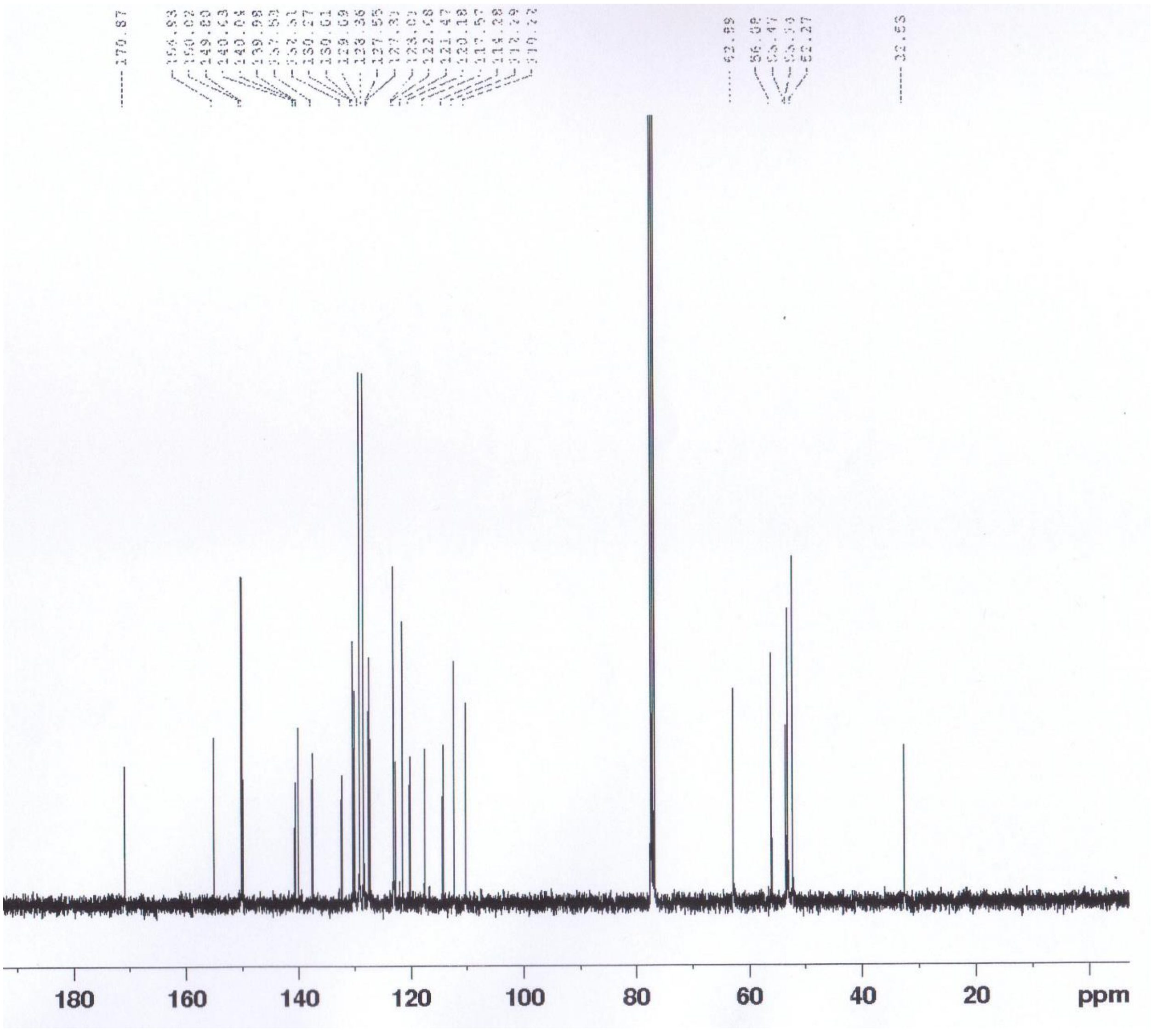

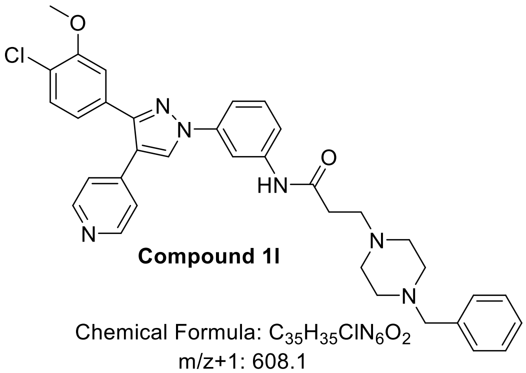

Compound 1l

Chemical Formula: $C_{35}H_{35}ClN_6O_2$
m/z+1: 608.1

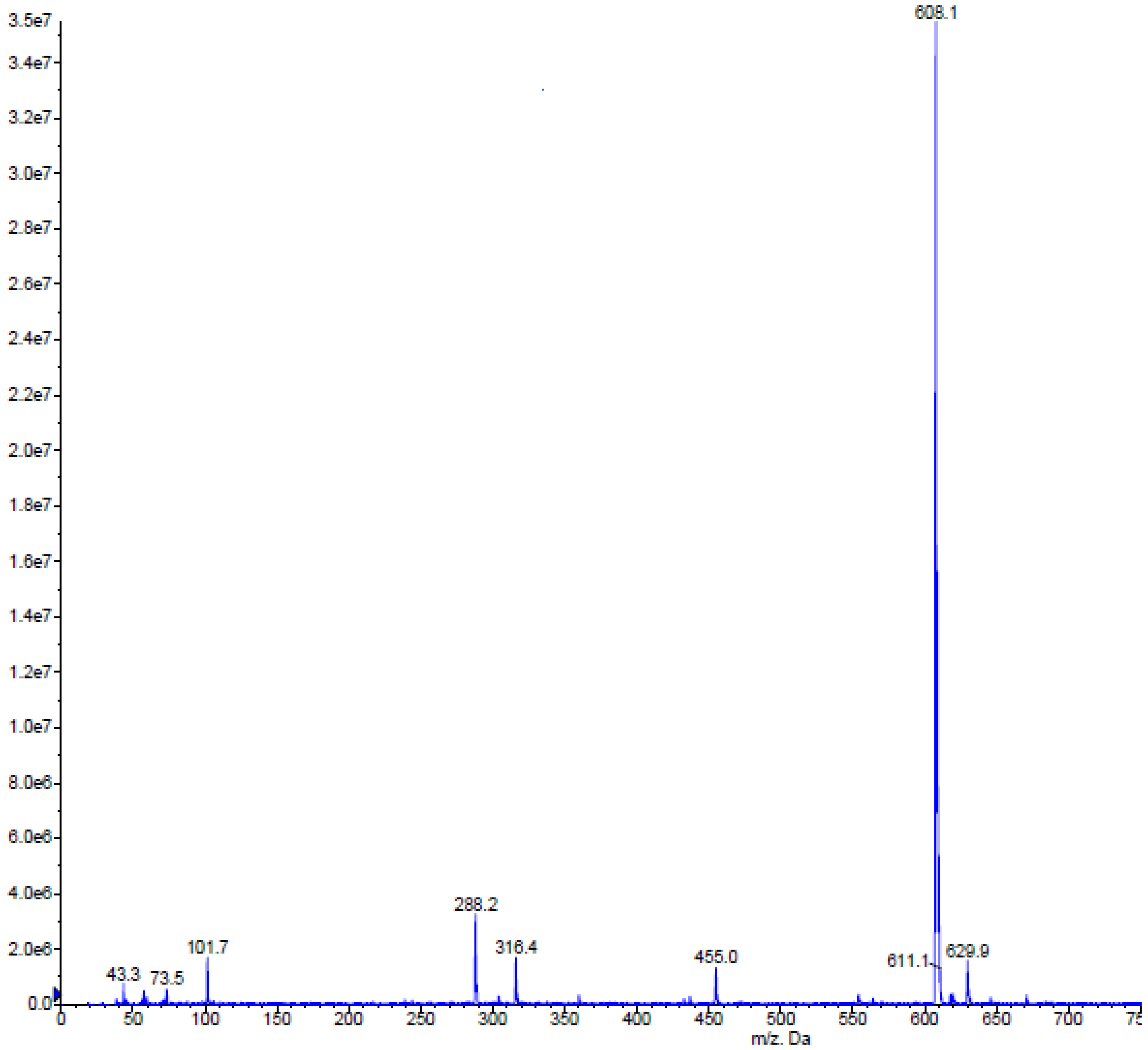

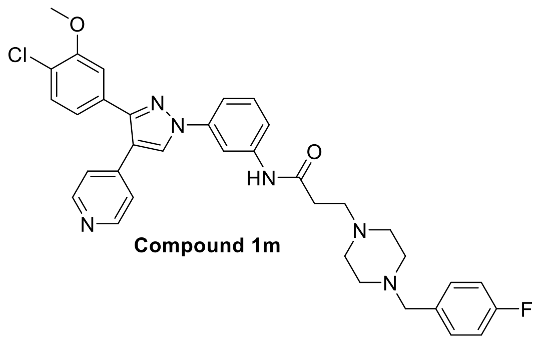
Cl
O
N
N
N
HN
O
N
N
F

Compound 1m

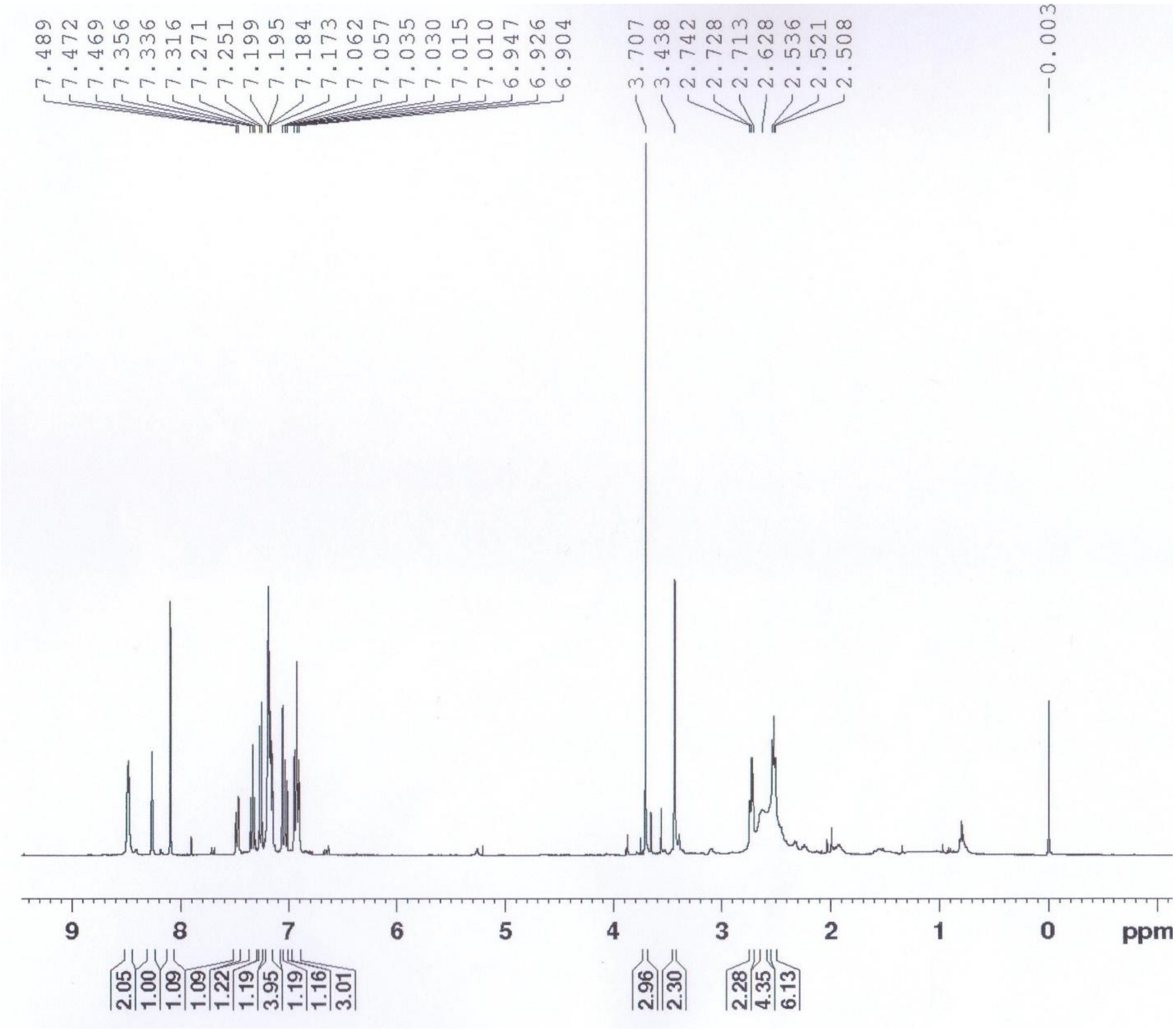
7.489
7.472
7.469
7.356
7.336
7.316
7.271
7.251
7.199
7.195
7.184
7.173
7.062
7.057
7.035
7.030
7.015
7.010
6.947
6.926
6.904
3.707
3.438
2.742
2.728
2.713
2.628
2.536
2.521
2.508
-0.003
9
8
7
6
5
4
3
2
1
0
ppm
2.05
1.00
1.09
1.09
1.22
1.19
3.95
1.19
1.16
3.01
2.96
2.30
2.28
4.35
6.13

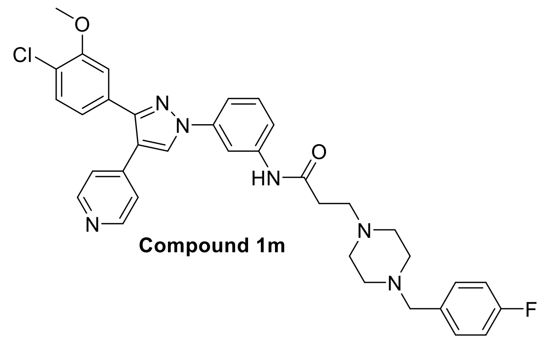
Cl
O
N
N
HN
O
N
N
N
F

Compound 1m

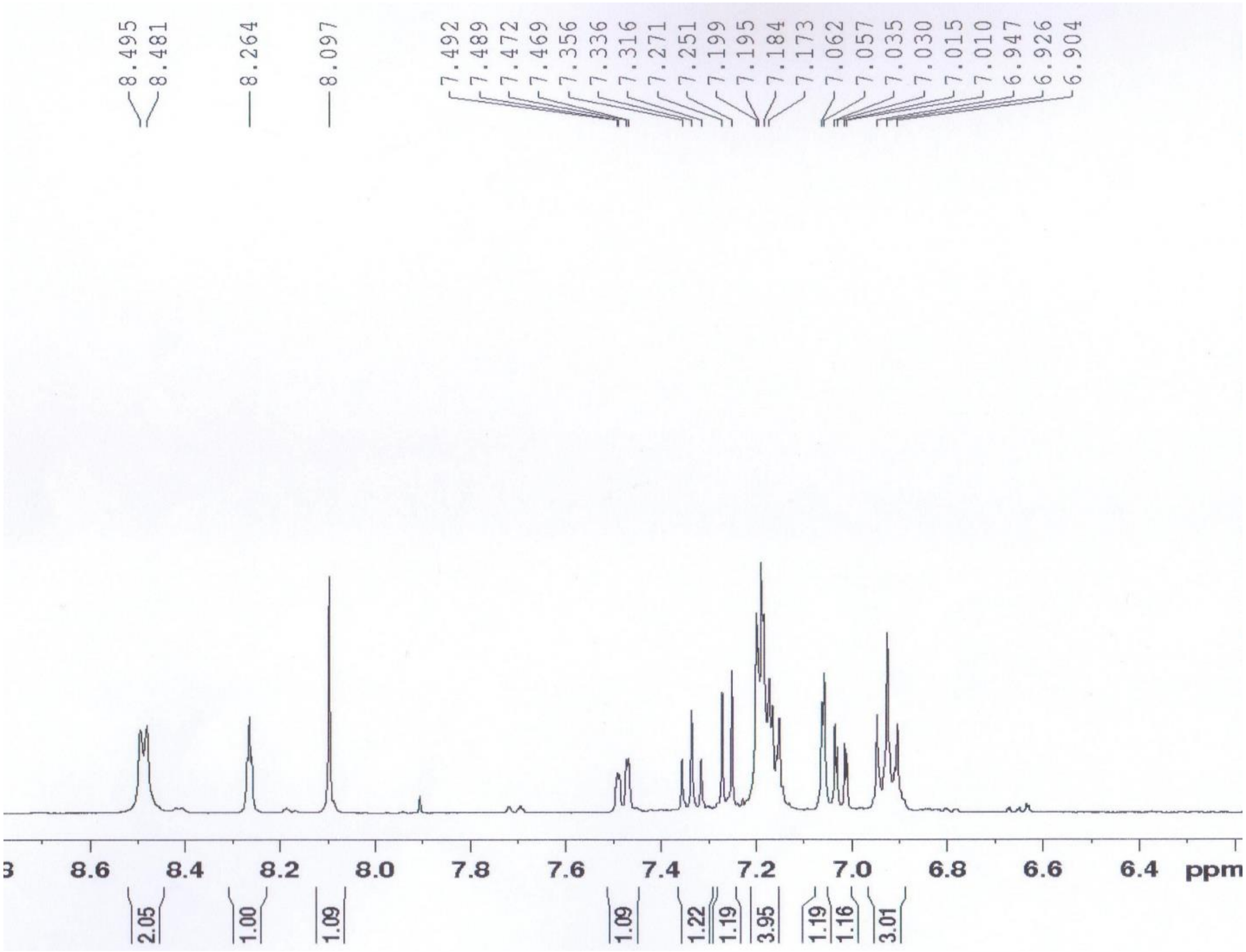
8.495
8.481
8.264
8.097
7.492
7.489
7.472
7.469
7.356
7.336
7.316
7.271
7.251
7.199
7.195
7.184
7.173
7.062
7.057
7.035
7.030
7.015
7.010
6.947
6.926
6.904
8.6
8.4
8.2
8.0
7.8
7.6
7.4
7.2
7.0
6.8
6.6
6.4
ppm
2.05
1.00
1.09
1.09
1.22
1.19
3.95
1.19
1.16
3.01

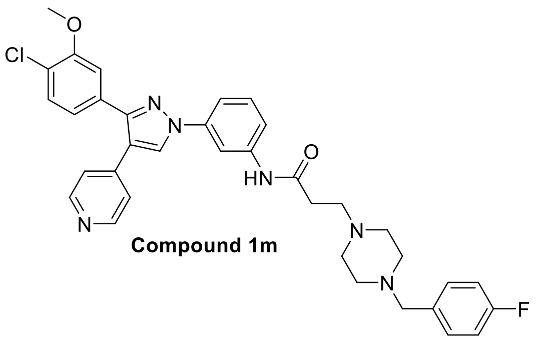

Compound 1m

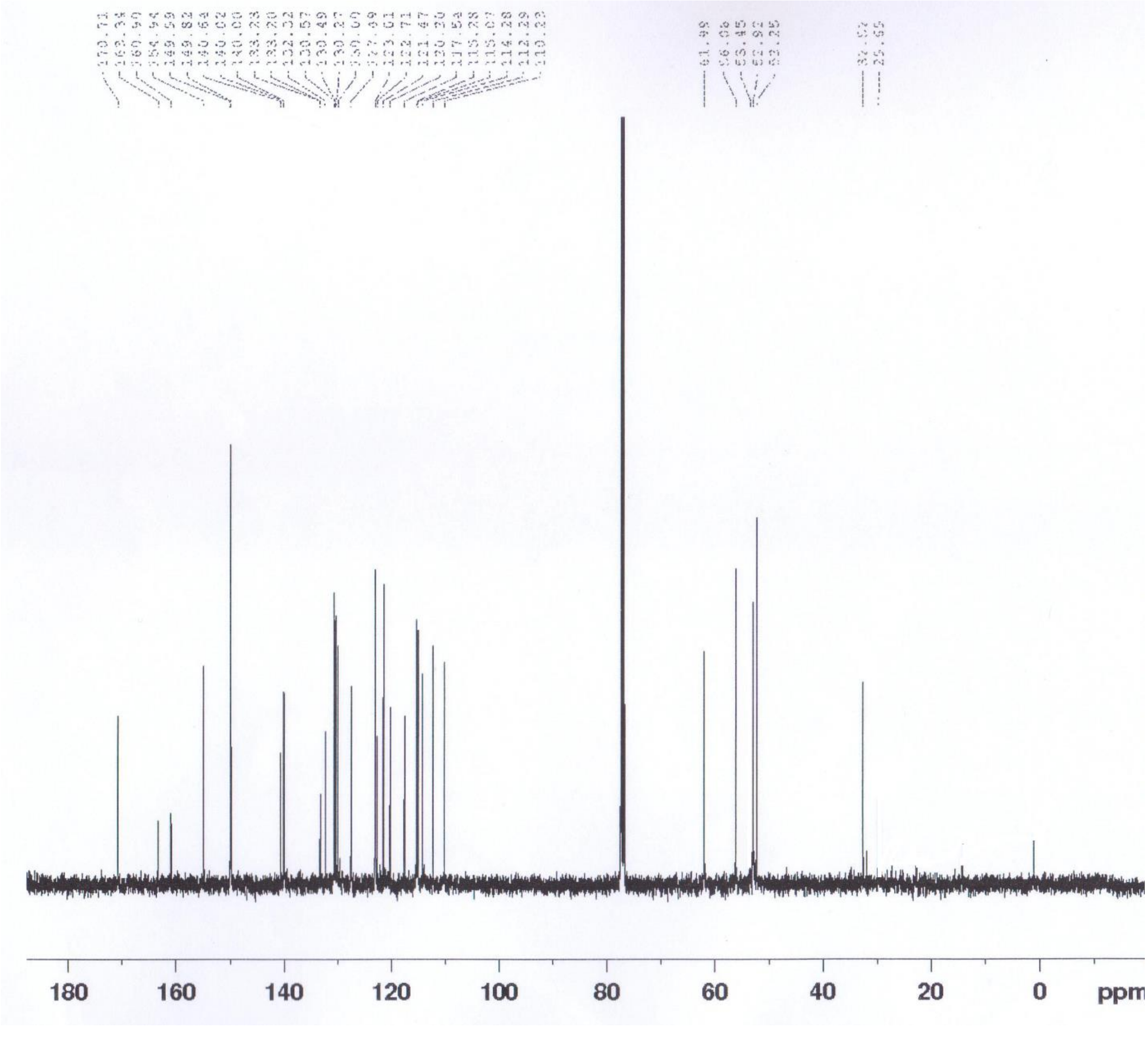

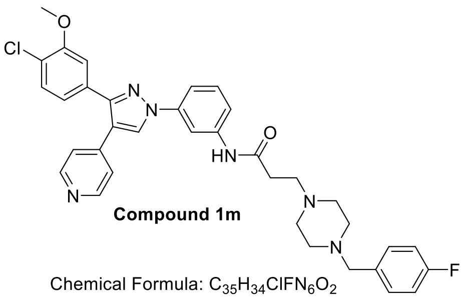

Compound 1m

Chemical Formula: $C_{35}H_{34}ClFN_6O_2$
m/z+2: 626.0

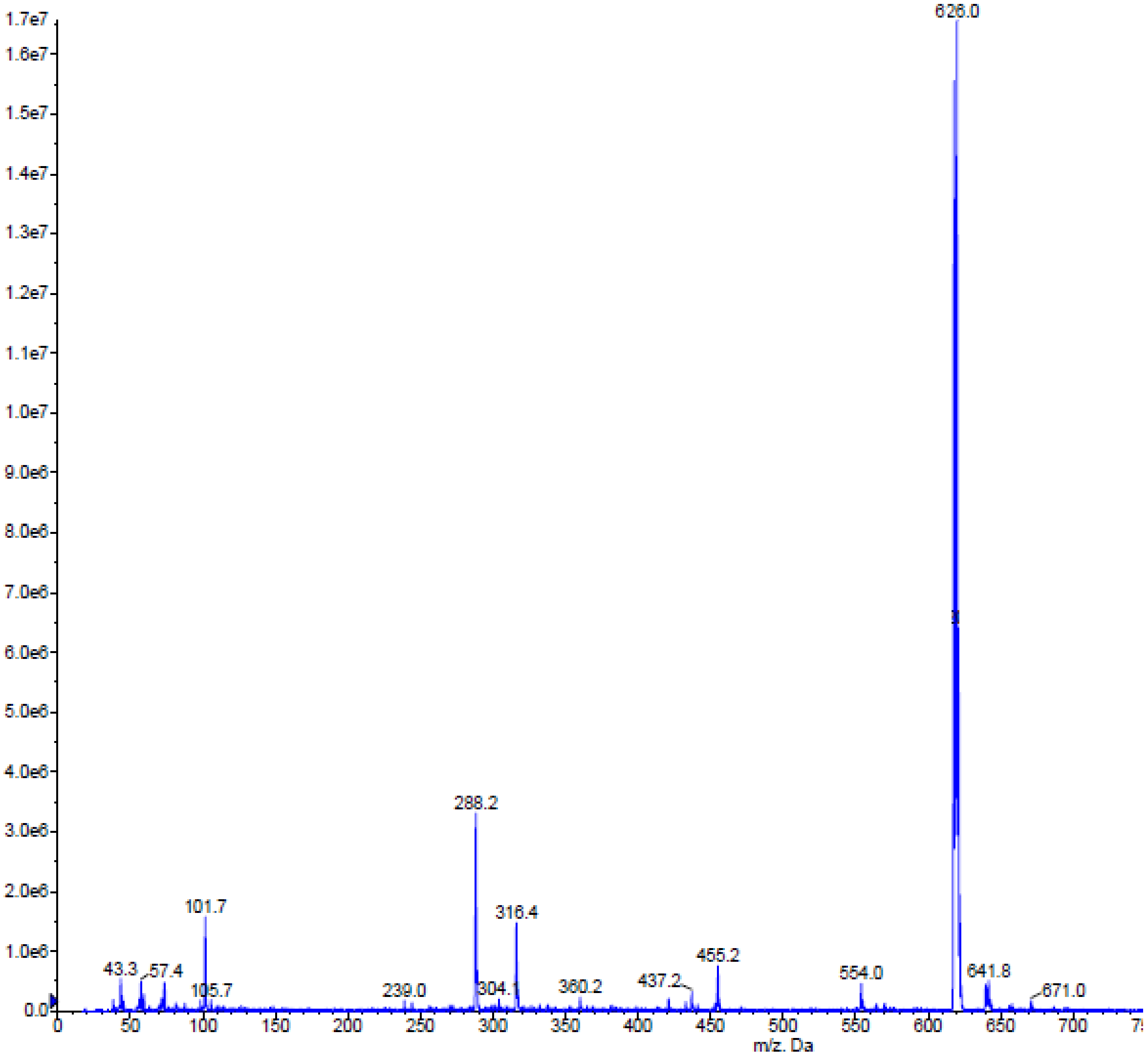

Supplement: Supplementary file 1 [file molecules-26-06489-s001.zip › molecules-1392587-supplementary.pdf]
